# Fine-Tuning Side Chain Substitutions: Impacts on the Lipophilicity–Solubility–Permeability Interplay in Macrocyclic Peptides

**DOI:** 10.3390/md24010013

**Published:** 2025-12-25

**Authors:** Yangping Deng, Hengwei Bian, Hongbo Li, Yingjun Cui, Sizheng Li, Jing Li, Li Chen, Xuemei Zhang, Zhuo Shen, Fengyue Li, Yue Chen, Haohao Fu

**Affiliations:** 1 State Key Laboratory of Medicinal Chemical Biology, Frontiers Science Center for New Organic Matter, College of Chemistry, Nankai University, 94 Weijin Road, Tianjin 300071, China; 18937791883@163.com (Y.D.); bigbian@mail.nankai.edu.cn (H.B.); 1120240554@mail.nankai.edu.cn (S.L.); jinglink@nankai.edu.cn (J.L.); chenliyss@nankai.edu.cn (L.C.); 2 Haihe Laboratory of Sustainable Chemical Transformations, 6 Keyanxi Road, Tianjin 300192, China; 3 Research Center for Analytical Sciences, Tianjin Key Laboratory of Biosensing and Molecular Recognition, College of Chemistry, Nankai University, Tianjin 300071, China; 4 Accendatech Company, Ltd., Tianjin 300193, China; hongbo.li@accendatech.com (H.L.); 1005594816@mail.nankai.edu.cn (Y.C.); xuemei.zhang@accendatech.com (X.Z.); shenzhuo@accendatech.com (Z.S.); fengyue.li@accendatech.com (F.L.)

**Keywords:** macrocyclic ligands, lipophilicity, membrane permeability, free-energy calculation, solubility, click reaction

## Abstract

Macrocyclic drugs are promising for targeting undruggable proteins, including those in cancer. Our prior work identified BE-43547A_2_ (BE) as a selective inhibitor of pancreatic cancer stem cells in PANC-1 cultures, but its high lipophilicity limits clinical application. To address this, we designed derivatives retaining BE’s backbone while modifying tail groups to improve its properties. A concise total synthesis enabled a versatile late-stage intermediate (compound **17**), serving as a platform for efficient diversification of BE analogs via modular click chemistry. This approach introduced a central triazole ring connected by flexible alkyl spacers. Key properties, including lipophilicity, solubility, and Caco-2 permeability, were experimentally determined. These derivatives exhibited reduced lipophilicity and improved solubility but unexpectedly lost cellular activity. Direct target engagement studies using MicroScale Thermophoresis (MST) revealed compound-dependent deactivation mechanisms: certain derivatives retained binding to eEF1A1 with only modestly reduced affinity (e.g., compound **29**), while others showed no detectable binding (e.g., compound **31**). Microsecond-scale molecular dynamics simulations and free-energy calculations showed that, for derivatives retaining target affinity, tail modifications disrupted the delicate balance of drug–membrane and drug–solvent interactions, resulting in substantially higher transmembrane free-energy penalties (>5 kcal/mol) compared to active compounds (<2 kcal/mol). These insights emphasize the need to simultaneously preserve both target engagement and optimal permeability when modifying side chains in cell-permeable macrocyclic peptides, positioning compound **17** as a robust scaffold for future lead optimization. This work furnishes a blueprint for balancing drug-like properties with therapeutic potency in macrocyclic therapeutics.

## 1. Introduction

Pancreatic cancer remains one of the most lethal malignancies worldwide, with a 5-year survival rate below 10% [1]. Traditionally, chemotherapeutic agents such as gemcitabine and 5-fluorouracil have been the cornerstone of pancreatic cancer treatment [2,3]. Yet, their therapeutic impact diminishes considerably in advanced disease stages [4], worsened by the tumor’s hypoxic [5] and nutrient-poor microenvironment, which reduces the efficacy of gemcitabine and kindred agents [6]. Pancreatic cancer stem cells (PCSCs) are believed to play a crucial role in the development, metastasis, and drug resistance associated with pancreatic cancer. Targeting cancer stem cells holds the potential to revolutionize cancer treatment [7]. Macrocyclic peptides have emerged as a promising therapeutic modality, attracting interest from pharmaceutical companies worldwide [8,9]. Their ability to engage targets with high specificity makes them particularly effective against flat protein surfaces [10]. Several macrocyclic peptides have already demonstrated potent antitumor activity across diverse cancer types. Among fully peptidic marine-derived macrocycles, halipeptin D exhibited strong in vitro inhibitory activity against the human colon cancer HCT-116 cell line [11], and phakellistatin **13** displayed potent growth inhibition against the human hepatoma BEL-7404 cell line [12], marking it as a promising lead compound for antitumor agents. However, they occupy the “beyond-rule-of-5” (bRo5) space, leading to poor membrane permeability [13]. Despite these challenges, several exceptions exist, particularly among natural products [14]. For example, the well-studied cyclic undecapeptide cyclosporin A shows surprisingly high passive membrane permeability [15], serving as a rare example of a cyclic peptide that inhibits intracellular targets [16]. Yet, its low solubility poses formulation challenges, illustrating a typical hurdle in drug development under such physicochemical constraints [17]. In addition, the few cyclic peptides that cross cell membranes [18,19,20,21,22,23] often exhibit high lipophilicity, which increases the risk of development failure due to reduced target specificity, higher plasma protein binding, greater volume of distribution, increased toxicity, high clearance, and lower aqueous solubility [24,25,26].

The BE-43547 family of compounds was isolated from the marine sponge-derived *Streptomyces* sp. (strain A43547) by Nishioka and colleagues [27]. BE-43547A_2_ is a macrocyclic depsipeptide, with congeners A1, B1, B2, B3, C1, and C2 that differ in the lipophilic side chain [28]. We recently reported the first asymmetric total synthesis of this cyclic depsipeptide in 15 linear steps [29], together with the identification of eukaryotic translation elongation factor 1α1 (eEF1A1) as a promising therapeutic target for pancreatic cancer [30]. Our findings indicate that BE demonstrates superior selectivity for PCSCs compared to other compounds targeting these cells. Importantly, BE demonstrates unprecedented hypoxia-selective cytotoxicity towards cancer cells. However, BE faces similar issues as other macrocyclic peptides, including high lipophilicity, low solubility, and hemolytic toxicity [31]. Thus, this study aims to address these limitations through structural modifications. We previously demonstrated that the cyclic peptide backbone is essential for BE to maintain its antiproliferative activities against PANC-1 cells [30]. Therefore, the most plausible site for modification lies in the lipid tail. Here, we investigate the effects of side-chain functionality on membrane permeability and various physicochemical properties in bRo5 BE macrocycles. Yet, two key challenges remain in the design of BE derivatives: (i) synthesizing an intermediate that allows easy modification of the tail, and (ii) understanding how the lipid tail influences the antiproliferative activity against pancreatic cancer cell lines of the drug molecules.

In this study, we aim to address the two aforementioned challenges. For the first challenge, concerns about the synthetic complexity of macrocyclic drugs, which may hinder lead optimization and raise scale-up costs [32], have made the pharmaceutical industry cautious in their development. As a result, macrocycles are often excluded from academic and industrial screening libraries. Here, we present a synthesized BE derivative scaffold with a short tail and a carbon-carbon bond, enabling easy extension with chain-like molecules to fine-tune the physicochemical properties of BE derivatives. To investigate how the property of the tail influences the activity of BE, we modified its hydrophilicity and hydrophobicity, synthesizing a series of new compounds. Our findings revealed that these modified compounds lost the original antiproliferative activity against pancreatic cancer cell lines. To uncover the mechanism behind this deactivation at the atomic level, we performed molecular dynamics simulations and free-energy calculations. Based on these results, we propose key considerations for the future design of this class of drugs.

## 2. Results

### 2.1. Design of a Key Intermediate for BE Derivatives

**Figure 1:**
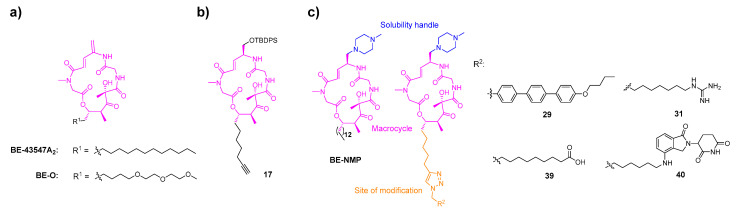
Design of a key intermediate for BE derivatives. (**a**) The structures of BE-43547A_2_ and BE-O. (**b**) The presented intermediate **17**. (**c**) BE derivatives presented in this study. The conserved macrocyclic core is highlighted in pink, the tail region (site of structural modification) is highlighted in orange, and the methylpiperazinyl group is highlighted in blue.

In our previous study, we designed a molecular probe and showed that BE exerts its anticancer effects by specifically and covalently binding to cysteine234 of eEF1A1 through its macrocycle [30]. In addition to BE, its derivatives BE-O and BE-NMP were also synthesized (Figure 1). As stated in a previous study, the macrocycle of BE is crucial [30]. Therefore, we retain the majority of the macrocycle unchanged and introduced a key intermediate, **17** (Figure 1b), featuring a short tail with a terminal alkyne that can be easily extended via a click reaction [33] with azide-containing chain-like molecules. Additionally, a methylpiperazinyl group, which does not affect the activity of the macrocycle [31], was introduced to enhance the solubility of BE derivatives. To demonstrate the versatility of **17** in connecting to different types of tails, we designed several derivatives with varying tail properties.

We noted that the approved antifungal drug anidulafungin is developed through the structural optimization of echinocandin B, where the replacement of its side chain with an alkoxytriphenyl group significantly reduces the hemolytic toxicity associated with echinocandin B [34]. Given that our BE cyclic peptides also exhibit hemolytic toxicity, we drew inspiration from this strategy and introduced an alkoxytriphenyl side chain into the BE scaffold, leading to the design and synthesis of **29** (Figure 1c). The guanidinium group, known for its basic and hydrophilic nature, plays a critical role in enhancing cellular uptake due to its ability to form hydrogen bonds and interact with cell membranes [35,36], while the carboxyl group introduces an ionizable, polar functionality that can modulate physicochemical properties. To systematically investigate the influence of key physicochemical properties, specifically hydrogen bond donors (HBDs), polar surface area (PSA) and lipophilicity (log *D*_7.4_) on the membrane permeability and bioactivity of BE cyclic peptides, we designed and synthesized **31** and **39** by incorporating a guanidinium group or a polar carboxyl group into the side chain. In addition, to explore the potential of BE cyclic peptides as a scaffold for targeted protein degradation, we introduce a Cereblon-containing proteolysis-targeting chimera moiety in compound **40**. All the aforementioned modifications are described in Figure 1.

### 2.2. Synthesis of BE Derivative

**Scheme 1:**
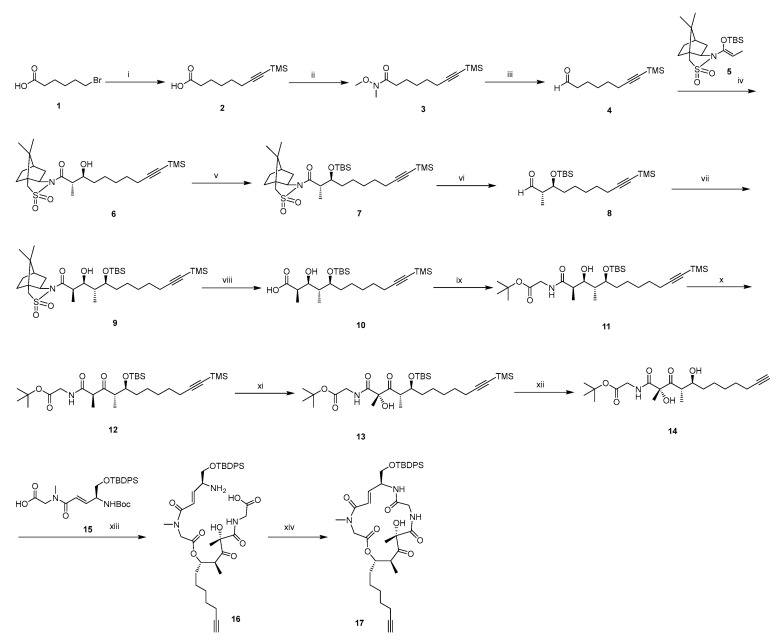
Synthesis of intermediate **17**^a^.

**Scheme 2:**
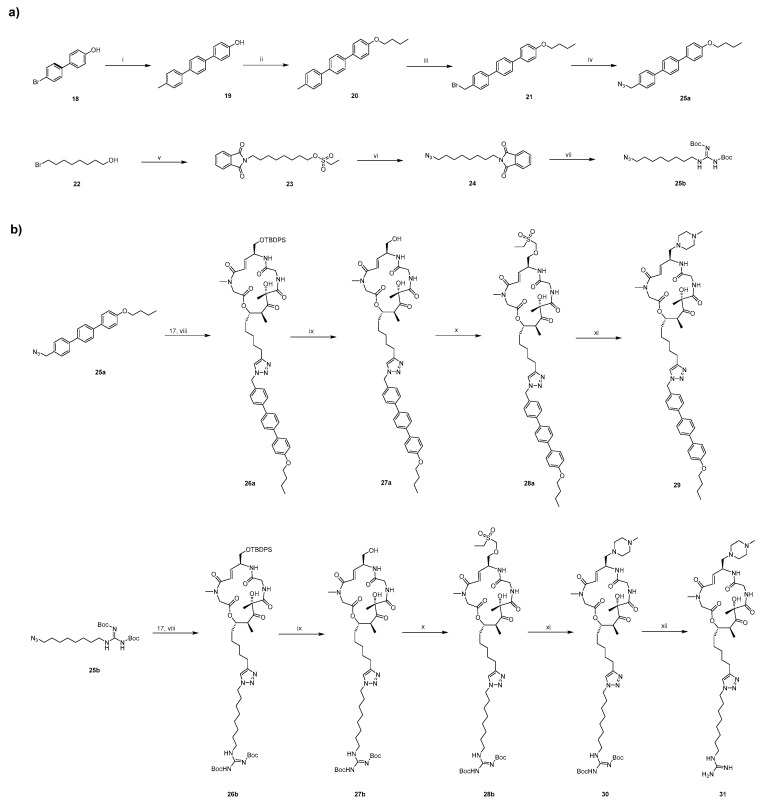
Synthesis of Target Compounds **29** and **31**^a^. (**a**) Synthesis of azide-containing tail fragments; (**b**) Assembly of target compounds via click reaction.

**Scheme 3:**
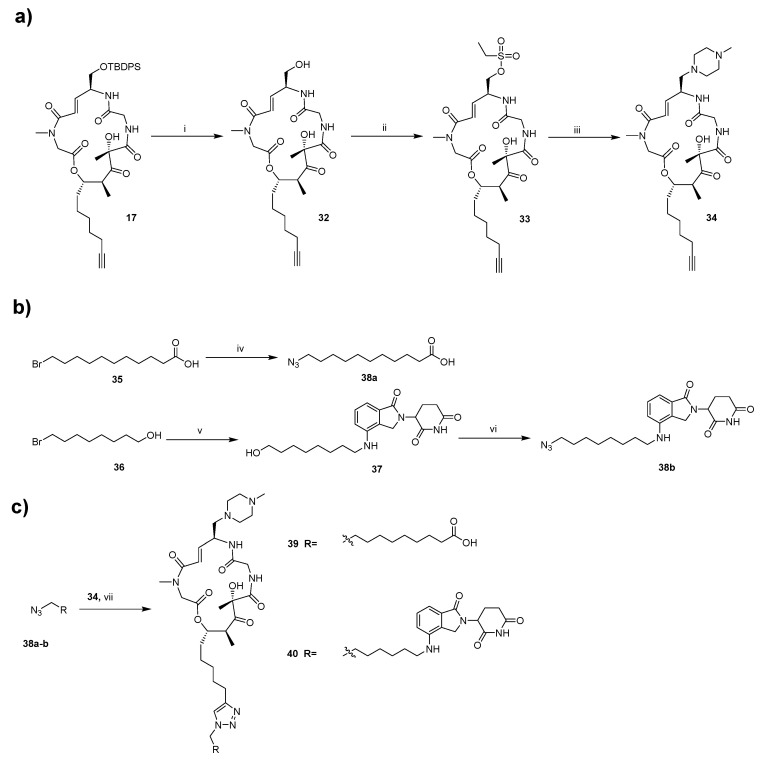
Synthesis of target compounds **39** and **40**^a^. (**a**) Preparation of the common alkyne-containing macrocyclic intermediate; (**b**) Synthesis of azide-containing tail fragments; (**c**) Construction of the target compounds via click reaction.

The synthetic approaches for the newly designed intermediate **17** and BE derivatives are outlined in Scheme 1, Scheme 2 and Scheme 3. Briefly, the synthesis of target compounds **29** and **31** is divided into three main stages. First, the intermediate **17** is synthesized (Scheme 1). Building upon our previous work on BE [30], we have the acid fragment **15** available, which is utilized as the starting material along with alcohol fragment **14** (its synthetic route is shown in Scheme 1). Initially, fragment **14** is coupled with fragment **15** in the presence of DIC and DMAP. Then, the protecting groups of *tert*-butyloxycarbonyl (Boc) and *tert*-butoxy (BuO) are removed using TFA. The resulting mixture undergoes a pivotal ring-closing reaction in the presence of HATU and DIPEA, allowing for the macrolactamization of **16** to form **17**. Subsequently, fragments bearing different polar tail groups are synthesized (Scheme 2a). These fragments are then assembled into the final products through a copper-catalyzed azide-alkyne cycloaddition (CuAAC) click reaction, followed by TBS deprotection and installation of the 1-methylpiperazine moiety (Scheme 2b).

*^a^* Reagents and conditions: (i) Trimethylsilylacetylene, n-BuLi, HMPA, THF, −78 °C, N_2_. (ii) *N*-methoxymethylamine hydrochloride, EDCI, HOBt, DCM, DIPEA, 10 °C, N_2_. (iii) DIBAL-H, −78 °C, DCM. (iv) BF_3_·Et_2_O, −78 °C, N_2_, DCM. (v) TBSOTf, 2,6-lutidine, DCM, 5 °C, N_2_. (vi) DIBAL-H, −78 °C, DCM, N_2_. (vii) *N*-Propionyl-(2R)-bornane-10,2-sultam, n-Bu_2_BOTf, DIPEA, 0 °C, DCM, N_2_. (viii) LiOH·H_2_O, 30% H_2_O_2_, THF/MeOH/H_2_O, 0 °C. (ix) Glycine tert-butyl ester, EDCI, HOBt, DCM, DIPEA, 0 °C, N_2_. (x) DMP, DCM, DIPEA, 0 °C, N_2_. (xi) KHMDS, D-CSO, −75 °C, N_2_, THF. (xii) TBAF, THF, 0 °C, N_2._ (xiii) DIC, DMP, DCM, N_2_, 0 °C, then TFA, DCM, N_2_, rt. (xiv) HATU, DIPEA, N_2_, THF, rt.

*^a^* Reagents and conditions: (i) 4-methylbenzeneboronic acid, Pd(dppf)_2_Cl_2_, K_2_CO_3_, 80 °C, DME. (ii) 1-bromobutane, Cs_2_CO_3_, DMF, rt. (iii) NBS, BPO, 80 °C, CCl_4_. (iv) TMSN_3_, TBAF, 55 °C, DMF. (v) Potassium phthalimide, K_2_CO_3_, 80 °C, DMF, then DIPEA, ethanesulfonyl chloride, rt. (vi) TMSN_3_, TBAF, 55 °C, DMF. (vii) Hydrazine monohydrate, THF, 60 °C, then 1,3-Di-Boc-2-methylisothiourea, DIPEA, DMF, 0 °C. (viii) Copper Sulfate Solution (1M), L-Ascorbic acid sodium salt (1M), H_2_O, *tert*-butanol, rt. (ix) 3HF·Et_3_N, THF, N_2_, rt. (x) Ethanesulfonyl chloride, Et_3_N, THF, rt. (xi) 1-methylpiperazine, THF, N_2_, rt. (xii) TFA, DCM, rt.

Similarly, the synthesis of target compounds **39** and **40** is divided into three main stages (Scheme 3). However, unlike the previous steps described in Scheme 1 and Scheme 2, we first remove the TBS protecting group from **17** using TBAF to obtain alcohol fragment **32**. This fragment is then coupled with 1-methylpiperazine to yield the common fragment, **34**, which is essential for the synthesis of compounds **39**–**40**. Subsequently, three different tail groups are synthesized. Finally, the corresponding final products are obtained via a click reaction. This modular approach enables the efficient preparation of diverse BE derivatives for structure-activity relationship (SAR) studies. All synthesized compounds are characterized in the Supporting Information.

*^a^* Reagents and conditions: (i) 3HF·Et_3_N, THF, N_2_, rt. (ii) Ethanesulfonyl chloride, Et_3_N, THF, rt. (iii) 1-methylpiperazine, THF, N_2_, rt. (iv) Azidotrimethylsilane, TBAF, 60 °C, DMF. (v) Lenalidomide, K_2_CO_3_, 100 °C, THF. (vi) DPPA, DBU, N_2_, 60 °C, THF. (vii) Copper Sulfate Solution (1M), L-Ascorbic acid sodium salt (1M), H_2_O, *tert*-butanol, rt.

The results demonstrated that the alkyne group at the tail of **17** can undergo click reactions with various azide-containing linear molecules, facilitating the synthesis of a diverse array of BE derivatives with tailored tails. Therefore, compound **17** serves as a crucial intermediate for the design and synthesis of macrocyclic drugs with antiproliferative activity against PANC-1 cells.

### 2.3. IC_50_, Lipophilicity and Solubility

The inhibitory activities against human pancreatic cancer PANC-1 cells of the synthesized compounds were evaluated by CCK-8 assay. Notably, aside from the BE and BE-NMP, which correspond to an IC_50_ of 0.72 ± 0.14 μM and 0.65 ± 0.19 μM, respectively, all the new compounds presented in this study were inactive against PANC-1 cells, with IC_50_ larger than 50 μM (Table 1), no matter whether hydrophilic or hydrophobic groups are introduced to the tail. To ensure that the observed loss of activity is not specific to the PANC-1 cell line, we further evaluated the original BE and two representative inactive derivatives that span the extremes of lipophilicity in the series (the bulky hydrophobic compound **29** and the highly polar compound **31**) in two additional human pancreatic cancer cell lines, PATU8988T and BxPC3. Consistent with the results in PANC-1, BE retained potent antiproliferative activity in both cell lines, whereas compounds **29** and **31** remained inactive (IC_50_ > 50 μM; see Figure S1 and Table S1 in the Supporting Information).

One explanation for the high IC_50_ values (i.e., low activity) of the new compounds is their potential poor solubility in aqueous solution or inadequate lipophilicity. To investigate this hypothesis, we determined kinetic solubility in potassium phosphate buffer at pH 7.4 (Table 1). Of the six BE derivatives tested, four exhibit high solubility (>200 μM at pH 7.4). The remaining two compounds, BE and **29**, show low solubilities (<0.1 μM). Hence, no direct relationship existed between the solubility and the IC_50_ of the compounds. Additionally, since approximately 95% of drugs are ionizable, assessment of lipophilicity at pH 7.4 (log *D*_7.4_) is essential during the initial phases of drug discovery [37]. In this study, we employ the shake-flask method to evaluate this property. Interestingly, BE and BE-NMP, which show activity against PANC-1 cells, had log *D*_7.4_ values greater than 5. Because lipophilicity directly reflects compound–membrane interaction, the activity of these compounds against PANC-1 cells likely stems from their interaction with the cell membrane.

**Table 1:** Antiproliferative activities of BE [31], BE-O [30], BE-NMP [31] and BE derivatives against PANC-1 cells, along with experimentally determined physicochemical properties.

Compound	IC_50_ ^[a]^	log *D*_7.4_ ^[b]^	Solubility ^[c]^	HBDs	PSA ^[d]^
BE	0.72 ± 0.14	>6.18 *	0.0047	3	142.1
BE-NMP	0.65 ± 0.19	5.05	209.70	3	148.5
BE-O	>50	ND ^[e]^	ND	3	169.8
**29**	>100	3.87	<0.063 *	3	188.5
**31**	>100	−1.06	267.66	7	241.2
**39**	>100	−0.30	290.67	4	216.6
**40**	>100	0.93	259.19	5	257.8
Progesterone	\	3.85	13.13	\	\

^[a]^ Data represent the mean (µM) from three independent experiments. ^[b]^ Distribution coefficients for 1-Octanol/PBS (pH = 7.4) are determined using the shake-flask method. Values marked with an asterisk (*) indicate that the sample signal in the aqueous phase is below the limit of detection; a peak area of 200 (representing the limit of detection) is used for the calculation. ^[c]^ Kinetic solubility in aqueous solution is measured in PBS (pH 7.4) and is expressed in µM. Values marked with an asterisk (*) indicate that no visible peak is detected. The solubility is estimated using a peak area value of 200 (representing the limit of detection), and the result is reported as less than the calculated value (e.g., <X µM). ^[d]^ Polar Surface Area (PSA) values (Å^2^) are obtained using ADMETLab 3.0 [38]. ^[e]^ Not determined.

### 2.4. Caco-2 Permeability Determination

One of the physicochemical quantities that mirrors the interaction of a compound with the cell membrane is permeability. Traditionally, measuring permeability with the PAMPA model provides estimates of passive permeability across a model membrane [39]. However, it lacks the critical biological components, such as efflux transporters, found in cell-based models like Caco-2 cells [40], particularly efflux transporters like P-glycoprotein (P-gp) abundantly expressed in Caco-2 cells, significantly influencing the permeability and bioavailability [41] of structurally complex compounds such as those investigated in this study. Therefore, we measure the cell permeability of the six compounds using Caco-2 cells, so that the apical to basolateral (A to B) and basolateral to apical (B to A) permeabilities, and thereby the efflux ratio, can be determined. Three commercial drugs (Table 2) are used as standard compounds to establish and validate our in-house assay.

As shown in Table 2, all BE derivatives exhibited significantly lower permeability than control compounds, even compared to atenolol, the low-permeability reference standard. This includes the active lead compounds BE and BE-NMP. However, their measured permeabilities may be artificially underestimated. For BE (and analog **29**), this underestimation likely stems from their extremely low solubility (BE: 0.0047 μM; **29**: <0.063 μM), which restricts the pool of dissolved molecules available for transport across the membrane. In the case of BE-NMP, high P-gp-mediated efflux (ER > 65.35) further confounds the results by diminishing net permeability [42,43,44] in the apical-to-basolateral (A-to-B) direction and masking its true intrinsic transmembrane permeability. Compounds **31** and **39**, despite bearing ionizable groups (log *D*_7.4_ < 0) (Table 1), showed slightly higher A-to-B values but remained impermeable, consistent with charged molecules facing high barriers in lipid bilayers [45,46,47]. Additionally, while polyarginine motifs [35,36] are known to confer cell permeability, a single arginine residue, such as the one in **31**, is generally insufficient to do so. These findings did not directly correlate with bioactivity, highlighting the challenges of Caco-2 assays for low-solubility, efflux-prone compounds—a common issue in early drug discovery [48,49].

Consequently, to address the limitations of experimental permeability measurements, computational free-energy calculations are employed to elucidate the relationship between bioactivity and transmembrane permeability. These calculations provide a direct measure of transmembrane permeability, unaffected by efflux transporters or solubility limitations, as shown below.

These results underscore the inherent limitations of Caco-2 assays in accurately assessing the intrinsic membrane permeability of highly lipophilic, poorly soluble, or efflux-prone compounds, such as those encountered in bRo5 chemical space. While the observed reductions in apparent permeability support the hypothesis that tail modifications impair passive transmembrane diffusion—a critical factor for accessing the intracellular target eEF1A1—a fundamental question remains: do these simplified derivatives retain binding affinity to the validated primary molecular target of BE, namely eEF1A1? To address this, we next evaluated direct target engagement using MST.

### 2.5. Target Engagement Studies

To investigate whether the loss of cellular activity in the BE derivatives results from diminished binding to the known intracellular target eEF1A1, we assessed direct target engagement using MST. Recombinant human eEF1A1 protein was titrated against the compounds, which were supplied in HEPES buffer (pH 7.4) and diluted as required. Binding interactions were quantified by monitoring changes in thermophoretic mobility. Detailed materials and protein labeling procedures are provided in the Supporting Information.

**Figure 2:**
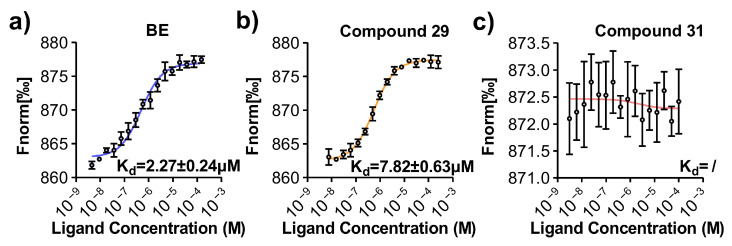
MST binding curves for (**a**) BE, (**b**) **29**, and (**c**) **31** to eEF1A1, with fitted *K*_d_ values. Each panel corresponds to a different compound as indicated, where colored lines represent MST traces obtained at varying concentrations of the respective compound.

BE served as the positive control in MST experiments, binding to recombinant human eEF1A1 with a dissociation constant (*K*_d_) of 2.27 ± 0.24 μM (Figure 2a), consistent with its known covalent target engagement [30] and potent antiproliferative activity. Among the inactive derivatives, we prioritized two representatives that span the extremes of lipophilicity modification in this series: compound **29** (bulky hydrophobic alkoxytriphenyl tail) and compound **31** (highly polar guanidinium-containing tail). Compound **29** exhibited modestly reduced affinity (*K*_d_ = 7.82 ± 0.63 μM; approximately 3.4-fold weaker than the parent BE; Figure 2b), whereas compound **31** showed no detectable binding (no saturation observed even at high concentrations; Figure 2c).

These results demonstrated that tail modifications can abolish cellular activity through at least two distinct mechanisms, depending on the nature of the substitution: (i) substantial or complete loss of binding affinity to eEF1A1, likely due to introduction of polar/charged functionalities incompatible with the binding pocket (exemplified by compound **31**, no detectable binding); and (ii) retention of measurable target affinity accompanied by loss of cellular potency (exemplified by compound **29**, *K*_d_ = 7.82 ± 0.63 μM, ~3.4-fold weaker than BE), consistent with impaired passive membrane permeation as a major contributing factor in this case. These findings highlight the dual challenge in optimizing bRo5 macrocycles: preserving both target recognition and cell entry.

### 2.6. Mechanism by Which Tail Modifications Affect Antiproliferative Activity of BE Derivatives

**Figure 3:**
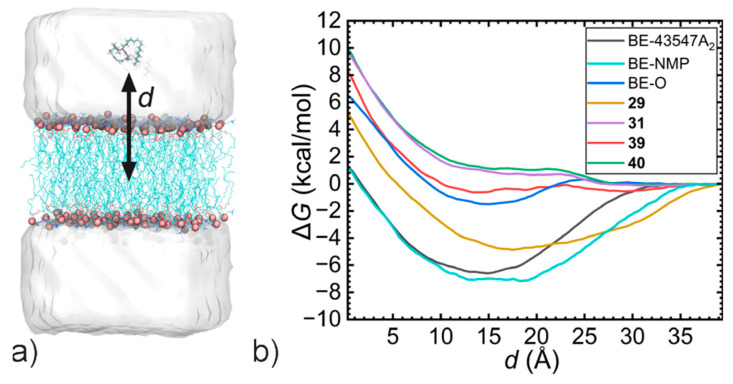
PMF curves characterizing the transmembrane processes of BE and its derivatives. (**a**) The reaction-coordinate model *d* represents the projection of the distance between the centers of mass of the BE-series and the membrane onto the *z*-axis. (**b**) Free-energy profiles.

To elucidate the molecular mechanism underlying the deactivation of BE derivatives, free-energy calculations characterizing BE and its derivatives were performed. The reaction-coordinate model is the projection of the distance between the center of mass of the BE-series and the membrane onto the *z*-axis. The convergence of the free-energy calculations is demonstrated in Figure S2 (simulation times for equilibration and free-energy calculations are provided in Table S2). As shown in Figure 3, active compounds BE and BE-NMP exhibited low transmembrane free-energy penalties (<2 kcal/mol), whereas the inactive derivatives tested displayed significantly higher values (5.6–10.3 kcal/mol). This correlation suggests that a high transmembrane energy barrier contributes to the loss of cellular activity for at least some derivatives.

To further investigate the mechanism behind the deactivation of BE derivatives, free-energy decomposition is performed. The original PMFs are broken down into compound-lipid Coulombic and van der Waals interactions, as well as solute-solvent interactions, which include both compound-water and lipid-water interactions. When the distance (*d*) approaches zero, the compound-lipid Coulombic interaction primarily involves unfavorable interactions between the hydrophilic groups and the hydrocarbon tails of lipids. In contrast, the compound-lipid van der Waals interaction describes favorable interactions between the hydrophobic groups and the hydrocarbon tails of lipids. Meanwhile, the solute-solvent interaction characterizes the dehydration contribution of the drug-like compound.

**Figure 4:**
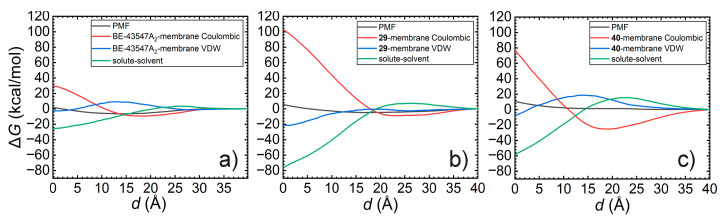
Free-energy decomposition for PMFs characterizing the transmembrane of (**a**) BE-43547A_2_. (**b**) **29**. and (**c**) **40**.

As shown in Figure 4, BE achieved a well-balanced interplay of interactions during the transmembrane process, where the compound-lipid van der Waals contribution remains near zero, and the favorable dehydration effects are offset by unfavorable interactions between the hydrophilic groups and the lipid hydrocarbon tails. For compound **29**, which retains measurable (albeit modestly reduced) binding affinity to eEF1A1, free-energy decomposition (Figure 4b) revealed that the introduction of a bulky alkoxytriphenyl tail, combined with the additional polar groups, substantially amplifies unfavorable interaction components. In this case, favorable dehydration and compound-lipid van der Waals contributions are insufficient to counterbalance the increased unfavorable Coulombic interactions with lipid tails, resulting in a disrupted energetic balance that likely impairs passive transmembrane diffusion.

Similar energetic trends were observed for compound **40** (Figure 4c). However, in the absence of direct target engagement data for compound **40** (and compound **39**), the extent to which impaired membrane permeability—versus potential loss of target binding—contributes to the observed deactivation remains hypothetical and requires future experimental validation.

Overall, these computational analyses indicate that, for derivatives where target binding is preserved (e.g., compound **29**), tail modifications can abolish cellular activity by disrupting the delicate balance of drug-membrane and drug-solvent interactions critical for low-energy transmembrane passage. For other derivatives, additional or alternative mechanisms—such as loss of target affinity—may play a significant role, highlighting the compound-specific nature of deactivation in this series.

## 3. Materials and Methods

### 3.1. Materials and Reagents

Details of materials and reagents used are provided in the Supporting Information.

### 3.2. Cell Culture

Human pancreatic cancer cell lines PANC-1, PATU8988T and BxPC3 were purchased from the Cancer Institute & Hospital of the Chinese Academy of Medical Sciences (Shanghai, China). All three cell lines were cultured in RPMI-DMEM medium containing 10% fetal bovine serum, cultured at a constant temperature of 37 °C in a humidified incubator with 5% CO_2_.

### 3.3. CCK-8 Assay

Pancreatic cancer cells are seeded into 96-well plates. After a proliferation time of 16 h, different concentrations of compounds are added into the corresponding wells and incubated for 72 h. 10 μL of CCK-8 solution is added into each well and incubated for 1–4 h at 37 °C. Shake the plates and read the OD value of each well at 450 nm on a microplate reader.

### 3.4. Caco-2 Cell Permeability Study

For Caco-2 cells (originally obtained from the American Type Culture Collection; ATCC, Manassas, VA, USA), 50 μL and 25 mL of cell culture medium are added to each well of the Transwell insert and reservoir, respectively. And then the HTS transwell plates are incubated at 37 °C, 5% CO_2_ for 1 h before cell seeding. Caco-2 cells are diluted to 6.86 × 10^5^ cells/mL with culture medium and 50 μL of cell suspension is dispensed into the filter well of the 96-well HTS Transwell plate. Cells are cultivated for 14–18 days in a cell culture incubator at 37 °C, 5% CO_2_, 95% relative humidity. Cell culture medium is replaced every other day, beginning no later than 24 h after initial plating. Stock solutions of the test compounds are prepared at a concentration of 10 mM in DMSO. Similarly, stock solutions of the positive control compounds, digoxin, minoxidil and atenolol are also prepared in DMSO at a concentration of 10 mM and utilized as controls in this assay. Detailed assay procedures are provided in the SI. Before the experiment is conducted, transepithelial electrical resistance (TEER) is measured for each insert, using the Millicell Epithelial Volt-Ohm measuring system (Millipore, Burlington, MA, USA), and TEER is always above 400 Ω·cm^2^ (Table S3).

### 3.5. Solubility Measurements

Solubility is determined in 100 mM sodium phosphate buffer (PBS), pH 7.4. The stock solutions of test compound and control compound progesterone are prepared in DMSO at a concentration of 10 mM. 30 µL of working solutions of each sample is placed in order into their proper 96-well rack. 970 µL of PBS pH 7.4 is added into each vial of the cap-less Solubility Sample plate. The assay is performed in duplicate. Add one stir stick to each vial and seal using a molded PTFE/Silicone plug. Then the Solubility Sample plate is transferred to the Eppendorf Thermomixer Comfort plate shaker and shaken at 25 °C at 1100 RPM for 2 h. After completion of the 2 h, plugs are removed and the stir sticks are removed using a big magnet, the samples from the Solubility Sample plate are transferred into the filter plate. Using the Vacuum Manifold, all the samples are filtered. Aliquot of 5 µL is taken from the filtrate, followed by addition of 5 µL DMSO and 490 µL of a mixture of H_2_O and acetonitrile (1:1 in *v*/*v*). 200 μL of diluent is transferred to a new 96-well plate for LC-MS/MS analysis (see SI Bioanalytical Method for Solubility Measurement). The dilution factor is changed according to the solubility values and the LC-MS signal response.

### 3.6. Log D_7.4_ Measurements

To determine the log *D* values of test compounds, a 100 mM PBS at pH 7.4 is first prepared by dissolving 14.2 g/L Na_2_HPO_4_ and 12.0 g/L NaH_2_PO_4_ in deionized water to create basic and acidic solutions, respectively, which are stored at 4 °C for up to 30 day. The basic solution is titrated with the acidic solution to achieve pH 7.4, with the pH verified and adjusted to 7.4 ± 0.1 on the experiment day. Saturated 1-octanol and PBS (pH 7.4) are also prepared. The stock solutions of test compounds and control compound progesterone are prepared in DMSO at a concentration of 10 mM (0.666 mM: compound **39**). The detailed procedure for log *D* determinations can be found in the SI.

### 3.7. MST Assay

Compounds were serially diluted (16-point, 2-fold) in assay buffer (50 mM HEPES, pH 7.4, 0.05% Tween-20) as ligands. Labeled eEF1A1 protein (target) was mixed 1:1 with each dilution and incubated at room temperature for 20 min. Samples were loaded into premium capillaries and analyzed on the Monolith X instrument following the manufacturer’s protocol, with infrared laser excitation at 1480 nm to generate a temperature gradient. Thermophoretic mobility was monitored via fluorescence changes before, during, and after laser activation.

Data were analyzed using NanoTemper Analysis software (version 2.3) to fit binding curves and determine dissociation constants (*K*_d_). Experiments were performed in triplicate, and *K*_d_ values are reported as mean ± standard deviation.

### 3.8. Molecular Modeling

Molecular dynamics (MD) simulations were adopted to investigate the interaction of BE derivatives and the cell membrane. A 2 × 64 pure POPC bilayer is generated using the CHARMM-GUI and placed in a box measuring 66 Å × 66 Å × 120 Å. A 40 Å thick water slab is positioned on top of each leaflet [50]. The BE-series molecules are positioned 40 Å away from the center of mass of the bilayer. 0.15 M KCl is added to the water box. The topology and parameters for the BE-series compounds are generated using the Ligand Reader & Modeler module of CHARMM-GUI [51], employing the CHARMM Generalized Force Field (CGenFF) [52]. The CHARMM36 and TIP3P force fields are used for modeling lipids and water, respectively [53].

### 3.9. Classical Molecular Dynamics Simulation

All MD simulations reported in this study are conducted using NAMD 3.0 [54]. A Langevin thermostat with a damping coefficient of 1 ps^−1^ is employed to maintain the temperature at 300 K. The Langevin piston method [55] is used to maintain a pressure of 1 atm. Bonds involving hydrogen atoms are constrained using the RATTLE algorithm [56]. The SHAKE algorithm is applied to constrain water molecules to their equilibrium geometry [57]. Long-range electrostatic forces are evaluated using the particle–mesh Ewald algorithm with a grid spacing of 1.2 Å, while a smoothed 9-Å spherical cutoff is applied to truncate short-range van der Waals and electrostatic interactions. The r-RESPA multiple time-stepping algorithm integrates the equations of motion with an effective time step of 2 fs for short-range interactions and 4 fs for long-range interactions.

### 3.10. Free-Energy Calculations

The well-tempered metadynamics-extended adaptive biasing force (WTM-eABF) method [58,59,60] implemented within the Colvars module [61] is used to calculate the free-energy profile, or potential of mean force (PMF), describing the transmembrane of drug-like molecules. The reaction-coordinate model, *w*(*z*), is defined as the projection of the distance between the centers of mass (COMs) of drug-like molecules and the membrane onto the *z* axis. The corrected *z*-averaged restraint (CZAR) estimator [62] is adopted to numerically estimate the free-energy profile.

## 4. Conclusions

The goal of this study was to evaluate the effects of side-chain modifications on the physicochemical properties of a series of BE-based compounds sharing a common core scaffold. In particular, we focused on characterizing BE analogs with varying side-chain polarity, which spanned a wide range of log *D* values. To this end, we prepared these analogs and evaluated their key physicochemical properties to assess the impact of each structural change.

We developed a concise synthetic approach leading to intermediate **17**, which enables click-based tail variation for BE macrocycles—overcoming synthetic challenges in PCSC-targeted SAR studies. These peptides feature a macrocycle with antiproliferative activity against PANC-1 cells and a tail that can be either hydrophobic or hydrophilic. For instance, BE derivatives **29**, **31**, **39**, and **40** have been designed and synthesized successfully. Despite these advances, our understanding of the absorption, distribution, metabolism, excretion, and toxicity (ADMET) profiles of macrocycles remains limited [32] which hampers drug discovery. Thus, there is a pressing need for better insights into the structural features that control cell permeability, including passive diffusion and transporter-mediated uptake [63]. To address this, we evaluated the antiproliferative activity against multiple human pancreatic cancer cell lines (including PANC-1, PaTu8988T, and BxPC3), along with kinetic solubility, lipophilicity, and apparent permeability across Caco-2 monolayers for these BE macrocycle derivatives. Our results showed that substituting the lipid tail with various side chains (derived from non-natural amino acids) in the cell-permeable cyclic peptide BE exerts a major impact on its biological activity. This effect correlates with changes in the lipophilicity of the side chain. These findings indicate that reducing lipophilicity to increase solubility comes at the expense of biological activity. Moreover, tail modifications with hydrogen-bond donors impaired membrane permeability, and polar surface area values above established thresholds further exacerbated this issue.

Importantly, direct target engagement studies via MST revealed that loss of antiproliferative activity arises through compound-specific mechanisms: in some derivatives (e.g., **31**), introduction of highly polar/charged side chains abolishes detectable binding to eEF1A1, likely due to incompatibility with the target pocket; in others (e.g., **29**), binding affinity is largely preserved but cellular potency is nevertheless lost. For these latter derivatives, microsecond-scale molecular dynamics simulations and free-energy calculations indicated markedly increased transmembrane free-energy penalties (>5 kcal/mol versus < 2 kcal/mol for active compounds), resulting from disruption of the delicate balance between drug-membrane and drug-solvent interactions that enables efficient passive permeation in the parent natural product BE. For compounds **39** and **40**, where target engagement data are currently unavailable, computational analyses suggest similar permeability impairments, but contributions from potential loss of target binding cannot be excluded and require future experimental confirmation. These compounds illustrate how minor structural modifications, as observed in comparisons such as BE-NMP versus compound **29**, can result in substantial variations in cell permeability for macrocyclic compounds. Based on these results, we suggested that hydrophobic tags, such as alkyl chains (present in the tail groups of BE and BE-NMP), promote efficient and stable membrane anchoring due to their similarity to membrane lipids [64,65]. In contrast, introducing charged or bulky groups into the tail structures of our BE analogs eliminates this anchoring ability.

In recent years, computer-aided drug design (CADD) has gained significant popularity, with methods for high-throughput [66,67,68] or accurate [69,70,71,72] prediction of protein-ligand binding affinities being widely adopted. However, the transmembrane properties of drug candidates are often overlooked during the CADD process. Experimental determination of membrane permeability faces limitations when screening large libraries of molecules and when evaluating new, unsynthesized compounds, particularly complex natural products that are challenging to synthesize. The lack of antiproliferative activity observed for the four newly synthesized derivatives in this study does not represent a setback but rather a profound advancement in elucidating the structure-activity relationship of the parent cyclic depsipeptide BE. In this study, we show that PMF calculations and free-energy decomposition, based on the WTM-eABF algorithm, have significant potential not only for predicting the transmembrane properties of drug candidates but also for elucidating the underlying mechanisms to facilitate rational drug design. We underscore the essential role of “rational failure” in drug discovery: far from a mere endpoint, it acts as a pivotal catalyst, thereby informing the strategic prioritization of branched alkyl chains in next-generation scaffolds to recapture potency while reducing hemolytic risks. We believe the computational workflow presented in this study can be broadly applied in future drug development efforts. Overall, we recommend a cautious approach when making modifications to active hits. Our study established a baseline data set to enable the design and synthesis of macrocyclic peptides and the knowledge gained will be used in the development of novel peptide therapeutics to come.

## Figures and Tables

**Table 2:** Caco-2 apparent permeability and efflux ratio (ER) of six BE derivatives with control compounds ^[a]^.

Mean *P*_app_ (10^−6^ cm/s)			Mean % Solution Recovery		
Compound	A to B	B to A	Efflux Ratio	A to B	B to A
BE	<0.00224	<0.000745	NC ^[b]^	<95.54	<65.27
BE-NMP	<0.0144	0.942	>65.35	<23.48	8.99
**29**	<0.00798	<0.00266	<NC	<92.87	<71.11
**31**	0.0348	0.0894	2.57	108.94	70.43
**39**	0.0836	0.105	1.26	99.46	48.60
**40**	<0.0116	0.13	>10.82	<89.39	50.41
Atenolol	0.52	0.67	1.29	110.26	100.96
Digoxin	0.77	19.98	25.86	83.19	95.03
Minoxidil	9.52	10.32	1.08	103.76	105.57

^[a]^ All assays are performed at a concentration of 5 μM. *P*_app_: apparent permeability rate coefficient. Efflux ratio: *P*_app (B to A)_/*P*_app (A to B)_. Atenolol, digoxin, and minoxidil are used as the low permeability control, high efflux control, and high permeability control, respectively. *P*_app_ values for BE, **29**, and BE-NMP may be underestimated due to low solubility and/or high efflux. ^[b]^ Not calculated.

## Data Availability

The original data presented in the study are included in the article/Supplementary Materials; further inquiries can be directed to the corresponding author.

## Supplementary Materials

The following supporting information can be downloaded at: https://www.mdpi.com/article/10.3390/md24010013/s1, Figure S1: Characterization of biological activity; Table S1: The effects of compounds against two pancreatic cancer cell lines were evaluated using BE as the benchmark; Scheme S1: Synthesis of intermediate **17**; Figure S2: Convergence of the PMF calculated from WTM-eABF simulation; Table S2: Simulation times of the equilibration and free-energy calculations; Scheme S2: Synthesis of target compounds **29** and **31**; Scheme S3: Synthesis of target compounds **39** and **40**; Table S3: The assessment of caco-2 cell monolayer integrity [30,69].

## Abbreviations

The following abbreviations are used in this manuscript:

DMP	Dess-Martin periodinane
DIC	*N*,*N*-diisopropylcarbodiimide
DIPEA	*N*,*N*-Diisopropylethylamine
DMSO	dimethyl sulfoxide
DBU	1,8-diazabicyclo[5.4.0]undec-7-ene
DIBAL-H	diisobutylaluminum hydride
DPPA	diphenylphosphoryl azide
CSO	camphorsulfonyloxaziridine
DME	dimethyl ether
DMF	dimethylformamide
DMAP	4-dimethylaminopyridine
EDCI	1-ethyl-3(3-dimethylpropylamine) carbodiimide
EA	ethyl acetate
HATU	2-(7-Azabenzotriazol-1-yl)-*N*,*N*,*N*′,*N*′-tetramethyluronium hexafluorophosphate
HOBT	1-hydroxybenzotriazole
HMPA	hexamethylphosphoramide
KHMDS	potassium bis(trimethylsilyl)amide
PE	petroleum ether
TBAF	tetrabutylammonium fluoride
TBSOTf	*tert*-butyldimethylsilyl triflate
TFA	trifluoroacetic acid
THF	tetrahydrofuran
TBS	*tert*-butyldimethylsilyl
TMSN_3_	trimethylsilyl azide

## References

[1] Bray F., Laversanne M., Sung H., Ferlay J., Siegel R.L., Soerjomataram I., Jemal A. (2024). Global cancer statistics 2022: GLOBOCAN estimates of incidence and mortality worldwide for 36 cancers in 185 countries. CA Cancer J. Clin..

[2] Babiker H.M., Karass M., Recio-Boiles A., Chandana S.R., McBride A., Mahadevan D. (2019). Everolimus for the treatment of advanced pancreatic ductal adenocarcinoma (PDAC). Expert Opin. Investig. Drug.

[3] Kieler M., Unseld M., Bianconi D., Scheithauer W., Prager G.W. (2019). A real-world analysis of second-line treatment options in pancreatic cancer: Liposomal-irinotecan plus 5-fluorouracil and folinic acid. Ther. Adv. Med. Oncol..

[4] Luchini C., Pea A., Yu J., He J., Salvia R., Riva G., Weiss M.J., Bassi C., Cameron J.L., Hruban R.H. (2019). Pancreatic cancer arising in the remnant pancreas is not always a relapse of the preceding primary. Mod. Pathol..

[5] Tao J., Yang G., Zhou W., Qiu J., Chen G., Luo W., Zhao F., You L., Zheng L., Zhang T. (2021). Targeting hypoxic tumor microenvironment in pancreatic cancer. J. Hematol. Oncol..

[6] Izuishi K., Kato K., Ogura T., Kinoshita T., Esumi H. (2000). Remarkable tolerance of tumor cells to nutrient deprivation: Possible new biochemical target for cancer therapy. Cancer Res..

[7] Visvader J.E., Lindeman G.J. (2012). Cancer stem cells: Current status and evolving complexities. Cell Stem Cell.

[8] Dougherty P.G., Sahni A., Pei D. (2019). Understanding Cell Penetration of Cyclic Peptides. Chem. Rev..

[9] Ji X., Nielsen A.L., Heinis C. (2024). Cyclic Peptides for Drug Development. Angew. Chem. Int. Ed. Engl..

[10] Brudy C., Walz C., Spiske M., Dreizler J.K., Hausch F. (2024). The Missing Link(er): A Roadmap to Macrocyclization in Drug Discovery. J. Med. Chem..

[11] Li W., Schlecker A., Ma D. (2010). Total synthesis of antimicrobial and antitumor cyclic depsipeptides. Chem. Comm..

[12] Li T., Jiang S., Li T., Xu H., Zhang X., Yan R., Wu X., Jin Y., Wang Z. (2024). Exploring the potential of cyclic peptidyl antitumor agents derived from natural macrocyclic peptide phakellistatin 13. J. Med. Chem..

[13] Buyanova M., Pei D. (2022). Targeting intracellular protein–protein interactions with macrocyclic peptides. Trends Pharmacol. Sci..

[14] Nielsen D.S., Shepherd N.E., Xu W., Lucke A.J., Stoermer M.J., Fairlie D.P. (2017). Orally absorbed cyclic peptides. Chem. Rev..

[15] Witek J., Keller B.G., Blatter M., Meissner A., Wagner T., Riniker S. (2016). Kinetic models of cyclosporin A in polar and apolar environments reveal multiple congruent conformational states. J. Chem. Inf. Model..

[16] Liu J., Farmer J.D., Lane W.S., Friedman J., Weissman I., Schreiber S.L. (1991). Calcineurin is a common target of cyclophilin-cyclosporin A and FKBP-FK506 complexes. Cell.

[17] Dunn C.J., Wagstaff A.J., Perry C.M., Plosker G.L., Goa K.L. (2001). Cyclosporin: An Updated Review of the Pharmacokinetic Properties, Clinical Efficacy and Tolerability of a Microemulsion-Based Formulation (Neoral®) su1 in Organ Transplantation. Drugs.

[18] Rezai T., Yu B., Millhauser G.L., Jacobson M.P., Lokey R.S. (2006). Testing the conformational hypothesis of passive membrane permeability using synthetic cyclic peptide diastereomers. J. Am. Chem. Soc..

[19] Rezai T., Bock J.E., Zhou M.V., Kalyanaraman C., Lokey R.S., Jacobson M.P. (2006). Conformational flexibility, internal hydrogen bonding, and passive membrane permeability: Successful in silico prediction of the relative permeabilities of cyclic peptides. J. Am. Chem. Soc..

[20] Rand A.C., Leung S.S., Eng H., Rotter C.J., Sharma R., Kalgutkar A.S., Zhang Y., Varma M.V., Farley K.A., Khunte B. (2012). Optimizing PK properties of cyclic peptides: The effect of side chain substitutions on permeability and clearance. MedChemComm.

[21] Qian Z., Liu T., Liu Y.-Y., Briesewitz R., Barrios A.M., Jhiang S.M., Pei D. (2013). Efficient delivery of cyclic peptides into mammalian cells with short sequence motifs. ACS Chem. Biol..

[22] Hess S., Ovadia O., Shalev D.E., Senderovich H., Qadri B., Yehezkel T., Salitra Y., Sheynis T., Jelinek R., Gilon C. (2007). Effect of structural and conformation modifications, including backbone cyclization, of hydrophilic hexapeptides on their intestinal permeability and enzymatic stability. J. Med. Chem..

[23] Beck J.G., Chatterjee J., Laufer B., Kiran M.U., Frank A.O., Neubauer S., Ovadia O., Greenberg S., Gilon C., Hoffman A. (2012). Intestinal permeability of cyclic peptides: Common key backbone motifs identified. J. Am. Chem. Soc..

[24] Naylor M.R., Ly A.M., Handford M.J., Ramos D.P., Pye C.R., Furukawa A., Klein V.G., Noland R.P., Edmondson Q., Turmon A.C. (2018). Lipophilic permeability efficiency reconciles the opposing roles of lipophilicity in membrane permeability and aqueous solubility. J. Med. Chem..

[25] Wang S., Konig G., Roth H.-J., Fouché M., Rodde S., Riniker S. (2021). Effect of flexibility, lipophilicity, and the location of polar residues on the passive membrane permeability of a series of cyclic decapeptides. J. Med. Chem..

[26] Yang Y., Engkvist O., Llinàs A., Chen H. (2012). Beyond size, ionization state, and lipophilicity: Influence of molecular topology on absorption, distribution, metabolism, excretion, and toxicity for druglike compounds. J. Med. Chem..

[27] Nishioka H., Nakajima S., Nagashima M., Kojiri K., Suda H. (1998). BE-43547 Series Substances, Their Manufacture with *Streptomyces* Species, and Their Use as Antitumor Agent.

[28] Kranthikumar R. (2021). Toward the synthesis of the hypoxia selective anticancer agent BE-43547 A 2. Org. Biomol. Chem..

[29] Sun Y., Ding Y., Li D., Zhou R., Su X., Yang J., Guo X., Chong C., Wang J., Zhang W. (2017). Cyclic Depsipeptide BE-43547A(2): Synthesis and Activity against Pancreatic Cancer Stem Cells. Angew. Chem. Int. Ed. Engl..

[30] Liu C., Wang L., Sun Y., Zhao X., Chen T., Su X., Guo H., Wang Q., Xi X., Ding Y. (2022). Probe Synthesis Reveals Eukaryotic Translation Elongation Factor 1 Alpha 1 as the Anti-Pancreatic Cancer Target of BE-43547A(2). Angew. Chem. Int. Ed. Engl..

[31] Guo J.-S., Li J.-J., Wang Z.-H., Liu Y., Yue Y.-X., Li H.-B., Zhao X.-H., Sun Y.-J., Ding Y.-H., Ding F. (2023). Dual hypoxia-responsive supramolecular complex for cancer target therapy. Nat. Commun..

[32] Giordanetto F., Kihlberg J. (2014). Macrocyclic drugs and clinical candidates: What can medicinal chemists learn from their properties?. J. Med. Chem..

[33] Hein J.E., Fokin V.V. (2010). Copper-catalyzed azide–alkyne cycloaddition (CuAAC) and beyond: New reactivity of copper (I) acetylides. Chem. Soc. Rev..

[34] Szymanski M., Chmielewska S., Czyzewska U., Malinowska M., Tylicki A. (2022). Echinocandins—Structure, mechanism of action and use in antifungal therapy. J. Enzyme Inhib. Med. Chem..

[35] McKinlay C.J., Waymouth R.M., Wender P.A. (2016). Cell-Penetrating, Guanidinium-Rich Oligophosphoesters: Effective and Versatile Molecular Transporters for Drug and Probe Delivery. J. Am. Chem. Soc..

[36] Wender P.A., Mitchell D.J., Pattabiraman K., Pelkey E.T., Steinman L., Rothbard J.B. (2000). The design, synthesis, and evaluation of molecules that enable or enhance cellular uptake: Peptoid molecular transporters. Proc. Natl. Acad. Sci. USA.

[37] Duan Y.J., Fu L., Zhang X.C., Long T.Z., He Y.H., Liu Z.Q., Lu A.P., Deng Y.F., Hsieh C.Y., Hou T.J. (2023). Improved GNNs for Log D(7.4) Prediction by Transferring Knowledge from Low-Fidelity Data. J. Chem. Inf. Model..

[38] Fu L., Shi S., Yi J., Wang N., He Y., Wu Z., Peng J., Deng Y., Wang W., Wu C. (2024). ADMETlab 3.0: An updated comprehensive online ADMET prediction platform enhanced with broader coverage, improved performance, API functionality and decision support. Nucleic Acids Res..

[39] Kansy M., Avdeef A., Fischer H. (2004). Advances in screening for membrane permeability: High-resolution PAMPA for medicinal chemists. Drug Discov. Today Technol..

[40] Hubatsch I., Ragnarsson E.G., Artursson P. (2007). Determination of drug permeability and prediction of drug absorption in Caco-2 monolayers. Nat. Protoc..

[41] Sugano K., Kansy M., Artursson P., Avdeef A., Bendels S., Di L., Ecker G.F., Faller B., Fischer H., Gerebtzoff G. (2010). Coexistence of passive and carrier-mediated processes in drug transport. Nat. Rev. Drug Discov..

[42] Lin J.H., Yamazaki M. (2003). Clinical relevance of P-glycoprotein in drug therapy. Drug Metab. Rev..

[43] Batista da Silva Junior J., Marinho Dezani T., Bersani Dezani A., Helena dos Reis Serra C. (2015). Evaluating potential P-gp substrates: Main aspects to choose the adequate permeability model for assessing gastrointestinal drug absorption. Mini Rev. Med. Chem..

[44] Saaby L., Brodin B. (2017). A critical view on in vitro analysis of P-glycoprotein (P-gp) transport kinetics. J. Pharm. Sci..

[45] Shinoda W. (2016). Permeability across lipid membranes. Biochim. Biophys. Acta (BBA)-Biomembr.

[46] Vorobyov I., Olson T.E., Kim J.H., Koeppe R.E., Andersen O.S., Allen T.W. (2014). Ion-induced defect permeation of lipid membranes. Biophys. J..

[47] Patel S.J., Van Lehn R.C. (2018). Characterizing the molecular mechanisms for flipping charged peptide flanking loops across a lipid bilayer. J. Phys. Chem. B.

[48] Koziolek M., Augustijns P., Berger C., Cristofoletti R., Dahlgren D., Keemink J., Matsson P., McCartney F., Metzger M., Mezler M. (2023). Challenges in permeability assessment for oral drug product development. Pharmaceutics.

[49] Liu T., Chang L.-J., Uss A., Chu I., Morrison R.A., Wang L., Prelusky D., Cheng K.-C., Li C. (2010). The impact of protein on Caco-2 permeability of low mass balance compounds for absorption projection and efflux substrate identification. J. Pharm. Biomed. Anal..

[50] Wu E.L., Cheng X., Jo S., Rui H., Song K.C., Dávila-Contreras E.M., Qi Y., Lee J., Monje-Galvan V., Venable R.M. (2014). CHARMM-GUI membrane builder toward realistic biological membrane simulations. J. Comput. Chem..

[51] Kim S., Lee J., Jo S., Brooks C.L., Lee H.S., Im W. (2017). CHARMM-GUI ligand reader and modeler for CHARMM force field generation of small molecules. J. Comput. Chem..

[52] Vanommeslaeghe K., Hatcher E., Acharya C., Kundu S., Zhong S., Shim J., Darian E., Guvench O., Lopes P., Vorobyov I. (2010). CHARMM general force field: A force field for drug-like molecules compatible with the CHARMM all-atom additive biological force fields. J. Comput. Chem..

[53] Klauda J.B., Venable R.M., Freites J.A., O’Connor J.W., Tobias D.J., Mondragon-Ramirez C., Vorobyov I., MacKerell A.D., Pastor R.W. (2010). Update of the CHARMM all-atom additive force field for lipids: Validation on six lipid types. J. Phys. Chem. B.

[54] Phillips J.C., Braun R., Wang W., Gumbart J., Tajkhorshid E., Villa E., Chipot C., Skeel R.D., Kale L., Schulten K. (2005). Scalable molecular dynamics with NAMD. J. Comput. Chem..

[55] Feller S.E., Zhang Y., Pastor R.W., Brooks B.R. (1995). Constant pressure molecular dynamics simulation: The Langevin piston method. J. Comput. Phys..

[56] Andersen H.C. (1983). Rattle: A “velocity” version of the shake algorithm for molecular dynamics calculations. J. Comput. Phys..

[57] Miyamoto S., Kollman P.A. (1992). Settle: An analytical version of the SHAKE and RATTLE algorithm for rigid water models. J. Comput. Chem..

[58] Fu H., Chen H., Wang X.A., Chai H., Shao X., Cai W., Chipot C. (2020). Finding an optimal pathway on a multidimensional free-energy landscape. J. Chem. Inf. Model..

[59] Fu H., Shao X., Cai W., Chipot C. (2019). Taming Rugged Free Energy Landscapes Using an Average Force. Acc. Chem. Res..

[60] Fu H., Zhang H., Chen H., Shao X., Chipot C., Cai W. (2018). Zooming across the free-energy landscape: Shaving barriers, and flooding valleys. J. Phys. Chem. Lett..

[61] Fiorin G., Klein M.L., Hénin J. (2013). Using collective variables to drive molecular dynamics simulations. Mol. Phys..

[62] Lesage A., Lelièvre T., Stoltz G., Hénin J. (2017). Smoothed biasing forces yield unbiased free energies with the extended-system adaptive biasing force method. J. Phys. Chem. B.

[63] Kotz J. (2012). Bringing macrocycles full circle. Sci.-Bus. Exch..

[64] Ma Y.-H., Zhu Y., Wu H., He Y., Zhang Q., Huang Q., Wang Z., Xing H., Qiu L., Tan W. (2024). Domain-targeted membrane partitioning of specific proteins with DNA nanodevices. J. Am. Chem. Soc..

[65] Liu H., Kwong B., Irvine D.J. (2011). Membrane anchored immunostimulatory oligonucleotides for in vivo cell modification and localized immunotherapy. Angew. Chem. Int. Ed..

[66] Wang E., Sun H., Wang J., Wang Z., Liu H., Zhang J.Z., Hou T. (2019). End-point binding free energy calculation with MM/PBSA and MM/GBSA: Strategies and applications in drug design. Chem. Rev..

[67] Gentile F., Oprea T., Tropsha A., Cherkasov A. (2023). Surely you are joking, Mr Docking!. Chem. Soc. Rev..

[68] Muhammed M.T., Aki-Yalcin E. (2024). Molecular docking: Principles, advances, and its applications in drug discovery. Lett. Drug Des. Discov..

[69] Fu H., Chen H., Blazhynska M., Goulard Coderc de Lacam E., Szczepaniak F., Pavlova A., Shao X., Gumbart J.C., Dehez F., Roux B. (2022). Accurate determination of protein: Ligand standard binding free energies from molecular dynamics simulations. Nat. Protoc..

[70] Liu H., Fu H., Chipot C., Shao X., Cai W. (2021). Accuracy of alternate nonpolarizable force fields for the determination of protein–ligand binding affinities dominated by cation−π interactions. J. Chem. Theory Comput..

[71] Fu H., Zhou Y., Jing X., Shao X., Cai W. (2022). Meta-analysis reveals that absolute binding free-energy calculations approach chemical accuracy. J. Med. Chem..

[72] Fu H., Chipot C., Shao X., Cai W. (2023). Standard Binding Free-Energy Calculations: How Far Are We from Automation?. J. Phys. Chem. B.

