# Supplementary material for: Fine-Tuning Side Chain Substitutions: Impacts on the Lipophilicity–Solubility–Permeability Interplay in Macrocyclic Peptides
Source: Mar Drugs. 2025 Dec 25;24(1):13. doi: 10.3390/md24010013 (PMC12843137; doi:10.3390/md24010013)
Supplement: Supplementary file 1 [file marinedrugs-24-00013-s001.zip › marinedrugs-4013512-supplementary.pdf]

Supplementary Information

Fine-Tuning Side Chain Substitutions: Impacts on the Lipophilicity–Solubility–Permeability Interplay in Macrocyclic Peptides

Yangping Deng [1], Hengwei Bian [1, 2, 3], Hongbo Li [4], Yingjun Cui [4], Sizheng Li [1], Jing Li [1], Li Chen [1, 2], Xuemei Zhang [4], Zhuo Shen [4], Fengyue Li [4], Yue Chen [1, 2,*] and Haohao Fu [1, 2, 3,*]

[1] State Key Laboratory of Medicinal Chemical Biology, Frontiers Science Center for New Organic Matter, College of Chemistry, Nankai University, 94 Weijin Road, Tianjin, 300071, China

[2] Haihe Laboratory of Sustainable Chemical Transformations, 6 Keyanxi Road, Tianjin, 300192, China

[3] Research Center for Analytical Sciences, Tianjin Key Laboratory of Biosensing and Molecular Recognition, College of Chemistry, Nankai University, Tianjin 300071, China

[4] Accendatech Company, Ltd., Tianjin 300193, China

1. Characterization of biological activity

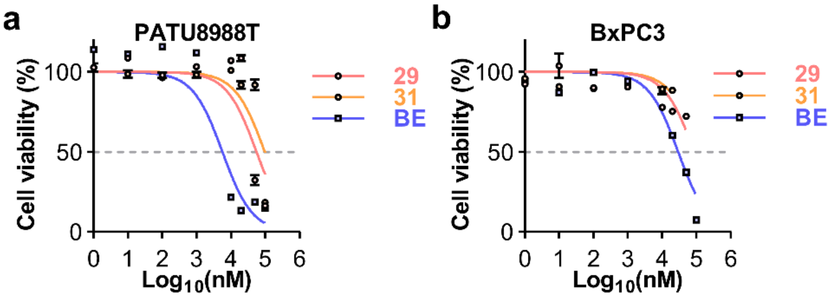

Supplementary Figure S1. Cell viability is assessed using the CCK-8 assay following 72 h treatment with the tested compounds in pancreatic cancer cell lines: a) PATU8988T, b) BxPC3.

Supplementary Table S1. The effects of compounds against two pancreatic cancer cell lines were evaluated using BE[30] as the benchmark.

	Cell proliferation IC_{50}(μM)[a]	
**Compound**	**PATU8988T**	**BxPC3**
**29**	>50	>50
**31**	>50	>100
BE	5.7 ± 0.98	29.5 ± 3.18

[a] IC_{50} values of the tested compounds are determined in PATU8988T and BxPC3 cells. Data are shown as mean ± standard error of the mean (SEM) (n = 3)

2. Convergence of the free-energy calculations [69]

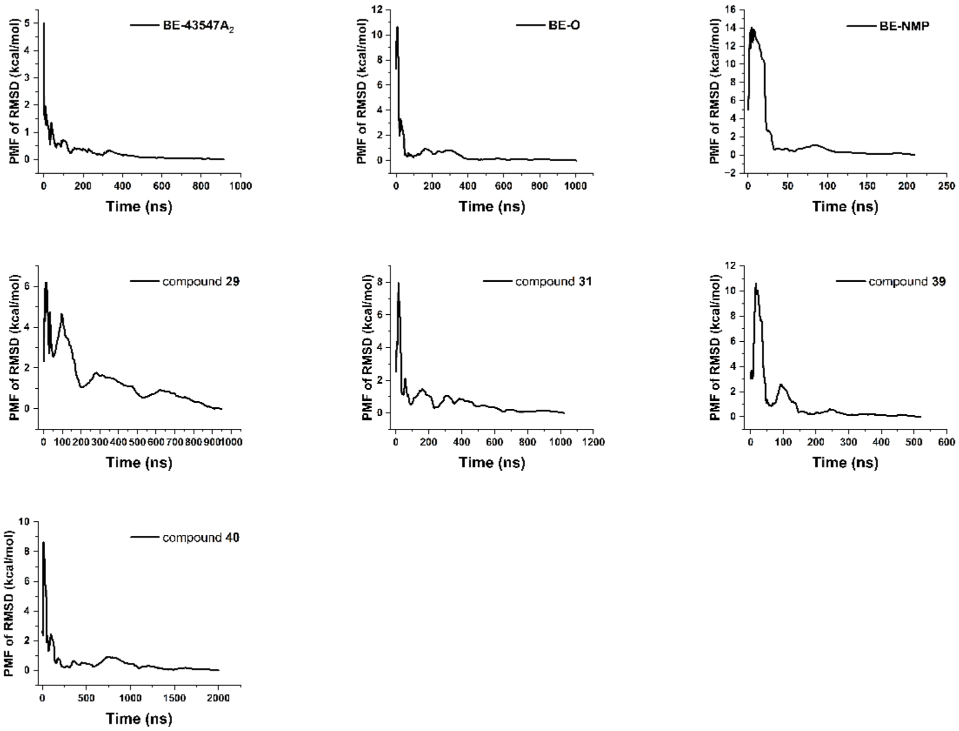

Supplementary Figure S2. Convergence of the PMF calculated from WTM-eABF simulation.

3. Simulation times of the equilibration and free-energy calculations for the BE derivative-POPC systems

Supplementary Table S2. Simulation times of the equilibration and free-energy calculations.

Permeants	Equilibration time (μs)	WTM-eABF simulation time (μs)
BE-43547A2	1.0	0.9
BE-O	1.0	1.0
BE-NMP	1.0	0.2
**29**	1.0	2.3
**31**	1.0	1.0
**39**	1.0	0.8
**40**	1.0	4.0

4. Procedure for log *D* determinations

Procedure for log *D* determinations of BE: 3 μL of 10 mM stock solution is diluted with the addition of 97 μL of phosphate buffer saturated 1-octanol to get a concentration of 300 μM working solution. 20 μL of 300 μM compound solution is placed in order into their proper 96-well rack followed by the addition of 980 μL of 1-octanol saturated phosphate buffer. One stir stick is added to each vial and vials are sealed molded PTFE/Silicone plugs. Then the log *D* plate is transferred to the Eppendorf Thermomixer Comfort plate shaker and shaken at 25°C at 1,100 RPM for 1 h. The assay is performed in duplicate. After completion of 1 h, the stir sticks are removed using a big magnet. The samples are then centrifuged at 25°C at 20,000 g for 20 minutes to separate the phases, and syringe is used to remove the lower (buffer) phases to the empty tubes. Then the buffer phase is diluted with the same volume of acetonitrile/ultrapure water (50%/50%) for LC-MS/MS analysis. 300 μM working solution is diluted with acetonitrile/ultrapure water (50%/50%) to achieve a concentration of 3 μM. Then the 3 μM solution is diluted to 0.3 μM to get the total samples by adding 4 volumes of acetonitrile/ultrapure water (50%/50%) and 5 volumes of 1-octanol saturated Phosphate Buffer for LC-MS/MS analysis.

Procedure for log *D* determinations of five additional compounds: 15 μL of stock solutions (10 mM) of test compounds and control compound is placed in order into their 2.0 mL deep well plate, 500 μL of saturated 1-octanol is added into each vial of the log *D* plate vortex for 2 minutes at 1,000 rpm, and then followed by the addition of 500 μL of saturated PBS (pH 7.4). Seal the plate using a Silicone sealing mat. Then the log *D* plate is transferred to the Eppendorf Thermomixer Comfort plate shaker and shaken at 25°C at 2,000 RPM for 2 h. After completion of 2 h, the samples are then centrifuged at 25°C at 3,220 g for 30 minutes to separate the phases, and pipette are used to remove the upper (1-octanol) and lower (buffer) phases to the empty tubes, respectively. Aliquots of 50 μL are taken from upper phases followed by addition of 450 μL of acetonitrile (containing internal standards (IS, 200 nM labetalol, 100 nM tolbutamide and 100 nM ketoprofen)). Mix with a pipette, and then aliquots of 50 μL are taken from the diluent followed by addition of 450 μL of acetonitrile (containing internal standards (IS, 200 nM labetalol, 100 nM tolbutamide and 100 nM ketoprofen)). Mix with a pipette, and final aliquots of 30 μL are taken from the diluent followed by addition of 120 μL of acetonitrile (containing internal standards (IS, 200 nM labetalol, 100 nM tolbutamide and 100 nM ketoprofen)) 120 μL of H_2O and 30 μL of saturated PBS. And aliquots of 50 μL are taken from lower phases followed by addition of 249.5 μL of acetonitrile (containing internal standards (IS, 200 nM labetalol, 100 nM tolbutamide and 100 nM ketoprofen)) 0.5 μL of saturated 1- Octanol and 200 μL of H_2O. A certain proportion of ultrapure water maybe used according to the peak shape. The

dilution factor is changed according to the log D value and the LC-MS signal response. The samples are evaluated by LC-MS/MS analysis. All experiments are performed in duplicates.

5. Caco-2 Cell Permeability Assay

The Caco-2 plate is removed from the incubator and washed twice with pre-warmed HBSS (10 mM HEPES, pH 7.4), and then incubated at 37 °C for 30 minutes. The stock solutions of control compounds and test compounds are diluted in DMSO to get 1 mM solutions and then diluted with HBSS (10 mM HEPES, pH 7.4) get 5 μM working solutions. The final concentration of DMSO in the incubation system is 0.5%. To determine the rate of drug transport in the apical to basolateral direction. 125 μL of 5 μM working solution of control compounds and test compounds are added to the Transwell insert (apical compartment), and transfer 50 μL sample (D0 sample) immediately from the apical compartment to a new 96-well plate. Fill the wells in the receiver plate (basolateral compartment) with 235 μL of HBSS (10 mM HEPES, pH 7.4). To determine the rate of drug transport in the basolateral to apical direction. 285 μL of 5 μM working solution of control compounds and test compounds are to the receiver plate wells (basolateral compartment), and transfer 50 μL sample (D0 sample) immediately from the basolateral compartment to a new 96-well plate. Fill the wells in the Transwell insert (apical compartment) with 75 μL of HBSS (10 mM HEPES, pH 7.4). The assay is performed in duplicate. The plates are incubated at 37 °C for 2 h. At the end of the incubation, 50 μL samples from donor sides (apical compartment for Ap→Bl flux, and basolateral compartment for Bl→Ap) and receiver sides (basolateral compartment for Ap→Bl flux, and apical compartment for Bl→Ap) are transferred to wells of a new 96-well plate, followed by the addition of 4 volume of cold acetonitrile containing appropriate internal standards (IS). Samples are Vortexed for 5 minutes and then centrifuged at 3,220 g for 30 minutes. An aliquot of 100 μL of the supernatant is mixed with an appropriate volume of ultra-pure water before LC-MS/MS analysis (see SI Bioanalytical Method for Caco-2 Cell Permeability Assay). If the $P_{app\ (A\ to\ B)}$ of the test article is less than atenolol it is assigned as low permeability and if the $P_{app\ (A\ to\ B)}$ is greater than the $P_{app\ (A\ to\ B)}$ of minoxidil it is assigned as high permeability. If the $P_{app\ (A\ to\ B)}$ is greater than $P_{app\ (A\ to\ B)}$ of atenolol and less than the $P_{app\ (A\ to\ B)}$ of minoxidil it is assigned as moderate permeability. If the efflux ratio of test article is greater than or equal to 2, it is considered a potential substrate of efflux transporters.

The apparent permeability coefficient (P_{app}), in units of centimeter per second, can be calculated for Caco-2 drug transport assays using the following equation:

$$P_{app} = (V_A \times [drug]_{acceptor})/(Area \times Time \times [drug]_{initial,donor})$$

where V_A is the volume (in mL) in the acceptor well, Area is the surface area of the membrane (0.143 cm^2 for Transwell-96 Well Permeable Supports), and time is the total transport time in seconds.

The recovery can be determined using the following equation:

$$Recovery\% = (V_A \times [drug]_{acceptor} + V_D \times [drug]_{donor})/(V_D \times [drug]_{initial,\ donor})$$

where V_A is the volume (in mL) in the acceptor well (0.235 mL for Ap→Bl flux, and 0.075 mL for Bl→Ap), V_D is the volume (in mL) in the donor well (0.075 mL for Ap→Bl flux, and 0.235 mL for Bl→Ap)

6. The Assessment of Caco-2 Cell Monolayer Integrity

To determine the Lucifer Yellow leakage after 2 hours transport period, stock solution of Lucifer yellow is prepared in water and diluted with HBSS (10 mM HEPES, pH 7.4) to reach the final concentration of 100 μM. 100 μL of the Lucifer yellow solution is added to each Transwell insert (apical compartment), followed by filling the wells in the receiver plate (basolateral compartment) with 300 μL of HBSS (10 mM HEPES, pH 7.4). The plates are Incubated at 37 °C for 30 mins. 80 μL samples are removed directly from the apical and basolateral wells (using the basolateral access holes) and transferred to wells of new 96 wells plates.

The Lucifer Yellow fluorescence (to monitor monolayer integrity) signal is measured in a fluorescence plate reader at 485 nM excitation and 530 nM emission.
The leakage of Lucifer Yellow, in unit of percentage (%), can be calculated using the following equation:

$$\%LY\ leakage = 100\times[LY]_{acceptor}/([LY]_{donor}+[LY]_{acceptor})$$

Where $[LY]_{acceptor}$ is the fluorescence intensity in the acceptor well (0.3 mL), and $[LY]_{donor}$ is the fluorescence intensity in the donor well (0.1 mL) and expressed as % leakage. The percentage of Lucifer yellow leakage should be less than 1.0%. If leakage in one parallel ranges from 1.0% to 1.5%, the P_{app} data from the replicate monolayers will be compared. If the P_{app} value in the transwell with >1% LY flux is qualitatively similar to those in the replicate transwells (CV<30%), the monolayer's P_{app} data will be considered valid and acceptable.

Supplementary Table S3. The assessment of caco-2 cell monolayer integrity.

Compound	Concentration (μM)	TEER $_{A-B}$ (Ω× cm^2)	TEER $_{B-A}$ (Ω× cm^2)	LY Leakage $_{A-B}$ (%)	LY Leakage $_{B-A}$ (%)
BE	5	570	596	0.21	0.19
BE-NMP	5	486	535	0.32	0.20
**29**	5	566	578	0.21	0.17
**31**	5	583	622	0.20	0.18
**39**	5	527	555	0.56	0.17
**40**	5	569	586	0.22	0.18
Atenolol	5	597	576	0.21	0.21
Digoxin	5	509	493	0.21	0.22
Minoxidil	5	510	539	0.20	0.20

7. MST Binding Assays: Detailed Materials and Protein Labeling Procedures

7.1 Materials

Recombinant human eEF1A1 (54.1 kDa; catalog no. TMPH-01283) was used as the target protein. The ligands tested included BE, Compound **29** and Compound **31**. Additional materials comprised the RED-NHS protein labeling kit (NanoTemper Technologies, cat. no. MO-L011), premium-coated capillaries (NanoTemper Technologies, cat. no. MO-K022), HEPES buffer (pH 7.4), and assay buffer (50 mM HEPES, pH 7.4, containing 0.05% Tween-20). The NanoTemper Monolith X instrument (NanoTemper Technologies, Munich, Germany) was used for all measurements.

7.2 Protein Labeling and Preparation

The eEF1A1 protein was exchanged into labeling buffer using a desalting column. The column was equilibrated with 300 μL labeling buffer three times by centrifugation at 3,000 rpm for 30 s each. The protein (50 μL at 5 μM) was then applied to the column and centrifuged at 3,000 rpm for 60 s to elute into labeling buffer. The eluted protein was adjusted to a final concentration of 5 μM in 70 μL labeling buffer.

Dye solution was prepared by dissolving the RED-NHS dye in 30 μL 100% DMSO to yield a 470 μM stock, then diluted in labeling buffer to 100 μL at a concentration of 15 μL to ensure excess dye for labeling. The protein (70 μL) was mixed with dye solution (70 μL) and incubated in the dark at room temperature for 30 min.

Excess dye was removed using a molecular sieve column equilibrated with 3 mL assay buffer three times under gravity flow. The labeled protein mixture (140 μL) was applied, followed by 300 μL assay buffer to

wash out unbound dye. Labeled protein was eluted with 600 μL assay buffer, yielding a stock solution at approximately 0.83 μM.

8. Experimental section

8.1 Chemistry. General

^{1}H NMR, ^{13}C NMR were recorded on Bruker AV 400 MHz or 600 MHz, calibrated by using internal references and solvent signals $CHCl_3$ (δH = 7.26 ppm, δC = 77.16 ppm), DMSO-d_6 (δH = 2.50 ppm, δC = 39.52 ppm). ^{1}H NMR data are reported as follows: chemical shift, multiplicity (s = singlet, d = doublet, dd = doublet of doublets, t = triplet, td = triplet of doublets, dt = doublet of triplets, q = quartet, m = multiplet), coupling constants and integration. High-resolution mass spectra (HRMS) were obtained with WATERS Xevo G2-XS Q-TOF. The purity of representative final compounds was evaluated on a Shimadzu HPLC system with an ODS-C18 column (4.6 × 150 mm, 5 μm) eluted at 1 mL/min with Milli-Q water and CH_3CN. All final compounds and the key intermediate **17** are stable for more than 18 months when stored as solids at –80 °C.

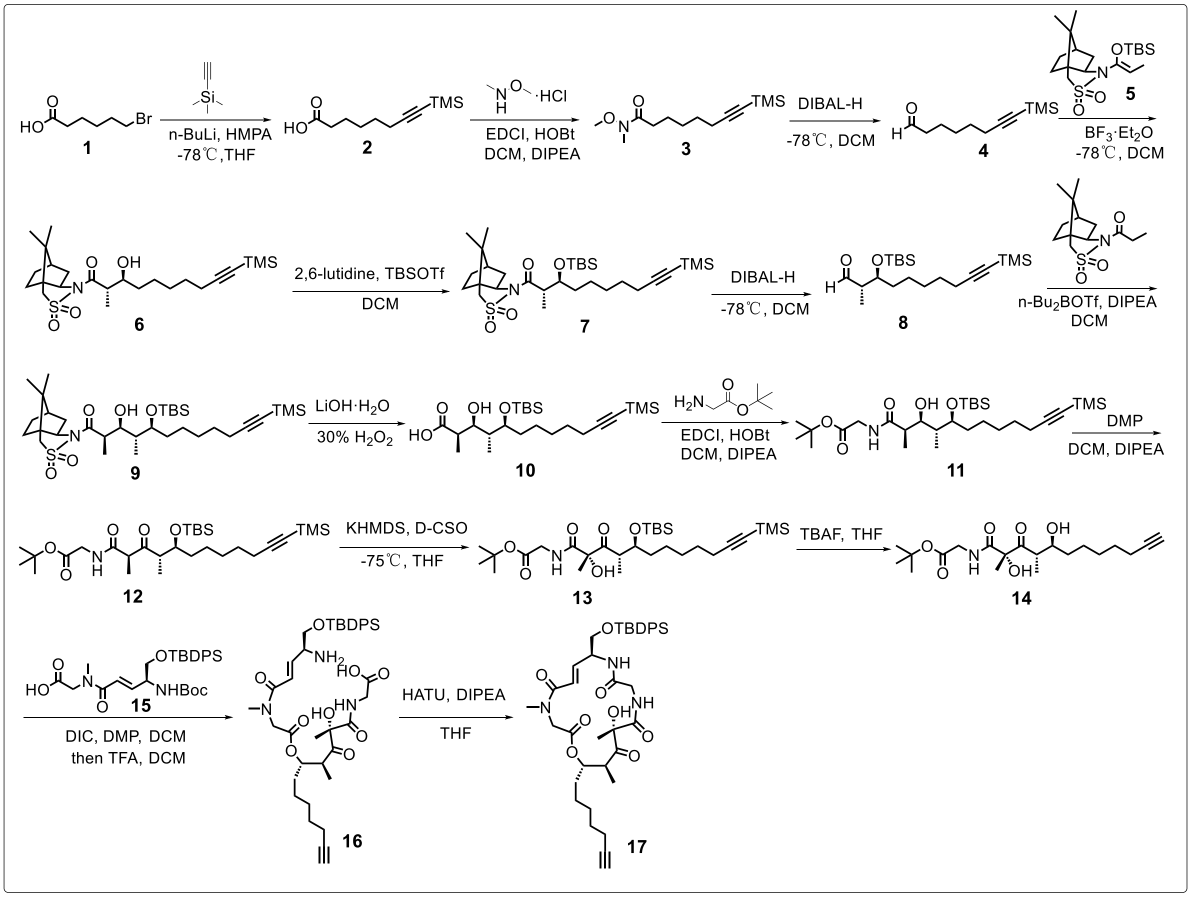

Supplementary Scheme S1. Synthesis of Intermediate **17**.

6-bromohexanoic acid (**2**). Trimethylsilylacetylene (90.00 g, 916.31 mmol) and anhydrous THF (300 mL) were added to a 2 L three-necked flask and stirred until a clear solution was obtained. Under a nitrogen atmosphere, the solution was cooled to -78°C, and a 2.5 M solution of n-butyllithium (380 mL) was added dropwise. After the addition was complete, the mixture was stirred for 0.5 h. A THF solution of **1** (63.84 g, 327.30 mmol) and HMPA (255.0 mL) was then added dropwise, and the reaction was stirred at -75°C for

1h. The reaction mixture was transferred to a -20°C cooling bath and stirred for an additional 2.5 h. A saturated aqueous ammonium chloride solution (300 mL) was added dropwise, allowing the mixture to warm to room temperature, and stirring was continued overnight. The layers were separated, and the aqueous phase was adjusted to *p*H = 2-3 using 1 M hydrochloric acid and extracted with ethyl acetate (2 × 100 mL). The combined organic layers were washed with saturated brine, dried over anhydrous sodium sulfate, filtered, and concentrated under reduced pressure. The crude product was purified by silica gel chromatography (PE: EA= 10:1) to give **2** (66.60 g, 94.96%) as a yellow oily substance. ^{1}H NMR (600 MHz, $CDCl_3$) δ 2.36 (t, *J* = 7.5 Hz, 2H), 2.23 (t, *J* = 7.1 Hz, 2H), 1.65 (dt, *J* = 15.2, 7.5 Hz, 2H), 1.53 (dd, *J* = 10.3, 4.3 Hz, 2H), 1.47 – 1.41 (m, 2H), 0.14 (s, 9H); ^{13}C NMR (101 MHz, DMSO-d_6) δ 174.2, 107.7, 84.0, 33.4, 27.6, 27.5, 23.8, 18.8; HRMS (ESI) m/z: calcd for $C_{11}H_{21}O_2Si^+$ [M+H]$^+$: 213.1305, found: 213.1307.

N-methoxy-N-methyl-8-(trimethylsilyl)oct-7-ynamide (**3**). **2** (67.04 g, 315.69 mmol) and DCM (700 mL) were added to a 2 L three-necked flask, followed by the sequential addition of EDCI (72.96 g, 378.82 mmol) and HOBt (51.16 g, 378.83 mmol). The mixture was stirred under nitrogen for 0.5 h. The temperature was lowered to 10°C, and *N*-methoxymethylamine hydrochloride (33.85 g, 347.26 mmol) was added in one portion. DIPEA (61.16 g, 473.54 mmol) was then added dropwise, and the mixture was allowed to warm to room temperature naturally. After adding 200 mL of dichloromethane and 100 mL of water, the mixture was stirred and the layers were separated. The organic layer was washed with water (2 × 100 mL), combined, dried over anhydrous sodium sulfate, filtered, and concentrated under reduced pressure. The crude product was purified by silica gel chromatography (PE: EA = 5:1) to give **3** (57.0 g, 70.74%) as a yellow oily substance. ^{1}H NMR (600 MHz, $CDCl_3$) δ 3.67 (s, 3H), 3.17 (s, 3H), 2.42 (t, *J* = 7.7 Hz, 2H), 2.22 (t, *J* = 7.2 Hz, 2H), 1.67 – 1.61 (m, 2H), 1.53 (q, *J* = 7.4 Hz, 2H), 1.45 – 1.40 (m, 2H), 0.13 (s, 9H); LCMS: retention time 7.44 min, *m/z* = 256.26 [M+H]$^+$.

8-(trimethylsilyl)oct-7-ynal (**4**). **3** (30.00 g, 117.45 mmol) and DCM (300 mL) were added to a 1 L round-bottom flask and stirred until a clear solution was obtained. Under a nitrogen atmosphere, the mixture was cooled to -75°C. DIBAL-H (118.0 mL, 176.17 mmol) was added dropwise, and the reaction was stirred for 1 h. Methanol (20 mL) was then added dropwise, allowing the mixture to warm to room temperature. A saturated potassium sodium tartrate solution (120 mL) was added, and the mixture was stirred overnight. The layers were separated, and the aqueous phase was extracted with ethyl acetate (2 × 100 mL). The combined organic layers were dried over anhydrous sodium sulfate, filtered, and concentrated under reduced pressure. The crude product was purified by silica gel chromatography (PE: EA = 10:1) to obtain **4** (18.44 g, 79.97%) as a yellow oily substance. ^{1}H NMR (600 MHz, $CDCl_3$) δ 9.76 (s, 1H), 2.48 – 2.36 (m, 2H), 2.22 (t, *J* = 7.1 Hz, 2H), 1.67 – 1.61 (m, 2H), 1.55 – 1.50 (m, 2H), 1.44 – 1.40 (m, 2H), 0.13 (s, 9H).

(2S,3S)-1-((6R,7aR)-8,8-dimethyl-2,2-dioxidotetrahydro-3H-3a,6-methanobenzo[c]isothiazol-1(4H)-yl)-3-hydroxy-2-methyl-10-(trimethylsilyl)dec-9-yn-1-one (**6**). Compound **4** (5.00 g, 25.49 mmol) and DCM (50 mL) were added to a 250 mL three-necked flask and stirred under nitrogen while cooling to -70°C. A solution of $BF_3{\cdot}Et_2O$ (5.43 g, 38.24 mmol) was added dropwise, and the reaction was stirred for 0.5 h. A solution of **5** (14.75 g, 38.24 mmol) in DCM (see ref. 2[3] for the synthesis **5**) was then added dropwise, and the reaction was stirred for an additional 1.5 h. A saturated sodium bicarbonate solution (40 mL) was added dropwise, and the mixture was separated. The aqueous layer was extracted with a small amount of DCM. The combined organic layers were dried over anhydrous sodium sulfate, filtered, and concentrated under reduced pressure. The crude product was purified by silica gel chromatography (PE: EA = 5:1) to yield **6** (5.41 g, 45.46%) as a colorless transparent oily substance. ^{1}H NMR (600 MHz, $CDCl_3$) δ 3.90 (dd, *J* = 7.8, 4.9 Hz, 1H), 3.52 (d, *J* = 13.8 Hz, 1H), 3.45 (d, *J* = 13.9 Hz, 1H), 3.18 (q, *J* = 6.6 Hz, 1H), 2.20 (t, *J* = 7.2 Hz, 2H),

2.14 (q, J = 4.6 Hz, 1H), 2.10 – 2.03 (m, 2H), 2.01 – 1.90 (m, 2H), 1.90 – 1.85 (m, 2H), 1.60 – 1.55 (m, 1H), 1.50 (dd, J = 10.2, 4.9 Hz, 2H), 1.47 – 1.40 (m, 1H), 1.39 (d, J = 4.6 Hz, 1H), 1.38 (d, J = 3.7 Hz, 2H), 1.36 – 1.34 (m, 2H), 1.33 (d, J = 4.5 Hz, 1H), 1.23 (d, J = 6.7 Hz, 3H), 1.18 (s, 3H), 0.97 (s, 3H), 0.14 (s, 9H); ^{13}C NMR (101 MHz, DMSO-d_6) δ 174.1, 107.3, 83.5, 72.5, 66.8, 64.2, 52.3, 47.7, 47.0, 44.2, 37.9, 33.2, 31.9, 28.1, 28.0, 25.9, 24.3, 20.5, 19.4, 19.0, 12.4; HRMS (ESI) m/z: calcd for $C_{24}H_{42}NO_4SSi^+$ $[M+H]^+$: 468.2598, found: 468.2596.

(2S,3S)-3-((tert-butyldimethylsilyl)oxy)-1-((6R,7aR)-8,8-dimethyl-2,2-dioxidotetrahydro-3H-3a,6-methanobenzo[c]isothiazol-1(4H)-yl)-2-methyl-10-(trimethylsilyl)dec-9-yn-1-one (**7**). Compound **6** (24.74 g, 52.90 mmol) and DCM (520 mL) were added to a 1 L three-necked flask, stirred under a nitrogen atmosphere, and cooled to 5°C. 2,6-lutidine (10.0 mL) was added dropwise, followed by the dropwise addition of TBSOTf (21.0 mL). The mixture was allowed to warm to room temperature and stirred overnight. Et_3N (6.0 mL) and MeOH (12.0 mL) were then added, and the mixture was stirred and washed three times with 1 M hydrochloric acid. The organic layer was dried over anhydrous sodium sulfate, filtered, and concentrated under reduced pressure. The crude product was purified by silica gel chromatography (PE: EA = 5:1) to obtain **7** (25.26 g, 82.07%) as a white solid. ^{1}H NMR (600 MHz, $CDCl_3$) δ 4.10 (td, J = 6.7, 2.7 Hz, 1H), 3.88 (dd, J = 7.7, 4.8 Hz, 1H), 3.48 (d, J = 13.7 Hz, 1H), 3.42 (d, J = 13.8 Hz, 1H), 3.27 (q, J = 6.6 Hz, 1H), 2.20 (t, J = 7.2 Hz, 2H), 2.06 (dd, J = 13.8, 7.7 Hz, 1H), 2.02 – 1.97 (m, 1H), 1.92 (dd, J = 12.0, 4.2 Hz, 1H), 1.89 – 1.85 (m, 2H), 1.57 – 1.51 (m, 2H), 1.49 (dd, J = 10.2, 4.9 Hz, 2H), 1.42 – 1.38 (m, 2H), 1.37 – 1.34 (m, 2H), 1.32 – 1.28 (m, 2H), 1.16 (s, 3H), 1.11 (d, J = 6.7 Hz, 3H), 0.97 (s, 3H), 0.94 (t, J = 8.0 Hz, 9H), 0.61 (dd, J = 7.9, 4.2 Hz, 6H), 0.14 (s, 9H).

(2S,3S)-3-((tert-butyldimethylsilyl)oxy)-2-methyl-10-(trimethylsilyl)dec-9-ynal (**8**). Compound **7** (25.26 g, 43.40 mmol) and DCM (250 mL) were added to a 500 mL three-necked flask under a nitrogen atmosphere and cooled to -78°C. DIBAL-H (65.0 mL, 65.10 mmol) was added dropwise, and the reaction was maintained at -75°C for 1 h. Methanol (15 mL) was then added dropwise, allowing the mixture to warm to room temperature, followed by the addition of 80 mL of saturated potassium sodium tartrate solution. The layers were separated, and the aqueous phase was extracted with DCM (2 × 100 mL). The combined organic layers were dried over anhydrous sodium sulfate, filtered, and concentrated under reduced pressure. The concentrate was triturated with 200 mL of PE, filtered, and concentrated. The crude product was purified by silica gel chromatography (PE: EA = 15:1) to give **8** (14.93 g, 93.31%) as a colorless transparent oily substance. ^{1}H NMR (600 MHz, $CDCl_3$) δ 9.74 (d, J = 2.3 Hz, 1H), 3.95 (dt, J = 6.2, 5.1 Hz, 1H), 2.52 – 2.46 (m, 1H), 2.22 (t, J = 7.2 Hz, 2H), 1.59 – 1.52 (m, 2H), 1.51 – 1.45 (m, 2H), 1.44 – 1.37 (m, 2H), 1.36 – 1.33 (m, 2H), 1.07 (dd, J = 13.9, 7.0 Hz, 3H), 0.95 (t, J = 8.0 Hz, 6H), 0.60 (q, J = 8.0 Hz, 6H), 0.14 (s, 9H).

(2R,3S,4R,5S)-5-((tert-butyldimethylsilyl)oxy)-1-((6R,7aR)-8,8-dimethyl-2,2-dioxidotetrahydro-3H-3a,6-methanobenzo[c]isothiazol-1(4H)-yl)-3-hydroxy-2,4-dimethyl-12-(trimethylsilyl)dodec-11-yn-1-one (**9**). *N*-Propionyl-(2R)-bornane-10,2-sultam (10.99 g, 40.49 mmol) and DCM (1500 mL) were added to a 500 mL three-necked flask and stirred to form a clear solution. Under a nitrogen atmosphere, the solution was cooled to 0°C. A solution of dibutylboryl trifluoromethanesulfonate (n-Bu_2BOTf) (49.00 mL, 48.59 mmol) was added dropwise, followed by the dropwise addition of DIPEA (6.54 g, 50.62 mmol), and the mixture was stirred for 0.5 h. A DCM solution of **8** (14.93 g, 40.49 mmol) was then added dropwise, and the reaction was stirred for an additional hour. A saturated ammonium chloride solution (100 mL) was added dropwise, and the mixture was allowed to warm to room temperature while stirring. The aqueous layer was extracted with 100 mL of DCM. The combined organic layers were dried over anhydrous sodium sulfate, filtered, and concentrated under reduced pressure. The crude product was purified by silica gel chromatography (PE: EA = 15:1) to give **9** (22.10 g, 85.26%) as a colorless transparent oily substance. ^{1}H NMR (600 MHz,

$CDCl_3$) δ 4.07 (dd, *J* = 10.0, 5.1 Hz, 1H), 3.90 – 3.87 (m, 1H), 3.51 (d, *J* = 13.9 Hz, 1H), 3.44 (d, *J* = 13.7 Hz, 1H), 3.22 – 3.17 (m, 1H), 2.23 – 2.19 (m, 2H), 2.10 – 2.03 (m, 2H), 1.96 – 1.88 (m, 2H), 1.87 (d, *J* = 3.7 Hz, 1H), 1.66 (dd, *J* = 11.2, 2.3 Hz, 1H), 1.54 (d, *J* = 3.0 Hz, 2H), 1.53 – 1.50 (m, 2H), 1.50 – 1.45 (m, 1H), 1.41 (d, *J* = 9.2 Hz, 2H), 1.36 (s, 2H), 1.34 – 1.31 (m, 2H), 1.31 – 1.29 (m, 1H), 1.27 (d, *J* = 7.0 Hz, 3H), 1.16 (d, *J* = 6.5 Hz, 3H), 0.99 – 0.94 (m, 9H), 0.89 – 0.84 (m, 3H), 0.84 – 0.72 (m, 3H), 0.61 – 0.58 (m, 3H), 0.14 (s, 9H), -0.00 (s, 3H).

(2R,3S,4R,5S)-5-((tert-butyldimethylsilyl)oxy)-3-hydroxy-2,4-dimethyl-12-(trimethylsilyl)dodec-11-ynoic acid (**10**). **9** (22.10 g, 34.37 mmol) was added to a 500 mL three-necked flask with a solvent mixture of THF: MeOH: H_2O in a 154.0: 25.0: 20.0 (mL) ratio and stirred to form a clear solution. Under a nitrogen atmosphere, the solution was cooled to 0°C. $LiOH{\cdot}H_2O$ (5.77 g, 137.48 mmol) was added in one portion, followed by the dropwise addition of 30% H_2O_2 (38.95 g, 343.70 mmol). After the addition was complete, the mixture was stirred for 1 h. A saturated sodium thiosulfate solution (60 mL) was added dropwise, causing vigorous heating. The *p*H was adjusted to 2-3 with 1 M hydrochloric acid, and the mixture was extracted with ethyl acetate (2 × 150 mL). The combined organic layers were washed with saturated brine, dried over anhydrous sodium sulfate, filtered, and concentrated under reduced pressure. The residue was triturated with a mixture of PE: EA = 15:1 (10 times the volume), filtered, and concentrated to yield 20.01 g of a yellow oily substance. The crude product **10** was used directly in the next step; LCMS: retention time 11.96 min, *m/z* = 443.48 $[M+H]^+$.

tert-butyl ((2R,3S,4R,5S)-5-((tert-butyldimethylsilyl)oxy)-3-hydroxy-2,4-dimethyl-12-(trimethylsilyl)dodec-11-ynoyl)glycinate (**11**). The aforementioned hydrolysis product **10** (obtained as described above) was added to a 500 mL three-necked flask containing a DCM (150 mL) solution and stirred until clear. Glycine tert-butyl ester (6.76 g, 51.56 mmol) was added, and the mixture was cooled to 0°C under a nitrogen atmosphere and stirred for 15 minutes. EDCI (7.90 g, 41.24 mmol), HOBt (5.57 g, 41.24 mmol), and DIPEA (5.33 g, 41.24 mmol) were then added sequentially, and the reaction was stirred for 3 h. Silica gel was added to the mixture for preparation, and the product was purified by silica gel chromatography (PE: EA = 5:1) to yield **11** (15.00 g, 78.49% for two steps) as a pale yellow transparent oily substance. ^1H NMR (600 MHz, $CDCl_3$) δ 3.95 (dd, *J* = 18.2, 5.4 Hz, 2H), 3.88 (dd, *J* = 18.2, 5.0 Hz, 1H), 3.75 (dd, *J* = 9.7, 1.8 Hz, 1H), 2.46 (dd, *J* = 7.2, 1.8 Hz, 1H), 2.21 (t, *J* = 7.2 Hz, 2H), 1.79 – 1.74 (m, 1H), 1.52 (t, *J* = 7.2 Hz, 2H), 1.46 (s, 9H), 1.44 – 1.43 (m, 1H), 1.42 – 1.38 (m, 2H), 1.37 – 1.32 (m, 2H), 1.18 (d, *J* = 7.2 Hz, 3H), 0.96 (t, *J* = 7.9 Hz, 9H), 0.76 (d, *J* = 6.9 Hz, 3H), 0.63 (q, *J* = 7.9 Hz, 6H), 0.14 (s, 9H).

tert-butyl ((2R,4S,5S)-5-((tert-butyldimethylsilyl)oxy)-2,4-dimethyl-3-oxo-12-(trimethylsilyl)dodec-11-ynoyl)glycinate (**12**). **11** (15.00 g, 27.01 mmol) was added to a 500 mL three-necked flask with DCM (220 mL) and stirred until a clear solution was formed. Under a nitrogen atmosphere, the solution was cooled to 0°C. DMP (17.18 g, 40.51 mmol) was added in one portion, and the reaction mixture was stirred for 1 h. A saturated sodium bicarbonate solution (80 mL) and a saturated sodium thiosulfate solution (80 mL) were added dropwise, followed by stirring and phase separation. The aqueous phase was extracted with DCM (2 × 100 mL). The combined organic layers were dried over anhydrous sodium sulfate, filtered, and concentrated under reduced pressure. The crude product was purified by silica gel chromatography (PE: EA = 6:1) to give **12** (12.12 g, 81.02%) as a yellow oily substance. ^1H NMR (600 MHz, $CDCl_3$) δ 3.98 – 3.93 (m, 1H), 3.91 (d, *J* = 9.5 Hz, 1H), 3.89 – 3.83 (m, 1H), 3.57 (q, *J* = 7.2 Hz, 1H), 2.99 – 2.92 (m, 1H), 2.23 – 2.19 (m, 2H), 1.57 – 1.50 (m, 2H), 1.50 – 1.47 (m, 2H), 1.46 (d, *J* = 2.1 Hz, 9H), 1.41 (d, *J* = 7.3 Hz, 2H), 1.34 (d, *J* = 7.2 Hz, 3H), 1.33 – 1.23 (m, 2H), 1.00 (dd, *J* = 24.6, 6.9 Hz, 3H), 0.92 (td, *J* = 7.9, 2.7 Hz, 9H), 0.56 (q, *J* = 7.9 Hz, 6H), 0.13 (s, 9H).

tert-butyl ((2S,4S,5S)-5-((tert-butyldimethylsilyl)oxy)-2-hydroxy-2,4-dimethyl-3-oxo-12-(trimethylsilyl)dodec-11-ynoyl)glycinate (**13**). **12** (4.32 g, 7.64 mmol) was added to a 250 mL three-necked flask with DCM and stirred until a clear solution was obtained. Under a nitrogen atmosphere, the solution was cooled to -75°C. KHMDS (2.30 mL, 2.29 mmol) was added dropwise, followed by the dropwise addition of a THF solution (60 mL) of D-CSO (1.75 g, 7.64 mmol). After the additions were complete, the reaction was stirred for 1 h. A saturated ammonium chloride solution (20 mL) was added dropwise, and the mixture was allowed to warm to room temperature. Water (10 mL) was added, and the mixture was stirred and separated. The aqueous phase was extracted with ethyl acetate (2 × 20 mL). The combined organic layers were washed with saturated brine, dried over anhydrous sodium sulfate, filtered, and concentrated under reduced pressure. The crude product was purified by silica gel chromatography (PE: EA = 15:1) to yield **13** (3.20 g, 73.56%) as a colorless transparent oily substance; LCMS: retention time 8.56 min, *m/z* = 400.38 $[M-170]^+$.

tert-butyl ((2S,4S,5S)-2,5-dihydroxy-2,4-dimethyl-3-oxododec-11-ynoyl)glycinate (**14**). Compound **13** (10.30 g, 18.07 mmol) was added to a 250 mL three-necked flask containing THF (80.0 mL) and stirred until a clear solution was formed. Under a nitrogen atmosphere, the solution was cooled to 0°C. A THF solution of TBAF (11.81 g, 22.59 mmol) was added dropwise, and the mixture was allowed to warm to room temperature, stirring for an additional 2 h. A saturated ammonium chloride solution (40 mL) was added dropwise, followed by the addition of 20 mL of water. The mixture was stirred and separated, and the aqueous phase was extracted with ethyl acetate (2 × 20 mL). The combined organic layers were washed with saturated brine, dried over anhydrous sodium sulfate, filtered, and concentrated under reduced pressure. The crude product was purified by silica gel chromatography (PE: EA = 3:1) to obtain **14** (6.48 g, 93.51%) as off-white crystals. ^{1}H NMR (600 MHz, $CDCl_3$) δ 7.43 (t, *J* = 5.5 Hz, 1H), 3.89 (dd, *J* = 18.0, 5.2 Hz, 1H), 3.81 (dd, *J* = 18.0, 5.6 Hz, 1H), 3.59 – 3.53 (m, 2H), 2.16 (td, *J* = 7.0, 2.7 Hz, 2H), 1.92 (t, *J* = 2.6 Hz, 1H), 1.58 (d, *J* = 2.5 Hz, 1H), 1.56 (s, 3H), 1.54 – 1.51 (m, 2H), 1.50 – 1.47 (m, 2H), 1.45(s, 9H), 1.37 – 1.33 (m, 2H), 1.29 (d, *J* = 28.0 Hz, 1H), 1.06 (d, *J* = 6.2 Hz, 3H); LCMS: retention time 6.10 min, *m/z* = 384.24 $[M+H]^+$; HPLC purity: 95.25%.

((6S,14S,15S,17S,E)-6-amino-14-(hept-6-yn-1-yl)-17-hydroxy-2,2,10,15,17-pentamethyl-9,12,16-trioxo-3,3-diphenyl-4,13-dioxa-10-aza-3-silaoctadec-7-en-18-oyl)glycine (**16**). To a solution of compound **14** (2.00 g, 5.21 mmol) and carboxylic acid **15** (4.23 g, 7.82 mmol) in 55 mL of DCM (see ref. 2[3] for the synthesis **15**), stirred at 0 ºC under a nitrogen atmosphere, were added DMAP (0.13 g, 1.04 mmol) and DIC (1.32 g, 10.43 mmol). The reaction mixture was stirred for 3 h, after which 50 mL of water was added, and the layers were separated. The aqueous phase was extracted with DCM (2 × 50 mL), and the combined organic phases were dried over Na_2SO_4 and concentrated under reduced pressure. The crude product was purified by silica gel chromatography (DCM: MeOH=40:1) to obtain a yellow foamy solid (4.16 g, 86.67%). This solid (4.16 g, 4.52 mmol), as obtained above, was dissolved in 32 mL of DCM, and trifluoroacetic acid (TFA, 8.0 mL) was added under a nitrogen atmosphere. The mixture was stirred at room temperature overnight. Subsequently, 50 mL of toluene was added, and the mixture was evaporated under reduced pressure to remove TFA and toluene, yielding a dark red viscous liquid. The crude product **16** was used in the subsequent step without further purification; LCMS: retention time 6.88 min, *m/z* = 750.52 $[M+H]^+$.

(8S,14S,16S,17S,E)-8-(((tert-butyldiphenylsilyl)oxy)methyl)-17-(hept-6-yn-1-yl)-14-hydroxy-4,14,16-trimethyl-1-oxa-4,9,12-triazacycloheptadec-6-ene-2,5,10,13,15-pentaone (**17**). To a solution of HATU (8.59 g, 22.60 mmol) and DIPEA (5.84 g, 45.52 mmol) in 410 mL of anhydrous THF, stirred at room temperature under a nitrogen atmosphere, a solution of the dark red viscous liquid (obtained as described above, in 80.0 mL of anhydrous THF) was added dropwise over 12 h. The reaction mixture was stirred at room temperature for an

additional 2 h, after which the THF was evaporated. The residue was dissolved in ethyl acetate (EtOAc, 50 mL) and the layers were separated. The organic phase was washed with 1M HCl solution (2 × 100 mL), saturated aqueous Na_2CO_3 solution (2 × 100 mL), and brine (100 mL), then dried over Na_2SO_4 and concentrated under reduced pressure. The crude product was purified by silica gel chromatography (DCM: MeOH=30:1) to yield **17** (2.34 g, 69.44% for 2 steps) as a brown solid. ^1H NMR (600 MHz, $CDCl_3$) δ 8.65 (t, *J* = 5.9 Hz, 1H), 7.61 (d, *J* = 1.7 Hz, 1H), 7.61 – 7.60 (m, 2H), 7.60 – 7.50 (m, 2H), 7.49 – 7.46 (m, 1H), 7.46 – 7.45 (m, 2H), 7.44 (dt, *J* = 3.1, 1.5 Hz, 2H), 7.43 – 7.41 (m, 1H), 6.77 (dd, *J* = 15.0, 3.4 Hz, 1H), 6.69, 6.25 (dd, *J* = 15.1, 2.1 Hz, 1H), 4.91 (td, *J* = 9.3, 2.7 Hz, 1H), 4.83 (dt, *J* = 9.6, 3.6 Hz, 1H), 4.57 (d, *J* = 19.2 Hz, 1H), 4.04 (d, *J* = 19.3 Hz, 1H), 3.79 – 3.72 (m, 2H), 3.72 – 3.68 (m, 1H), 3.59 (dd, *J* = 10.0, 6.7 Hz, 1H), 3.47 – 3.40 (m, 1H), 2.85(s, 3H), 2.73 (t, *J* = 2.6 Hz, 1H), 2.14 (td, *J* = 6.9, 2.6 Hz, 2H), 1.69 (dt, *J* = 11.6, 6.2 Hz, 1H), 1.63(s, 3H), 1.53 (dt, *J* = 9.4, 4.8 Hz, 1H), 1.44 (t, *J* = 7.9 Hz, 2H), 1.40 – 1.32 (m, 2H), 1.31 – 1.27 (m, 1H), 1.27 – 1.09 (m, 2H), 1.03 (d, *J* = 6.7 Hz, 3H), 0.99 (s, 9H); ^{13}C NMR (151 MHz, DMSO-d_6) δ 212.2, 173.2, 168.8, 167.7, 166.6, 142.1, 135.0, 132.7, 132.6, 129.8, 129.7, 127.9, 119.9, 84.4, 80.2, 76.7, 71.1, 65.4, 51.1, 50.1, 43.2, 43.0, 34.9, 31.1, 27.9, 27.8, 26.6, 23.9, 20.9, 18.8, 17.6, 15.7; HRMS (ESI) m/z: calcd for $C_{40}H_{54}N_3O_8Si^+$ $[M+H]^+$: 732.3675, found: 732.3670. HPLC purity: 98.31%.

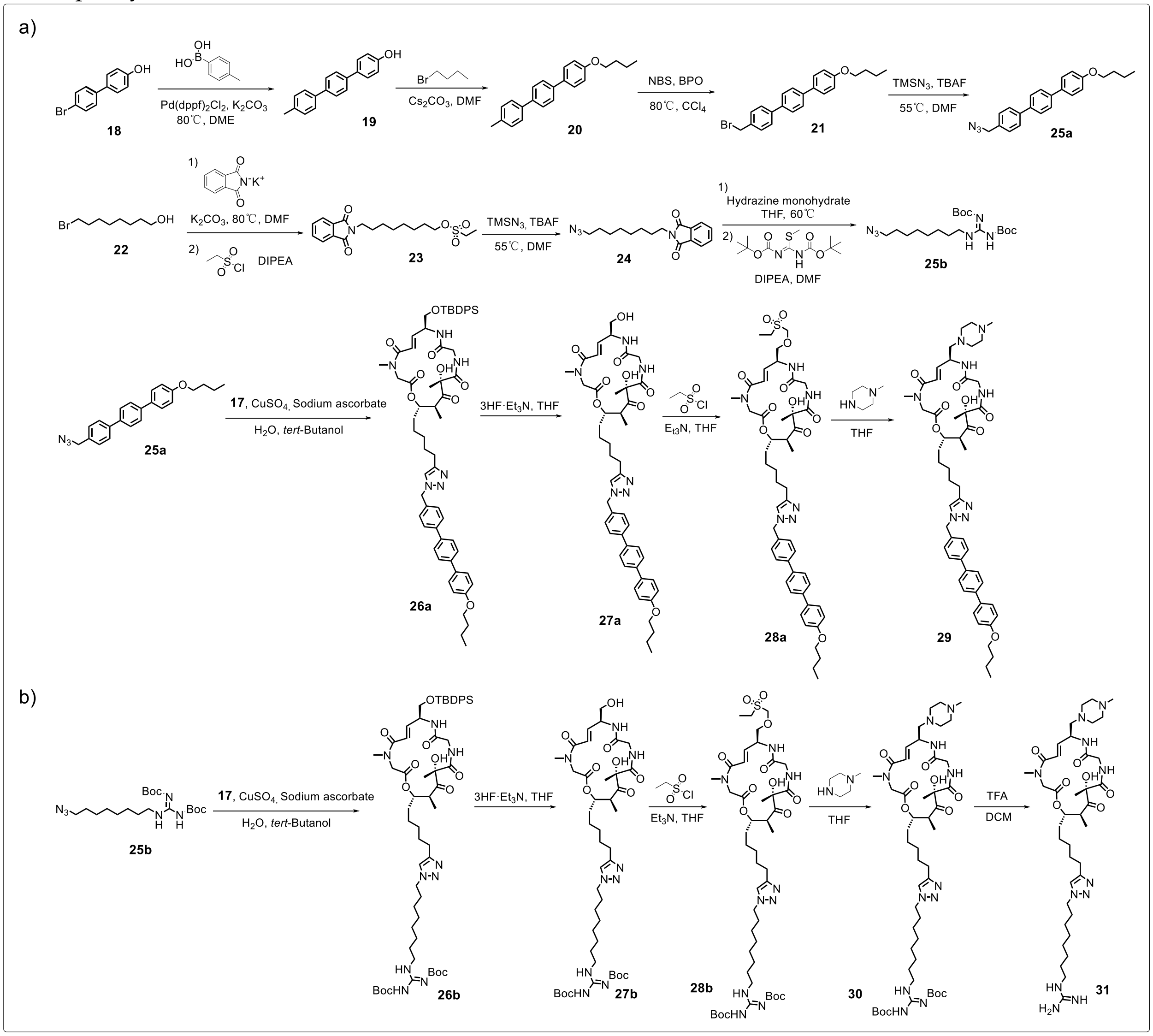

Supplementary Scheme S2. Synthesis of Target Compounds **29** and **31**.

4′′-methyl-[1,1′:4′,1′′-terphenyl]-4-ol (**19**). In a 500.0 mL three-necked reaction flask, compound **18** (10.00 g, 40.14 mmol), 4-methylphenylboronic acid (5.46 g, 40.14 mmol), and Pd(dppf)$_2$Cl$_2$ (2.94 g, 4.01 mmol) were dissolved in 200 mL of DME. Under a nitrogen atmosphere, a saturated potassium carbonate solution (30.0 mL) was added. The reaction mixture was heated to 80°C and stirred for 3 h, then allowed to cool to room temperature overnight while stirring continued. Ethyl acetate (100 mL) was added to the system, stirred, and filtered. The filtrate was washed sequentially with 1M hydrochloric acid, saturated sodium bicarbonate solution, and saturated sodium chloride solution, and then the organic phase was dried over anhydrous sodium sulfate. The solution was filtered and concentrated under reduced pressure to yield an off-white solid. The crude product was purified by silica gel chromatography (PE: EA=1:1) to afford **19** (6.80, 65.13%) as an off-white solid. ^{1}H NMR (600 MHz, DMSO-d_6) δ 9.56 (s, 1H), 7.68 (d, *J* = 8.5 Hz, 2H), 7.65 (d, *J* = 8.5 Hz, 2H), 7.59 (d, *J* = 8.2 Hz, 2H), 7.53 (d, *J* = 8.7 Hz, 2H), 7.28 (d, *J* = 8.2 Hz, 2H), 6.86 (d, *J* = 8.6 Hz, 2H), 2.35 (s, 3H).

4-butoxy-4′′-methyl-1,1′:4′,1′′-terphenyl (**20**). In a 100.0 mL single-necked reaction flask containing **19** (2.40 g, 9.22 mmol), 1-bromobutane (1.40 g, 10.21 mmol), and cesium carbonate (6.50 g, 19.95 mmol), 40.0 mL of DMF was added, and the mixture was stirred at room temperature. After the reaction, 100mL of water and ethyl acetate were added respectively, and the mixture was stirred and allowed to separate. The organic layer was washed sequentially with 1M brine, saturated sodium bicarbonate solution, and saturated sodium chloride solution, then dried over anhydrous sodium sulfate. The solution was concentrated under reduced pressure to obtain **20** (3.00 g, 90.90%) as an off-white solid. ^{1}H NMR (600 MHz, DMSO-d_6) δ 7.69 (d, *J* = 1.9 Hz, 4H), 7.64 (d, *J* = 8.7 Hz, 2H), 7.61 – 7.59 (m, 2H), 7.28 (d, *J* = 7.8 Hz, 2H), 7.03 (d, *J* = 8.8 Hz, 2H), 4.02 (t, *J* = 6.5 Hz, 2H), 2.35 (s, 3H), 1.75 – 1.69 (m, 2H), 1.49 – 1.42 (m, 2H), 0.95 (t, *J* = 7.4 Hz, 3H).

4-(bromomethyl)-4′′-butoxy-1,1′:4′,1′′-terphenyl (**21**). In a 50 mL three-necked reaction flask, compound **20** (0.82 g, 2.59 mmol) and 15.0 mL of carbon tetrachloride were added and stirred until clear. Benzoyl peroxide (BPO, 0.31 g, 1.30 mmol) and *N*-bromosuccinimide (NBS, 0.50 g, 2.85 mmol) were then added, and the reaction mixture was stirred at 80°C for 7 h, followed by cooling to room temperature overnight. A white solid precipitated, which was filtered and air-dried to obtain **21** (0.82 g, 80.39%) as an off-white solid.^{1}H NMR (600 MHz, DMSO-d_6) δ 7.87 (dd, *J* = 30.1, 7.8 Hz, 2H), 7.76 (s, 2H), 7.72 (t, *J* = 10.2 Hz, 2H), 7.67 (d, *J* = 8.4 Hz, 2H), 7.62 – 7.46 (m, 2H), 7.04 (s, 2H), 5.50 (s, 1H), 4.74 (d, *J* = 47.2 Hz, 1H), 4.02 (s, 2H), 1.72 (t, *J* = 7.5 Hz, 2H), 1.49 – 1.41 (m, 2H), 0.98 – 0.89 (m, 3H).

8-(1,3-dioxoisoindolin-2-yl)octyl ethanesulfonate (**23**). Potassium phthalimide (0.88 g, 4.78 mmol) and compound **22** (1.0 g, 4.78 mmol) were added to a 100 mL single-neck flask and dissolved in 20.0 mL of DMF. Solid potassium carbonate (1.32 g, 9.56 mmol) was added in portions, causing the mixture to gradually become cloudy. The reaction mixture was heated to 80 °C and stirred for 6 h. Subsequently, 30 mL of water was added, and the mixture was extracted with ethyl acetate (2 × 30 mL). The organic layers were combined, washed with saturated sodium chloride solution, dried over anhydrous sodium sulfate, filtered, and concentrated under reduced pressure to afford a crude product, which was then dissolved in 20 mL of DMF. The solution was cooled to 10 °C, and DIPEA (1.40 mL, 7.99 mmol) was added, followed by the dropwise addition of ethanesulfonyl chloride (0.77 g, 5.99 mmol). The mixture was stirred at room temperature for 1 h. Subsequently, 20.0 mL of water was added to quench the reaction, and the mixture was extracted with ethyl acetate (2 × 20 mL). The organic layers were combined and washed sequentially with 1 M hydrochloric acid, saturated sodium bicarbonate solution, and saturated sodium chloride solution.

The organic phase was dried over anhydrous sodium sulfate, filtered, and evaporated to dryness to yield a yellow oily crude product **23**, which was used directly in the next step.

2-(8-azidooctyl)isoindoline-1,3-dione (**24**). The crude product of compound **23** (obtained above, dissolved in 15.0 mL of DMF) under a nitrogen atmosphere was added trimethylsilyl azide (0.42 g, 3.67 mmol) and tetrabutylammonium fluoride (0.96 g, 3.67 mmol) successively, and the mixture was heated to 55°C and stirred for 2 h. The reaction was quenched by the addition of 10.0 mL of water, followed by extraction with ethyl acetate (3 × 20 mL). The combined organic phases were washed with 1M hydrochloric acid, saturated sodium bicarbonate solution, and saturated sodium chloride solution, then dried over an appropriate amount of anhydrous sodium sulfate. The solution was filtered and concentrated, and the residue was purified by silica gel chromatography (DCM: MeOH=60:1) to yield **24** (0.50 g, 67.97% for two steps) as an off-white solid. ^{1}H NMR (400 MHz, $CDCl_3$) δ 7.89 – 7.84 (m, 2H), 7.75 (dt, *J* = 5.0, 1.9 Hz, 2H), 3.71 (t, *J* = 7.3 Hz, 2H), 3.28 (t, *J* = 7.0 Hz, 2H), 1.71 (t, *J* = 7.0 Hz, 2H), 1.61 (q, *J* = 6.9 Hz, 2H), 1.37 (d, *J* = 4.6 Hz, 8H).

4-(azidomethyl)-4''-butoxy-1,1':4',1''-terphenyl (**25a**). Compound **21** (0.80 g, 2.03 mmol) was dissolved in 15.0 mL of DMF, forming a colorless reaction solution. Trimethylsilyl azide (0.37 g, 3.04 mmol) and TBAF (0.80 g, 3.04 mmol) were added, and the reaction mixture was stirred at 55°C for 2 h, then allowed to cool to room temperature overnight. A solid precipitated, which was filtered to yield **25a** (0.43 g, 59.31%) as an off-white solid. ^{1}H NMR (600 MHz, $CDCl_3$) δ 7.66 (d, *J* = 1.8 Hz, 1H), 7.66 – 7.60 (m, 5H), 7.57 (d, *J* = 8.7 Hz, 2H), 7.41 (d, *J* = 8.0 Hz, 2H), 7.00 (d, *J* = 8.7 Hz, 2H), 4.40 (s, 2H), 4.02 (t, *J* = 6.5 Hz, 2H), 1.85 – 1.77 (m, 2H), 1.53 (q, *J* = 7.5 Hz, 2H), 1.00 (t, *J* = 7.4 Hz, 3H).

Synthesis of (**25b**). **24** (0.50 g, 1.66 mmol) was dissolved in THF (10.0 mL). Hydrazine monohydrate (243 μL, 4.99 mmol) was added and the reaction mixture was stirred overnight at 60 °C. The solvent was removed in vacuo and chloroform (10 mL) was added. The solution was extracted with NaOH solution (2 × 10 mL, 1 M). The organic fraction was dried with $NaSO_4$, filtered and concentrated in vacuo. Subsequently, 1,3-Di-Boc-2-methylisothiourea (0.38 g, 1.32 mmol), DIPEA (174 μL, 1.76 mmol), and 6.0 mL DMF were added. The reaction mixture was stirred at 0 °C for 2 h, diluted with EtOAc, filtered through a short pad of Celite, and the filtrate was concentrated under reduced pressure. The oily residue was purified by silica gel chromatography (DCM: MeOH=30:1) to yield **25b** (0.22 g, 60.53%) as an off-white solid. ^{1}H NMR (400 MHz, $CDCl_3$) δ 11.49 (s, 1H), 8.29 (s, 1H), 3.39 (td, *J* = 7.3, 5.2 Hz, 2H), 3.24 (t, *J* = 6.9 Hz, 2H), 1.62 – 1.54 (m, 4H), 1.49 (s, 9H), 1.48 (s, 9H), 1.34 – 1.24 (m, 8H); LCMS: retention time 11.57 min, *m/z* = 413.32 [M+H]$^+$.

(8S,14S,16S,17R,E)-17-(5-(1-((4''-butoxy-[1,1':4',1''-terphenyl]-4-yl)methyl)-1H-1,2,3-triazol-4-yl)pentyl)-8-(((tert-butyldiphenylsilyl)oxy)methyl)-14-hydroxy-4,14,16-trimethyl-1-oxa-4,9,12-triazacycloheptadec-6-ene-2,5,10,13,15-pentaone (**26a**). In a 25 mL single-necked reaction flask, compound **25a** (0.43 g, 1.20 mmol) and 17 (0.88 g) were added to 15.0 mL of tert-butanol. Under stirring, 0.36 mL of copper sulfate aqueous solution (1.0 M) and 0.72 mL of sodium ascorbate (1.0 M) were added, and the mixture was stirred at room temperature overnight. A solid precipitated, and an additional 6.0 mL of tetrahydrofuran, 0.36 mL of copper sulfate aqueous solution (1.0 M), and 0.72 mL of sodium ascorbate (1.0 M) were added. The mixture was heated to 55°C and stirred for 8 h. After completion of the reaction, 5 mL of ethyl acetate and saturated sodium chloride solution were added respectively, and the layers were separated. The organic phase was concentrated, and the residue was purified by silica gel chromatography (DCM: MeOH=2:1) to yield **26a** (0.51 g, 78.10%) as an off-white solid; LCMS: retention time 8.62 min, *m/z* = 1090.03 [M+H]$^+$.

(8S,14S,16S,17R,E)-17-(5-(1-((4′′-butoxy-[1,1′:4′,1′′-terphenyl]-4-yl)methyl)-1H-1,2,3-triazol-4-yl)pentyl)-14-hydroxy-8-(hydroxymethyl)-4,14,16-trimethyl-1-oxa-4,9,12-triazacycloheptadec-6-ene-2,5,10,13,15-pentaone (**27a**). To a solution of **26a** (0.51 g，0.47 mmol）in 8.0 mL of THF was added 3HF•Et_3N (0.53 g, 3.28 mmol). The reaction mixture was stirred at room temperature overnight. 3HF·Et_3N (0.30 g, 1.86 mmol) was then added, and the mixture was stirred for an additional 4 h. Following this, 10.0 mL of water and 5.0 mL of tetrahydrofuran were added, and the layers were separated. The aqueous phase was adjusted to a basic *p*H, and the organic compound was extracted with 10.0 mL of tetrahydrofuran. The combined organic layers were dried over anhydrous sodium sulfate and concentrated under reduced pressure. The crude product was purified by silica gel chromatography (DCM: MeOH = 15:1) to obtain **27a** (0.25g, 62.50%) as a white solid. ^{1}H NMR (600 MHz, $CDCl_3$) δ 7.67 – 7.65 (m, 6H), 7.60 – 7.56 (m, 2H), 7.37 (d, *J* = 8.2 Hz, 2H), 7.29 (s, 1H), 7.01 (d, *J* = 8.7 Hz, 2H), 5.56 (d, *J* = 3.2 Hz, 2H), 5.32 (s, 1H), 5.16 (td, *J* = 8.9, 3.2 Hz, 1H), 4.45 (dd, *J* = 16.5, 7.6 Hz, 1H), 4.36 – 4.25 (m, 1H), 4.04 (t, *J* = 6.5 Hz, 2H), 3.92 (dd, *J* = 16.5, 3.6 Hz, 1H), 3.77 – 3.62 (m, 1H), 3.59 – 3.51 (m, 1H), 3.19 (s, 3H), 3.10 – 3.04 (m, 2H), 2.96 (s, 1H), 2.73 – 2.68 (m, 2H), 2.41 – 2.52 (m, 4H), 2.29 (s, 2H), 1.84 – 1.80 (m, 2H), 1.71 (s, 3H), 1.67 (dd, *J* = 14.9, 7.2 Hz, 2H), 1.61 – 1.55 (m, 2H), 1.53 (d, *J* = 7.4 Hz, 1H), 1.42 – 1.31 (m, 4H), 1.17 (dd, *J* = 9.9, 7.0 Hz, 3H), 1.02 (t, *J* = 7.4 Hz, 3H).

((8S,14S,16S,17R,E)-17-(5-(1-((4′′-butoxy-[1,1′:4′,1′′-terphenyl]-4-yl)methyl)-1H-1,2,3-triazol-4-yl)pentyl)-14-hydroxy-4,14,16-trimethyl-2,5,10,13,15-pentaoxo-1-oxa-4,9,12-triazacycloheptadec-6-en-8-yl)methyl ethanesulfonate (**28a**). Compound **27a** (0.20 g, 0.24 mmol) was dissolved in 3.0 mL of tetrahydrofuran, and triethylamine (0.05 g, 0.47 mmol) was added. The mixture was stirred while cooling, and ethylsulfonyl chloride (0.05 g, 0.35 mmol) was added dropwise. The reaction was carried out at 10°C for 2 h. Subsequently, 5.0 mL of water and 5.0 mL of ethyl acetate were added, and the layers were separated. The aqueous phase was extracted with ethyl acetate (2 × 5 mL), and the combined organic phases were washed with saturated brine and dried over anhydrous sodium sulfate. The solvent was removed under reduced pressure, and the crude product **28a** was used in the next step.

(8S,14S,16S,17R,E)-17-(5-(1-((4′′-butoxy-[1,1′:4′,1′′-terphenyl]-4-yl)methyl)-1H-1,2,3-triazol-4-yl)pentyl)-14-hydroxy-4,14,16-trimethyl-8-((4-methylpiperazin-1-yl)methyl)-1-oxa-4,9,12-triazacycloheptadec-6-ene-2,5,10,13,15-pentaone (**29**). The crude product of **28a** (obtained above, dissolved in DMF) was added N-methylpiperazine (0.24 g, 2.35 mmol), and the reaction mixture was stirred at 55°C for 8 h. The solvent was removed under reduced pressure. To the residue, 20 mL of ethyl acetate was added to induce precipitation of a white solid. After filtration, the product was purified by silica gel chromatography to yield **29** (0.03 g, 13.70% for two steps) as an off-white solid. ^{1}H NMR (600 MHz, DMSO-d_6) δ 8.48 (t, *J* = 6.0 Hz, 1H), 8.38 (s, 1H), 7.93 (s, 1H), 7.73 – 7.68 (m, 6H), 7.64 (d, *J* = 8.8 Hz, 2H), 7.38 (d, *J* = 8.3 Hz, 2H), 7.05 – 7.01 (m, 2H), 6.59 (s, 1H), 5.58 (s, 2H), 5.51 (s, 1H), 5.21 – 5.12 (m, 1H), 4.27 (d, *J* = 18.9 Hz, 1H), 4.05 – 4.01 (m, 3H), 3.89 (dd, *J* = 15.9, 6.8 Hz, 1H), 3.58 (dd, *J* = 15.9, 5.2 Hz, 1H), 3.39 (q, *J* = 7.0 Hz, 1H), 3.11 (d, *J* = 13.4 Hz, 1H), 2.92 (dd, *J* = 18.8, 7.2 Hz, 2H), 2.81 (s, 3H), 2.74 (dd, *J* = 18.8, 5.6 Hz, 1H), 2.60 (t, *J* = 7.6 Hz, 2H), 2.39 (s, 4H), 2.35 (s, 2H), 2.23 (s, 3H), 1.75 – 1.69 (m, 2H), 1.65 – 1.51 (m, 4H), 1.49 (s, 3H), 1.47 – 1.42 (m, 2H), 1.42 – 1.34 (m, 1H), 1.30 (dd, *J* = 18.8, 10.0 Hz, 2H), 1.26 – 1.14 (m, 2H), 1.02 (d, *J* = 6.8 Hz, 3H), 0.95 (t, *J* = 7.4 Hz, 3H); ^{13}C NMR (101 MHz, DMSO-d_6) δ 170.9, 167.1, 139.1, 128.5, 127.7, 127.1, 126.9, 126.7, 122.0, 115.0, 80.1, 75.6, 67.2, 52.4, 50.7, 44.2, 43.2, 40.2, 38.9, 34.7, 30.8, 29.1, 28.9, 28.6, 28.5, 25.0, 24.5, 21.4, 18.8, 13.9, 13.8; HRMS (ESI) *m/z*: calcd for $C_{52}H_{69}N_8O_8^+$ [M+H]$^+$: 933.5233, found: 933.5222.

(14S,16S,17S,E)-17-(5-(1-((4′′-butoxy-[1,1′:4′,1′′-terphenyl]-4-yl)methyl)-1H-1,2,3-triazol-4-yl)pentyl)-8-(((tert-butyldiphenylsilyl)oxy)methyl)-14-hydroxy-4,14,16-trimethyl-1-oxa-4,9,12-triazacycloheptadec-6-ene-2,5,10,13,15-pentaone (**26b**). Compounds **25b** (0.22 g, 0.53 mmol) and **17** (0.59 g, 0.80 mmol) were added to a 50 mL

single-necked flask and dissolved in 10.0 mL of tert-butanol. After stirring to achieve a homogeneous yellow solution, water (5.0 mL) was added, resulting in a slightly turbid mixture. Subsequently, 1 M aqueous copper sulfate (107 μL, 0.11 mmol) and 1 M aqueous sodium ascorbate (213 μL, 0.21 mmol) were added sequentially, turning the mixture pale green. The reaction was stirred overnight. To the mixture, 30 mL of water and 50 mL of ethyl acetate were added, and the layers were allowed to separate after stirring. The aqueous phase was extracted with ethyl acetate (2 × 30 mL). The combined organic phases were washed with saturated sodium chloride solution, dried over anhydrous sodium sulfate, filtered, and concentrated under reduced pressure. The crude product was purified by silica gel chromatography (DCM: MeOH = 50:1 to 20:1) to give **26b** (0.66 g, 20%) as an off-white solid. ^{1}H NMR (600 MHz, DMSO-d_6) δ 11.49 (s, 1H), 8.67 (t, *J* = 5.9 Hz, 1H), 8.26 (t, *J* = 5.6 Hz, 1H), 7.81 (s, 1H), 7.64 – 7.59 (m, 5H), 7.59 (s, 1H), 7.46 – 7.43 (m, 5H), 7.43 (s, 1H), 6.77 (dd, *J* = 15.0, 3.4 Hz, 1H), 6.70 (s, 1H), 6.25 (dd, *J* = 15.1, 2.1 Hz, 1H), 4.89 (dd, *J* = 9.2, 2.7 Hz, 1H), 4.82 (dd, *J* = 6.2, 2.6 Hz, 1H), 4.57 (d, *J* = 19.2 Hz, 1H), 4.26 (t, *J* = 7.1 Hz, 2H), 4.06 – 4.03 (m, 1H), 3.75 – 3.72 (m, 2H), 3.71 – 3.69 (m, 1H), 3.60 – 3.57 (m, 1H), 3.44 – 3.40 (m, 1H), 3.23 (s, 2H), 2.85 (d, *J* = 2.1 Hz, 3H), 2.58 (t, *J* = 7.6 Hz, 2H), 2.16 – 2.11 (m, 1H), 1.82 – 1.73 (m, 2H), 1.68 (s, 1H), 1.62 (d, *J* = 2.6 Hz, 3H), 1.59 – 1.54 (m, 2H), 1.47 (s, 9H), 1.44 (s, 1H), 1.38 (s, 9H), 1.24 (d, *J* = 5.3 Hz, 10H), 1.19 (dd, *J* = 13.0, 7.2 Hz, 2H), 1.02 (dd, *J* = 6.8, 4.5 Hz, 3H), 0.98 (s, 9H).

synthesis of **28b**. **26b** (0.66 g, 0.58 mmol) was added to a 50 mL single-necked flask and dissolved in THF (10.0 mL). Under a nitrogen atmosphere, 3HF·Et_3N (0.46 g, 2.85 mmol) was added dropwise, and the mixture was stirred overnight. Then, 3.5 mL of water was added, and the mixture was stirred and extracted with ethyl acetate (3 × 10 mL). The combined organic phases were washed with saturated sodium bicarbonate solution and saturated sodium chloride solution, then dried over anhydrous sodium sulfate. After filtration and concentration under reduced pressure, the crude product was purified by silica gel chromatography (DCM: MeOH = 30:1 to 15:1) to obtain **27b** (450.0 mg, 74.88%) as a white solid. Compound **27b** (0.25 g, 0.28 mmol) was immediately added to a 50 mL single-necked flask and dissolved in THF (2.5 mL), forming a clear, colorless solution. Triethylamine (54.98 mg, 0.54mmol) was added dropwise to the mixture, followed by the dropwise addition of ethylsulfonyl chloride (52.40 mg, 0.41 mmol). The reaction mixture was stirred for 2.5 h. To quench the reaction, 2 mL of water was added, followed by extraction with 20 mL of ethyl acetate. Sodium chloride was added to the aqueous phase, which was then extracted with a small amount of ethyl acetate. The combined organic phases were washed with dilute hydrochloric acid, saturated sodium carbonate solution, and saturated sodium chloride solution, then dried over anhydrous sodium sulfate. After filtration and concentration under reduced pressure, the crude product **28b** was obtained and used directly in the next step; LCMS: retention time 6.97 min, *m*/*z* = 998.62 $[M+H]^+$.

synthesis of **30**. The crude product **28b** (0.28g, 0.28 mmol) was added to a 50 mL single-necked flask and dissolved in THF (3.0 mL), forming a clear, colorless solution. *N*-methylpiperazine (0.14g, 1.40 mmol) was added dropwise, and the mixture was stirred overnight under a nitrogen atmosphere. The reaction mixture was concentrated to remove the solvent and directly purified by silica gel chromatography (DCM: MeOH = 50:1 to 10:1) to give **30** (0.22g, 77.39%) as an off-white solid. ^{1}H NMR (400 MHz, DMSO-d_6) δ 11.48 (s, 1H), 8.52 (t, *J* = 6.0 Hz, 1H), 8.41 (s, 1H), 8.26 (t, *J* = 5.6 Hz, 1H), 7.82 (s, 1H), 6.64 (s, 1H), 5.51 (t, *J* = 5.9 Hz, 1H), 5.18 – 5.10 (m, 1H), 4.25 (d, *J* = 7.1 Hz, 2H), 4.03 (d, *J* = 18.9 Hz, 1H), 3.89 – 3.84 (m, 1H), 3.59 (d, *J* = 10.6 Hz, 2H), 3.25 – 3.21 (m, 2H), 3.14 (d, *J* = 13.4 Hz, 1H), 3.05 – 3.01 (m, 1H), 2.97 – 2.90 (m, 2H), 2.80 (s, 3H), 2.73 (d, *J* = 13.2 Hz, 1H), 2.55 (d, *J* = 7.7 Hz, 2H), 2.45 (d, *J* = 7.4 Hz, 2H), 2.40 (s, 2H), 2.28 (s, 3H), 1.76 (t, *J* = 7.2 Hz, 2H), 1.58 (d, *J* = 12.3 Hz, 2H), 1.49 (s, 3H), 1.46 (s, 9H), 1.37 (s, 9H), 1.26 – 1.21 (m, 10H), 1.11 – 1.09 (m, 8H), 1.08 (s, 2H), 1.02 (d, *J* = 6.8 Hz, 3H); ^{13}C NMR (101 MHz, DMSO-d_6) δ 209.8, 173.0, 171.4, 169.3, 167.0, 163.4, 155.5, 152.4, 147.0, 132.0, 121.8, 83.1, 80.3, 78.3, 75.8, 67.2, 61.4, 54.4, 51.7, 51.5, 50.9, 49.4, 45.7, 45.4,

45.1, 44.4, 43.4, 43.1, 34.9, 31.6, 31.0, 30.2, 30.0, 29.1, 29.1, 28.7, 28.7, 28.5, 28.2, 27.9, 26.4, 26.1, 25.2, 24.7, 21.6, 14.0, 10.1; HRMS (ESI) *m/z*: calcd for $C_{48}H_{82}N_{11}O_{11}^{+}$ [M+H]+ : 988.6190, found: 988.6184.

synthesis of **31**. **30** (0.22g, 0.22 mmol) was added to a 50 mL single-necked flask and dissolved in DCM (6.0 mL), forming a clear solution. TFA (2.0 mL) was added dropwise, and the mixture was stirred overnight. Then, 8.0 mL of toluene was added, and the TFA was removed by rotary evaporation. The concentrated mixture was subjected to preparative chromatography. After evaporating the acetonitrile, the product was freeze-dried. This process to give **31** (67.00 mg, 39.18%) as a white solid. ^{1}H NMR (600 MHz, DMSO-*d*6) δ 8.50 (d, *J* = 6.0 Hz, 2H), 7.82 (s, 1H), 7.50 (s, 1H), 6.64 (s, 1H), 5.69 (s, 1H), 5.16 – 5.13 (m, 1H), 4.31 – 4.25 (m, 4H), 4.06 (d, *J* = 18.9 Hz, 1H), 3.88 (dd, *J* = 15.8, 6.5 Hz, 1H), 3.63 – 3.60 (m, 1H), 3.41 – 3.37 (m, 2H), 3.08 (d, *J* = 7.0 Hz, 2H), 3.06 – 3.04 (m, 2H), 2.99 (dd, *J* = 19.1, 6.4 Hz, 2H), 2.82 (s, 3H), 2.75 (d, *J* = 5.4 Hz, 1H), 2.57 (t, *J* = 7.6 Hz, 3H), 1.79 – 1.76 (m, 2H), 1.57 (dt, *J* = 12.4, 6.1 Hz, 4H), 1.53 (d, *J* = 5.5 Hz, 1H), 1.50 (s, 3H), 1.45 (d, *J* = 6.8 Hz, 2H), 1.38 (s, 1H), 1.33 (s, 2H), 1.29 (s, 2H), 1.27 (s, 2H), 1.26 – 1.23 (m, 6H), 1.22 – 1.19 (m, 2H), 1.03 (d, *J* = 6.9 Hz, 3H); ^{13}C NMR (101 MHz, DMSO-*d*6) δ 209.6, 172.9, 170.9, 169.0, 167.0, 156.9, 146.8, 121.6, 118.5, 115.5, 80.1, 75.6, 52.2, 50.6, 49.1, 48.9, 44.1, 43.2, 42.2, 40.7, 34.6, 31.3, 30.8, 30.0, 29.7, 28.9, 28.4, 28.4, 28.3, 25.9, 25.8, 25.0, 24.5, 21.3, 13.9.; HRMS (ESI) *m/z*: calcd for $C_{38}H_{66}N_{11}O_{7}^{+}$ [M+H]+ : 788.5141, found: 788.5137; HPLC purity: 99.42%.

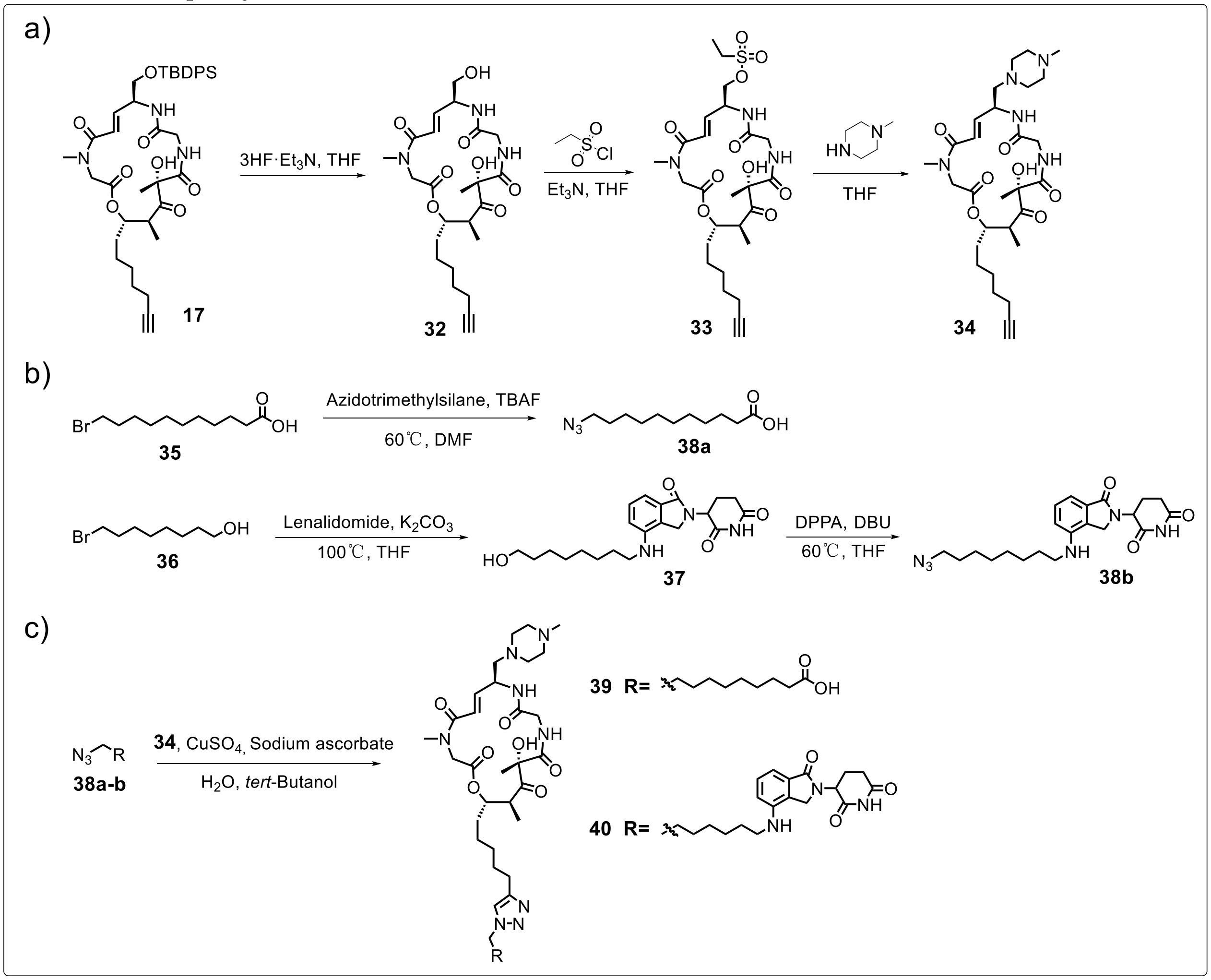

Supplementary Scheme S3. Synthesis of Target Compounds **39** and **40**.

(8S,14S,16S,17S,E)-17-(hept-6-yn-1-yl)-14-hydroxy-8-(hydroxymethyl)-4,14,16-trimethyl-1-oxa-4,9,12-triazacycloheptadec-6-ene-2,5,10,13,15-pentaone (**32**). **17** (1.20 g, 1.64 mmol) was added to a 50 mL single-necked flask and dissolved in THF (20.0 mL) under a nitrogen atmosphere. A solution of triethylamine trihydrofluoride (1.32 g, 8.20 mmol) was added dropwise, and the mixture was stirred overnight. Subsequently, 30 mL of water and 50 mL of ethyl acetate were added, and the mixture was stirred and allowed to separate. The aqueous phase was extracted with ethyl acetate (3 × 30 mL). The combined organic phases were washed with 50.0 mL of saturated sodium bicarbonate solution and 50 mL of saturated sodium chloride solution, then dried over anhydrous sodium sulfate. After filtration and concentration under reduced pressure, the crude product was purified by silica gel chromatography (DCM: MeOH = 10:1) to yield **32** (0.83 g, 64.61%) as an off-white solid. ^{1}H NMR (600 MHz, DMSO-d_6) δ 8.67 (t, *J* = 6.0 Hz, 1H), 7.40 (d, *J* = 9.3 Hz, 1H), 6.75 (s, 1H), 6.17 (dd, *J* = 15.1, 2.0 Hz, 1H), 5.00 (t, *J* = 5.6 Hz, 1H), 4.94 (td, *J* = 9.3, 2.7 Hz, 1H), 4.58 (d, *J* = 19.2 Hz, 2H), 4.03 (d, *J* = 19.3 Hz, 1H), 3.76 (dd, *J* = 16.7, 5.7 Hz, 1H), 3.63 (dd, *J* = 16.7, 6.3 Hz, 1H), 3.49 – 3.44 (m, 2H), 3.37 – 3.33 (m, 1H), 2.84 (s, 3H), 2.74 (t, *J* = 2.6 Hz, 1H), 2.14 (dt, *J* = 7.0, 3.6 Hz, 2H), 1.72 – 1.66 (m, 1H), 1.63 (s, 3H), 1.53 (t, *J* = 4.8 Hz, 1H), 1.46 – 1.37 (m, 4H), 1.34 – 1.28 (m, 2H), 1.24 (dd, *J* = 6.2, 3.2 Hz, 1H), 1.11 (d, *J* = 6.8 Hz, 3H).

((8S,14S,16S,17S,E)-17-(hept-6-yn-1-yl)-14-hydroxy-4,14,16-trimethyl-2,5,10,13,15-pentaoxo-1-oxa-4,9,12-triazacycloheptadec-6-en-8-yl)methyl ethanesulfonate (**33**). **32** (0.83 g, 1.68 mmol) was added to a 50 mL single-necked flask and dissolved in THF (8.0 mL), forming a clear, colorless solution. Triethylamine (0.34 g, 3.35 mmol) was added dropwise, followed by the dropwise addition of ethylsulfonyl chloride (0.32 g, 2.51 mmol). The reaction mixture was stirred for 1 h. To quench the reaction, 5 mL of water was added, followed by extraction with ethyl acetate (2 × 10 mL). Sodium chloride was added to the aqueous phase, which was then extracted with a small amount of ethyl acetate. The combined organic phases were washed with dilute hydrochloric acid, saturated sodium carbonate solution, and saturated sodium chloride solution, then dried over anhydrous sodium sulfate. After filtration and concentration under reduced pressure, 1.13 g of the crude product **33** as a brown solid was obtained and used directly in the next step.

(8S,14S,16S,17S,E)-17-(hept-6-yn-1-yl)-14-hydroxy-4,14,16-trimethyl-8-((4-methylpiperazin-1-yl)methyl)-1-oxa-4,9,12-triazacycloheptadec-6-ene-2,5,10,13,15-pentaone (**34**). The crude product **33** (1.13 g, 1.93 mmol) was added to a 50 mL single-necked flask and dissolved in THF (15.0 mL). *N*-methylpiperazine (0.97 g, 9.65 mmol) was added dropwise, and the mixture was stirred overnight under a nitrogen atmosphere. The reaction mixture was concentrated to remove the solvent and redissolved in DCM. The product was purified by silica gel chromatography (DCM: MeOH = 30:1 to 10:1) to obtain **34** (0.30 g, 27.03%) as a brown solid; HRMS (ESI) m/z: calcd for $C_{29}H_{46}N_5O_7^+$ [M+H]$^+$: 576.3392, found: 576.3400.

3-(4-((8-hydroxyoctyl)amino)-1-oxoisoindolin-2-yl)piperidine-2,6-dione (**37**). **36** (1.00 g, 4.78 mmol) and lenalidomide (1.24 g, 4.78 mmol) were added to a 50 mL single-necked flask and dissolved in DMF (5.0 mL). Potassium carbonate (1.32 g, 9.56 mmol) was gradually added in portions. The reaction mixture was heated to 100°C and stirred for 4 h. After adding 5.0 mL of water, the mixture was extracted with ethyl acetate (2 × 20 mL). The combined organic layers were washed with saturated sodium chloride solution, dried over anhydrous sodium sulfate, filtered, and concentrated under reduced pressure. The crude product was purified by silica gel chromatography (DCM: MeOH = 30:1) to give **37** (1.11 g, 59.46%) as an off-white solid; ^{1}H NMR (400 MHz, DMSO-d_6) δ 7.19 (t, *J* = 7.7 Hz, 1H), 6.92 (dd, *J* = 7.5, 0.8 Hz, 1H), 6.80 (dd, *J* = 7.9, 0.9 Hz, 1H), 5.43 (s, 1H), 5.17 (dd, *J* = 13.4, 5.1 Hz, 1H), 4.21 (d, *J* = 16.9 Hz, 1H), 4.07 (d, *J* = 16.9 Hz, 1H), 3.67 – 3.57 (m, 2H), 3.06 – 2.97 (m, 1H), 2.79 – 2.73 (m, 1H), 2.33 – 2.24 (m, 1H), 2.07 – 2.00 (m, 1H), 1.44 – 1.37 (m, 4H), 1.25 (d, *J* = 5.9 Hz, 8H); ^{13}C NMR (101 MHz, DMSO-d_6) δ 172.2, 171.1, 169.4, 144.1, 132.7,

129.3, 126.1, 116.9, 110.9, 61.2, 52.6, 46.0, 33.0, 31.9, 29.3, 29.2, 27.9, 26.8, 25.9, 22.6; HRMS (ESI) m/z: calcd for $C_{21}H_{30}N_3O_4^+$ [M+H]$^+$: 388.2231, found: 388.2231.

11-azidoundecanoic acid (**38a**). **35** (5.00 g, 18.85 mmol) was added to a 250 mL single-necked flask and dissolved in DMF (50.0 mL). At room temperature, trimethylsilyl azide (3.43 g, 28.28 mmol) and TBAF (8.70 g, 28.28 mmol) were added sequentially. The mixture was heated to 60°C and stirred for 2 h. Following this, 50 mL of water was added, and the mixture was stirred and extracted with ethyl acetate (2 × 50 mL). The combined organic layers were washed with 50 mL of saturated sodium bicarbonate solution and 50 mL of saturated sodium chloride solution, then dried over anhydrous sodium sulfate. After filtration and evaporation to dryness, the product was purified by silica gel chromatography (DCM: MeOH = 20:1) to afford **38a** (2.80 g, 65.42%) as a colorless, transparent liquid. ^{1}H NMR (600 MHz, $CDCl_3$) δ 3.25 (t, *J* = 7.0 Hz, 2H), 2.35 (t, *J* = 7.5 Hz, 2H), 1.65 – 1.56 (m, 4H), 1.41 – 1.32 (m, 4H), 1.31 – 1.22 (m, 8H).

3-(4-((8-azidooctyl)amino)-1-oxoisoindolin-2-yl)piperidine-2,6-dione (**38b**). Compound **37** (0.41 g, 1.06 mmol) and DPPA (0.35 g, 1.27 mmol) were added to a 50.0 mL single-necked flask and dissolved in THF (6.0 mL) under nitrogen gas protection. The mixture was cooled to 0°C, and DBU (0.19 g, 1.27 mmol) was injected. The reaction was then heated to 60°C and stirred for 8 h. Following this, 10 mL of ethyl acetate was added, and the mixture was washed sequentially with 1 M dilute hydrochloric acid, saturated sodium carbonate solution, and saturated sodium chloride solution. The organic layer was dried over anhydrous sodium sulfate, filtered, and concentrated under reduced pressure. The crude product **38b** was used directly in the next step; LCMS: retention time 5.54 min, *m/z* =413.34 [M+H]$^+$.

10-(4-(5-((8S,14S,16S,17S,E)-14-hydroxy-4,14,16-trimethyl-8-((4-methylpiperazin-1-yl)methyl)-2,5,10,13,15-pentaoxo-1-oxa-4,9,12-triazacycloheptadec-6-en-17-yl)pentyl)-1H-1,2,3-triazol-1-yl)decanoic acid (**39**). **34** (80.0 mg, 0.14 mmol) and compound **38a** (0.06 g, 0.28 mmol) were added to a 10 mL single-necked flask with tert-butanol (3.5 mL) and water (1.8 mL) to form a clear solution. A 1 M copper sulfate (42 μL, 0.04 mmol) and a 1 M sodium ascorbate (83 μL, 0.08 mmol) were added dropwise, and the mixture was stirred overnight under a nitrogen atmosphere. The reaction mixture was concentrated to remove the solvent and purified by prep-HPLC to give **39** (8.50 mg, 7.7%) as an off-white solid; ^{1}H NMR (400 MHz, DMSO-d_6) δ 8.50 (t, *J* = 6.0 Hz, 1H), 7.82 (s, 1H), 7.45 – 7.22 (m, 1H), 6.65 (s, 1H), 5.58 (s, 1H), 5.17 – 5.11 (m, 1H), 4.29 (d, *J* = 12.3 Hz, 1H), 4.25 (d, *J* = 7.2 Hz, 2H), 4.05 (d, *J* = 18.8 Hz, 1H), 3.91 – 3.81 (m, 2H), 3.62 – 3.56 (m, 2H), 3.08 (s, 1H), 2.98 (d, *J* = 10.9 Hz, 2H), 2.92 (d, *J* = 6.4 Hz, 2H), 2.81 (s, 3H), 2.77 (s, 2H), 2.67 (q, *J* = 1.9 Hz, 2H), 2.34 – 2.31 (m, 2H), 2.16 (d, *J* = 7.3 Hz, 2H), 2.03 – 1.88 (m, 2H), 1.76 (t, *J* = 7.2 Hz, 2H), 1.49 (s, 3H), 1.36 (d, *J* = 15.6 Hz, 2H), 1.27 (d, *J* = 15.8 Hz, 6H), 1.22 (d, *J* = 4.7 Hz, 10H), 1.20 – 1.15 (m, 4H), 1.10 (s, 2H), 1.02 (d, *J* = 6.9 Hz, 3H); HRMS (ESI) *m/z*: calcd for $C_{40}H_{67}N_8O_9^+$ [M+H]$^+$: 803.5026, found: 803.5032; HPLC purity: 98.36%.

(8S,14S,16S,17S,E)-17-(5-(1-(7-((2-(2,6-dioxopiperidin-3-yl)-1-oxoisoindolin-4-yl)amino)heptyl)-1H-1,2,3-triazol-4-yl)pentyl)-14-hydroxy-4,14,16-trimethyl-8-((4-methylpiperazin-1-yl)methyl)-1-oxa-4,9,12-triazacycloheptadec-6-ene-2,5,10,13,15-pentaone (**40**). **34** (80.0 mg, 0.14 mmol) and compound **38b** (crude product obtained as described above) were added to a 10.0 mL single-necked flask along with tert-butanol (27.0 mL) and water (14.0 mL). A 1 M copper sulfate (328 μL, 0.33 mmol) and a 1 M sodium ascorbate (656 μL, 0.66 mmol) were added dropwise, and the mixture was stirred overnight under a nitrogen atmosphere. The reaction mixture was concentrated to remove the solvent and directly subjected to preparative chromatography, yielding **40** (22.0 mg, 14.5%) as an off-white solid; ^{1}H NMR (600 MHz, DMSO-d_6) δ 8.56 (s, 1H), 8.52 (t, *J* = 6.0 Hz, 1H), 7.81 (s, 1H), 7.20 (t, *J* = 7.6 Hz, 1H), 6.94 – 6.92 (m, 1H), 6.82 (dd, *J* = 7.9, 0.9 Hz, 1H), 5.79 – 5.75 (m, 1H), 5.17 – 5.15 (m, 1H), 5.14 (d, *J* = 3.1 Hz, 1H), 5.13 – 5.10 (m, 1H), 4.29 (s, 1H), 4.27 – 4.26 (m, 2H), 4.24 (s, 1H), 4.22

(d, *J* = 16.9 Hz, 2H), 4.09 – 4.04 (m, 2H), 3.90 – 3.87 (m, 1H), 3.66 – 3.62 (m, 2H), 3.61 – 3.57 (m, 2H), 3.38 (q, *J* = 7.1 Hz, 2H), 3.05 – 3.00 (m, 2H), 2.99 – 2.97 (m, 1H), 2.82 (s, 3H), 2.80 – 2.77 (m, 4H), 2.76 (d, *J* = 6.2 Hz, 1H), 2.74 (dd, *J* = 4.4, 2.4 Hz, 1H), 2.56 (t, *J* = 7.6 Hz, 3H), 2.28 (dd, *J* = 13.2, 4.5 Hz, 1H), 2.06 – 2.03 (m, 1H), 1.78 (d, *J* = 7.2 Hz, 1H), 1.76 – 1.72 (m, 2H), 1.62 – 1.56 (m, 2H), 1.54 (d, *J* = 12.8 Hz, 2H), 1.50 (s, 3H), 1.42 – 1.39 (m, 2H), 1.33 – 1.28 (m, 2H), 1.26 – 1.18 (m, 12H), 1.03 (d, *J* = 6.9 Hz, 3H); ^{13}C NMR (101 MHz, DMSO-d_6) δ 209.9, 173.1, 172.0, 171.2, 170.8, 169.3, 169.2, 167.1, 147.0, 143.9, 134.3, 132.5, 129.9, 129.1, 125.9, 121.9, 116.7, 110.7, 80.3, 75.8, 60.5, 52.9, 52.9, 52.4, 51.4, 50.8, 49.3, 45.9, 44.4, 43.4, 42.6, 34.9, 31.5, 31.0, 30.2, 29.9, 29.3, 29.1, 28.7, 28.5, 27.6, 26.5, 26.0, 25.2, 24.7, 22.4, 21.6, 14.1; HRMS (ESI) *m*/*z*: calcd for $C_{50}H_{74}N_{11}O_{10}^+$ $[M+H]^+$: 988.5615, found: 988.5595; HPLC purity: 98.36%.

9. NMR Spectra

^{1}H NMR (600 MHz, $CDCl_3$) of **2**

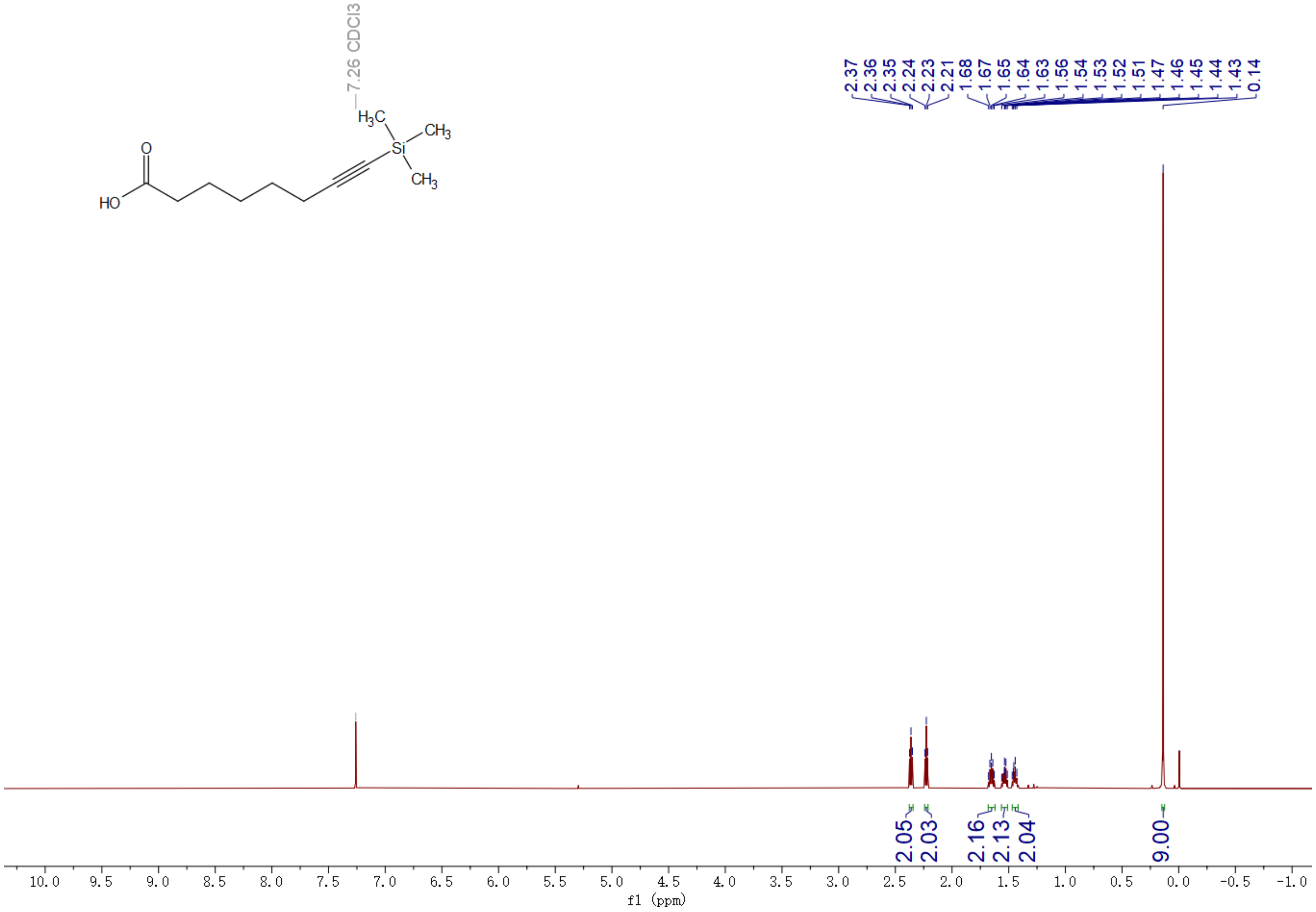

^{13}C NMR (101 MHz, DMSO-*d*$_6$) of **2**

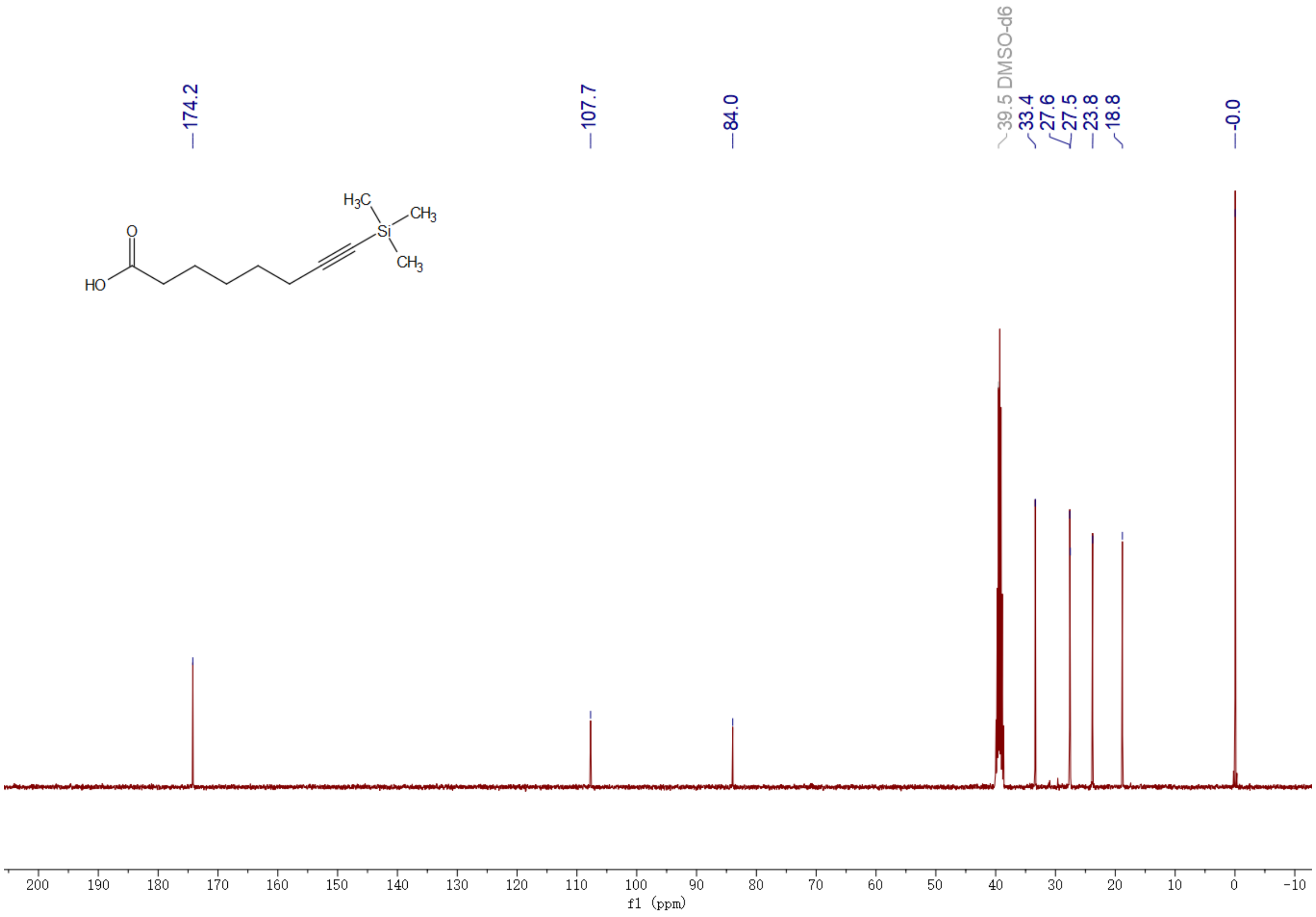

^{1}H NMR (600 MHz, $CDCl_3$) of **3**

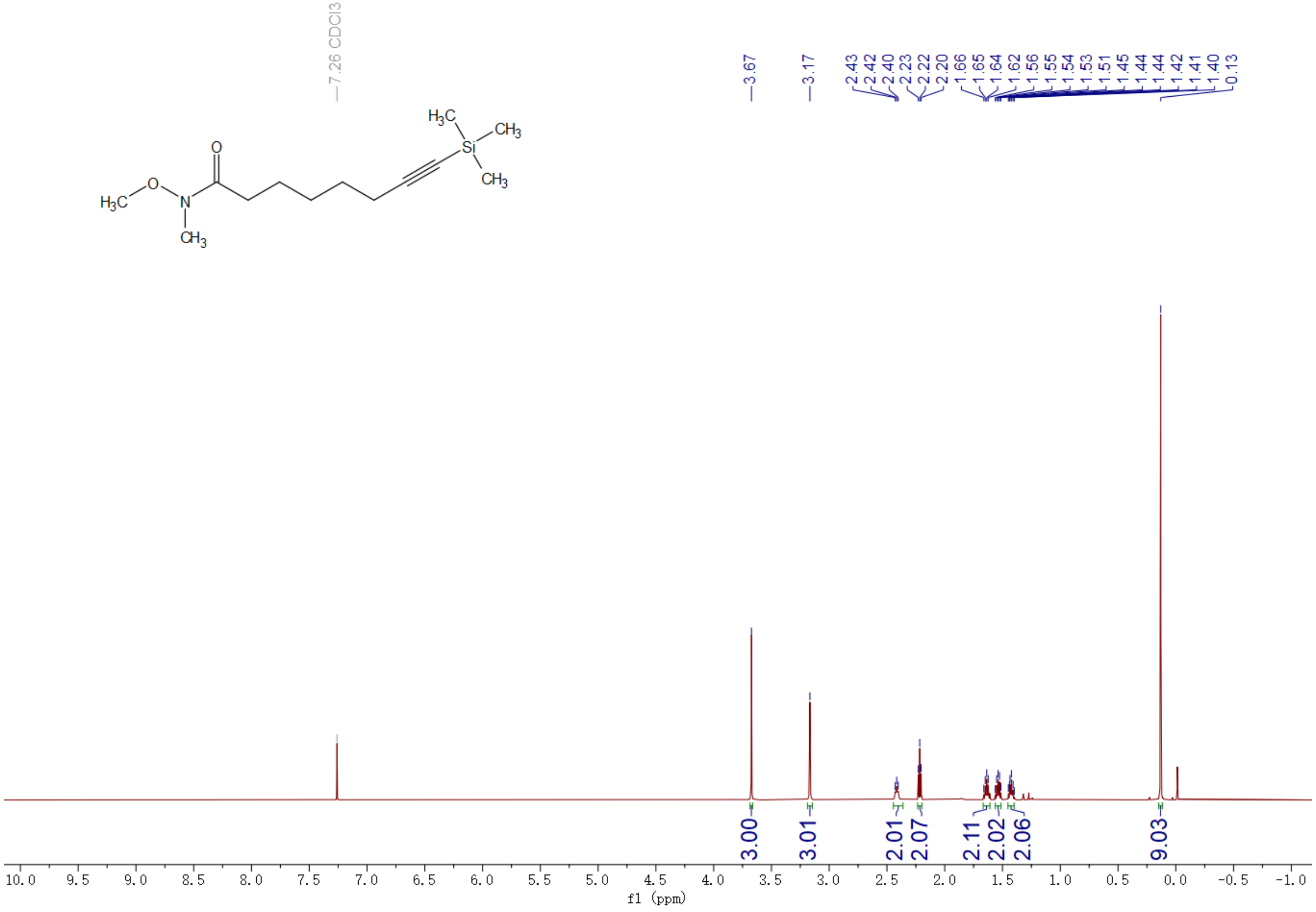

^{1}H NMR (600 MHz, $CDCl_3$) of **4**

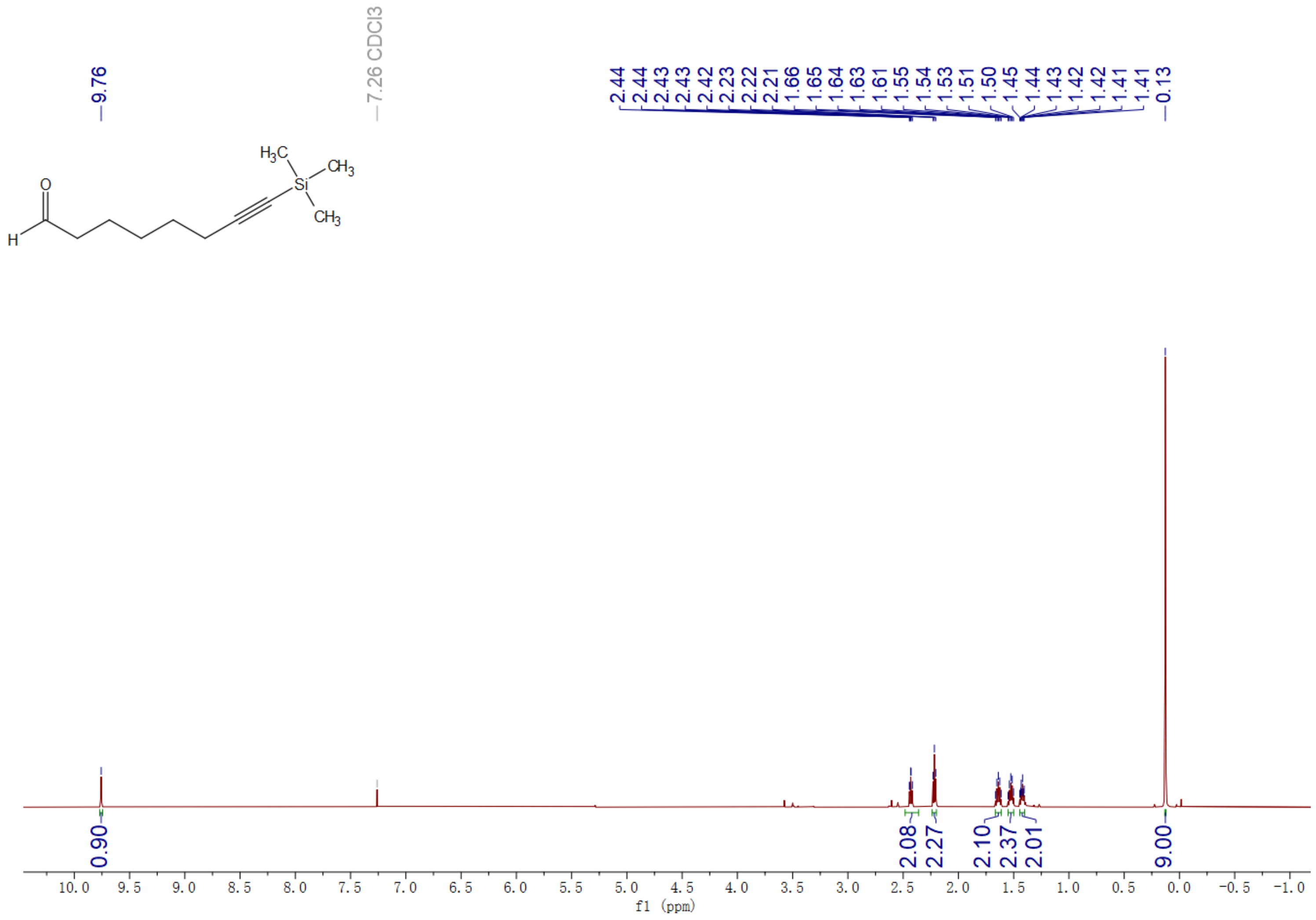

^{1}H NMR (600 MHz, $CDCl_3$) of **6**

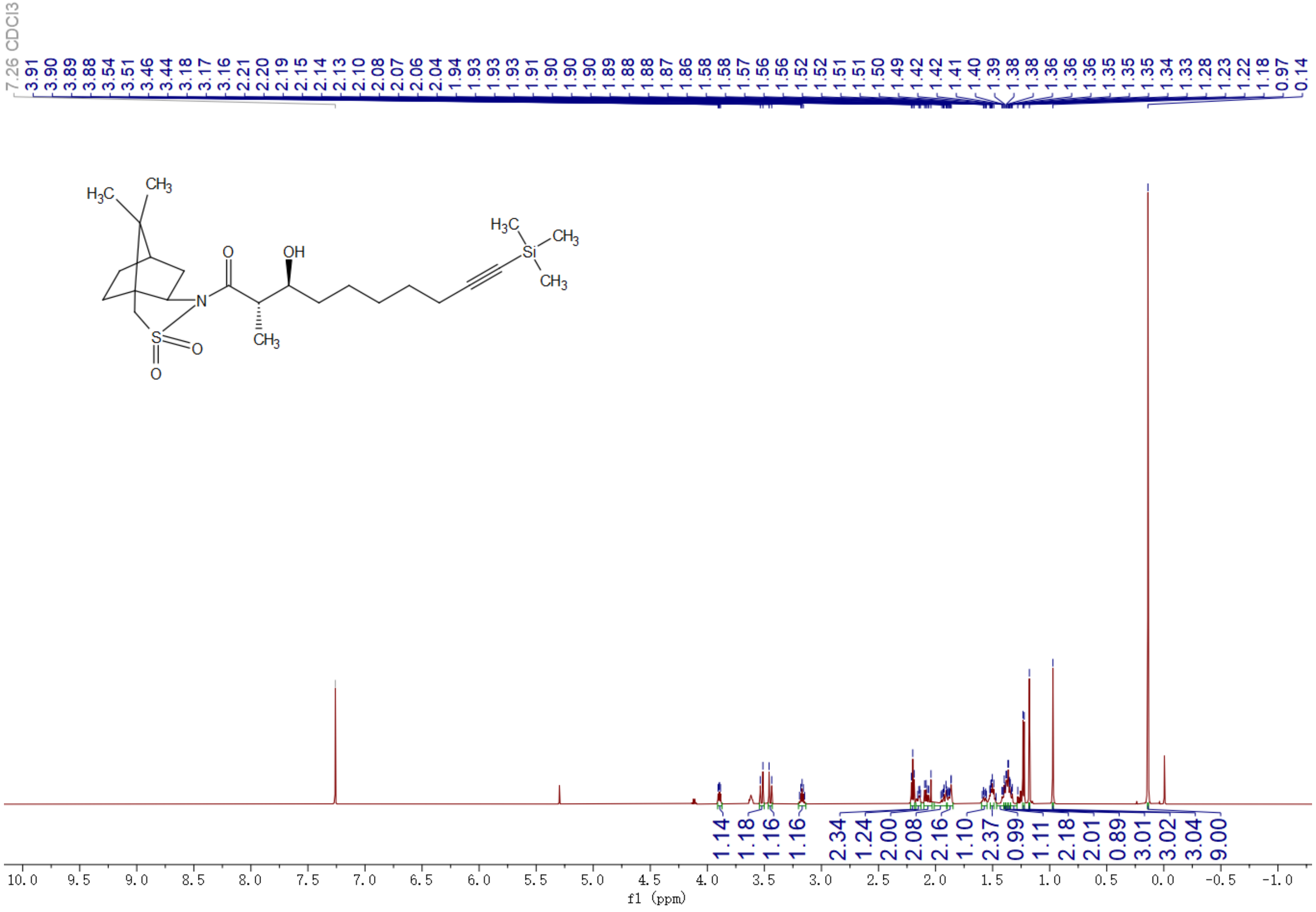

^{13}C NMR (101 MHz, DMSO-*d*6) of **6**

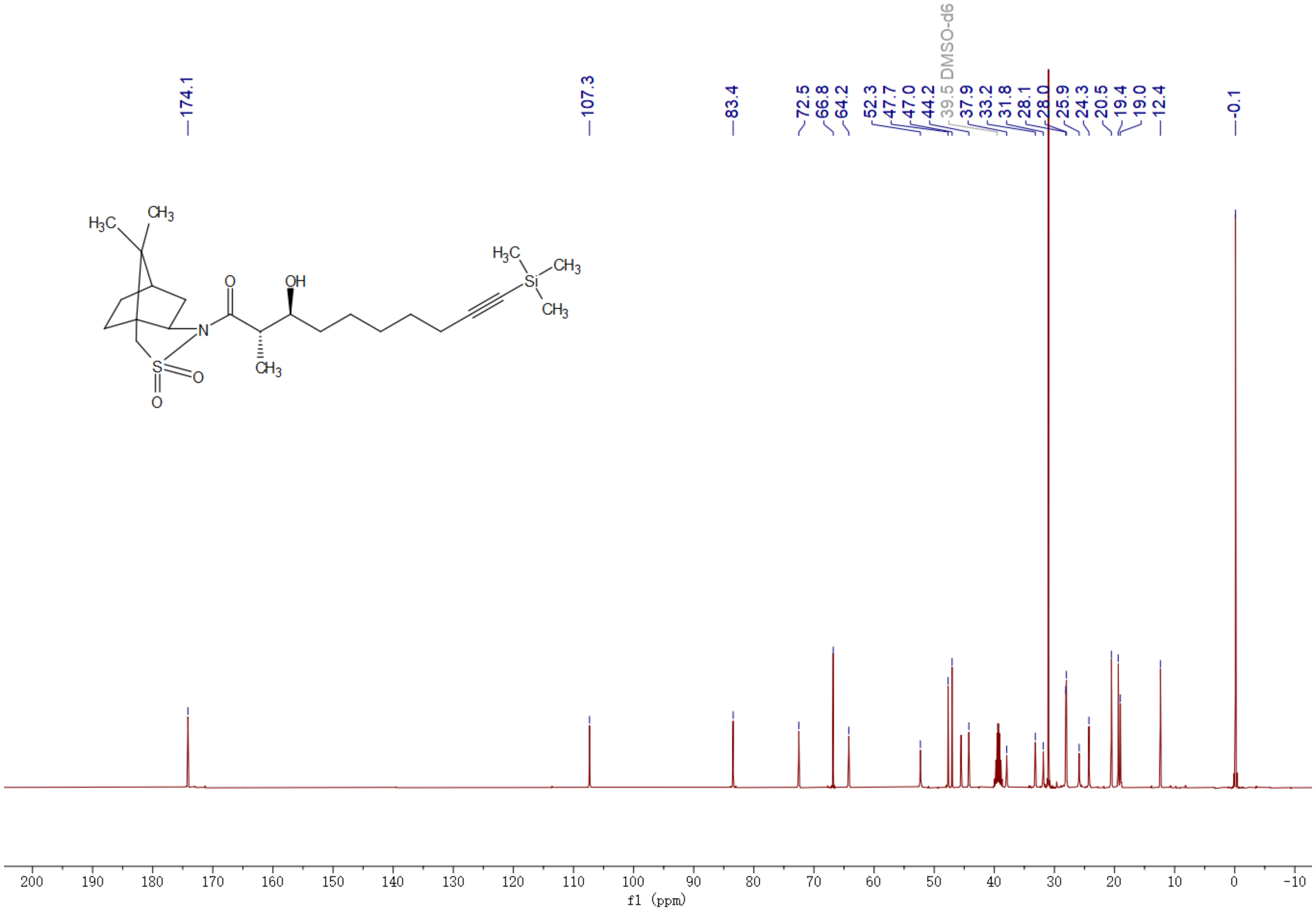

^{1}H NMR (600 MHz, $CDCl_3$) of **7**

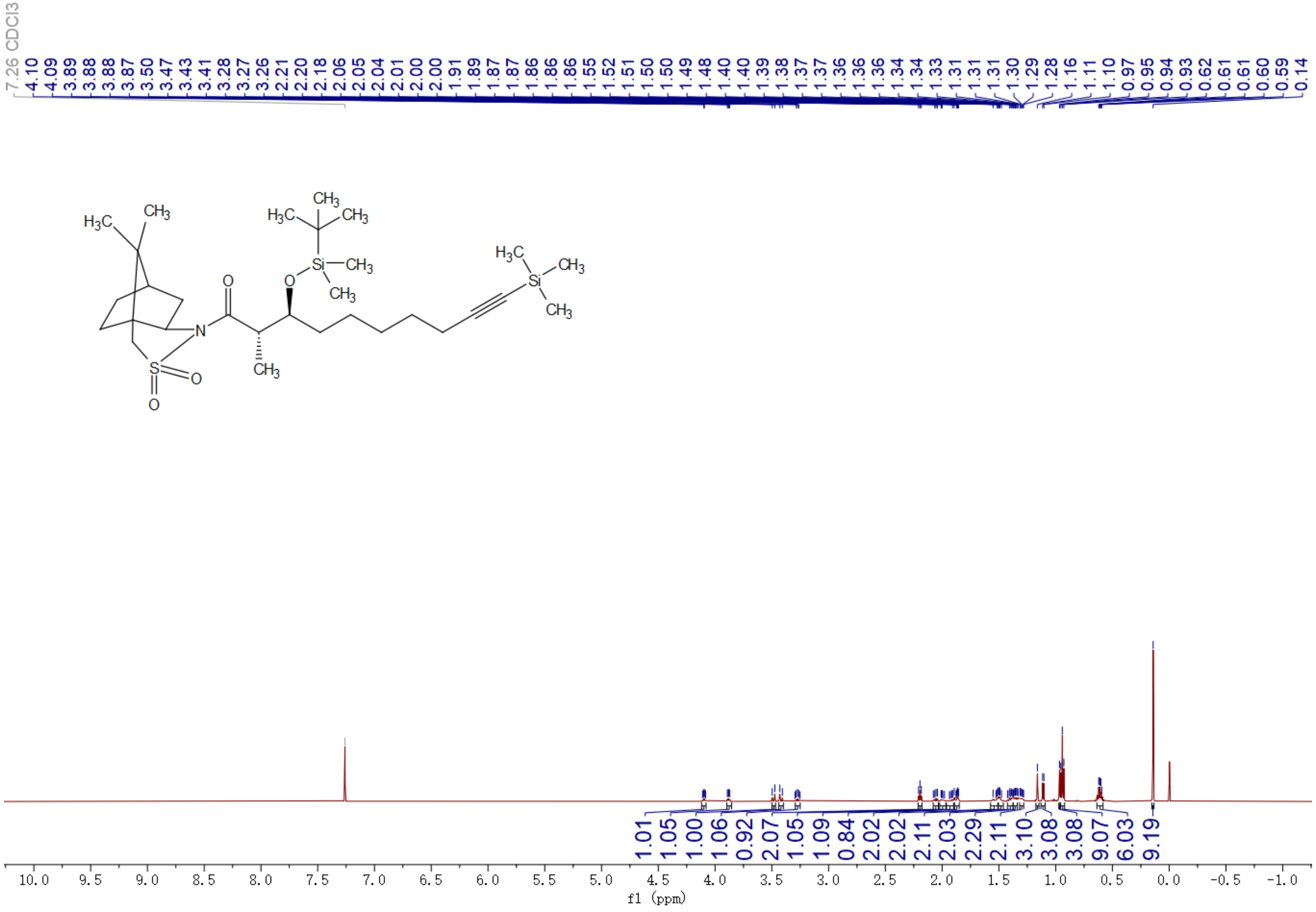

^{1}H NMR (600 MHz, $CDCl_3$) of **8**

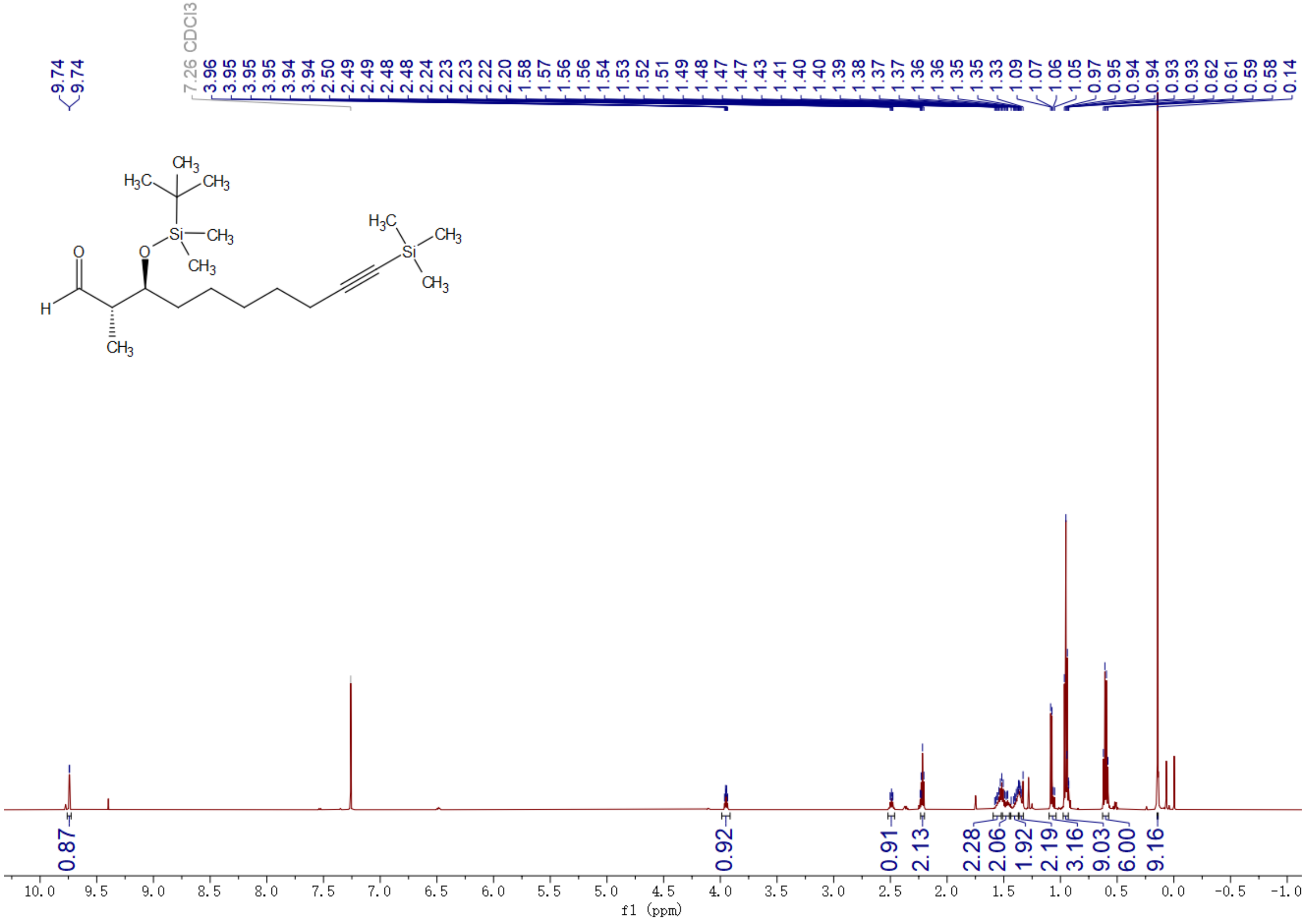

^1H NMR (600 MHz, $CDCl_3$) of **9**

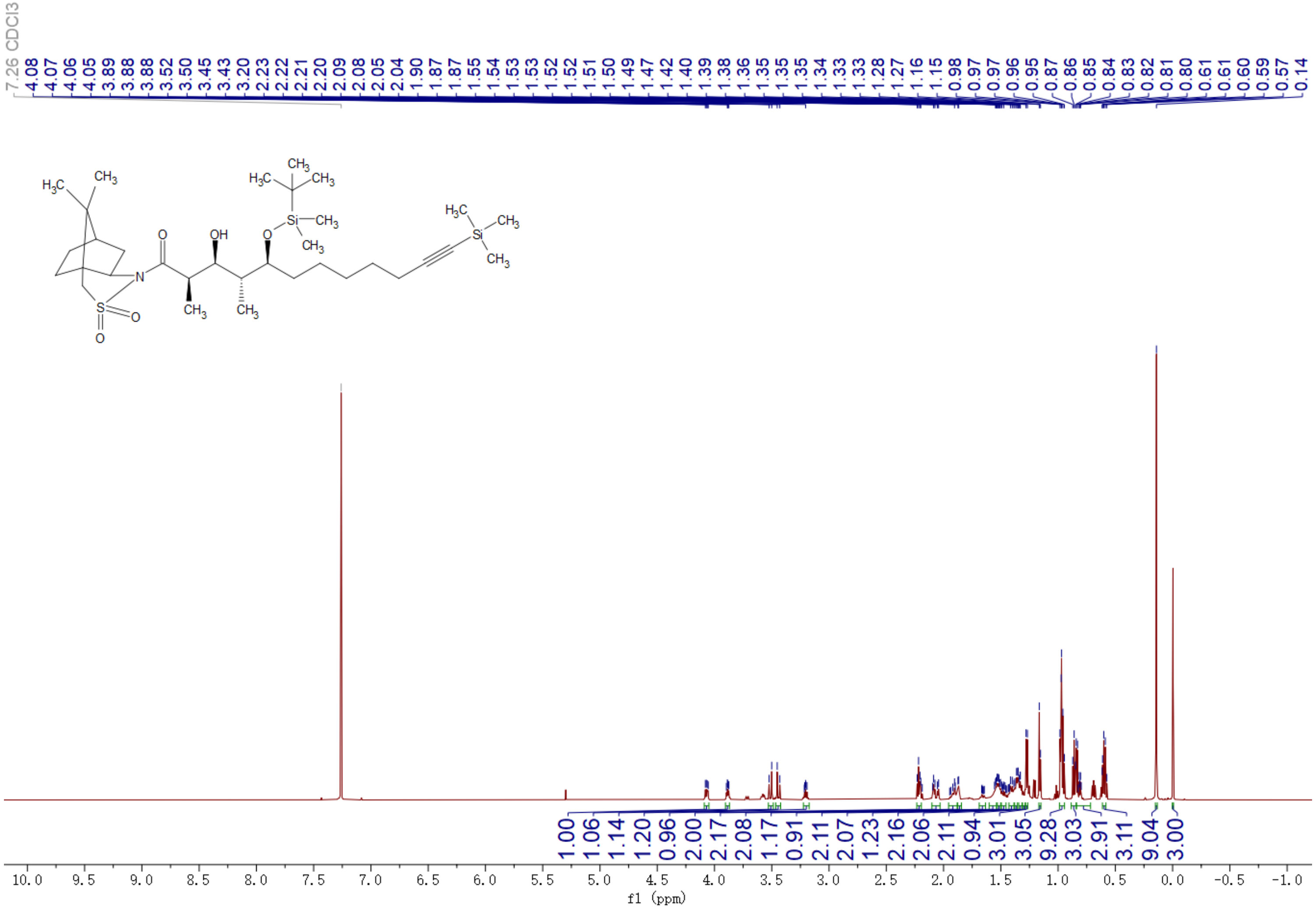

^{1}H NMR (600 MHz, $CDCl_3$) of **11**

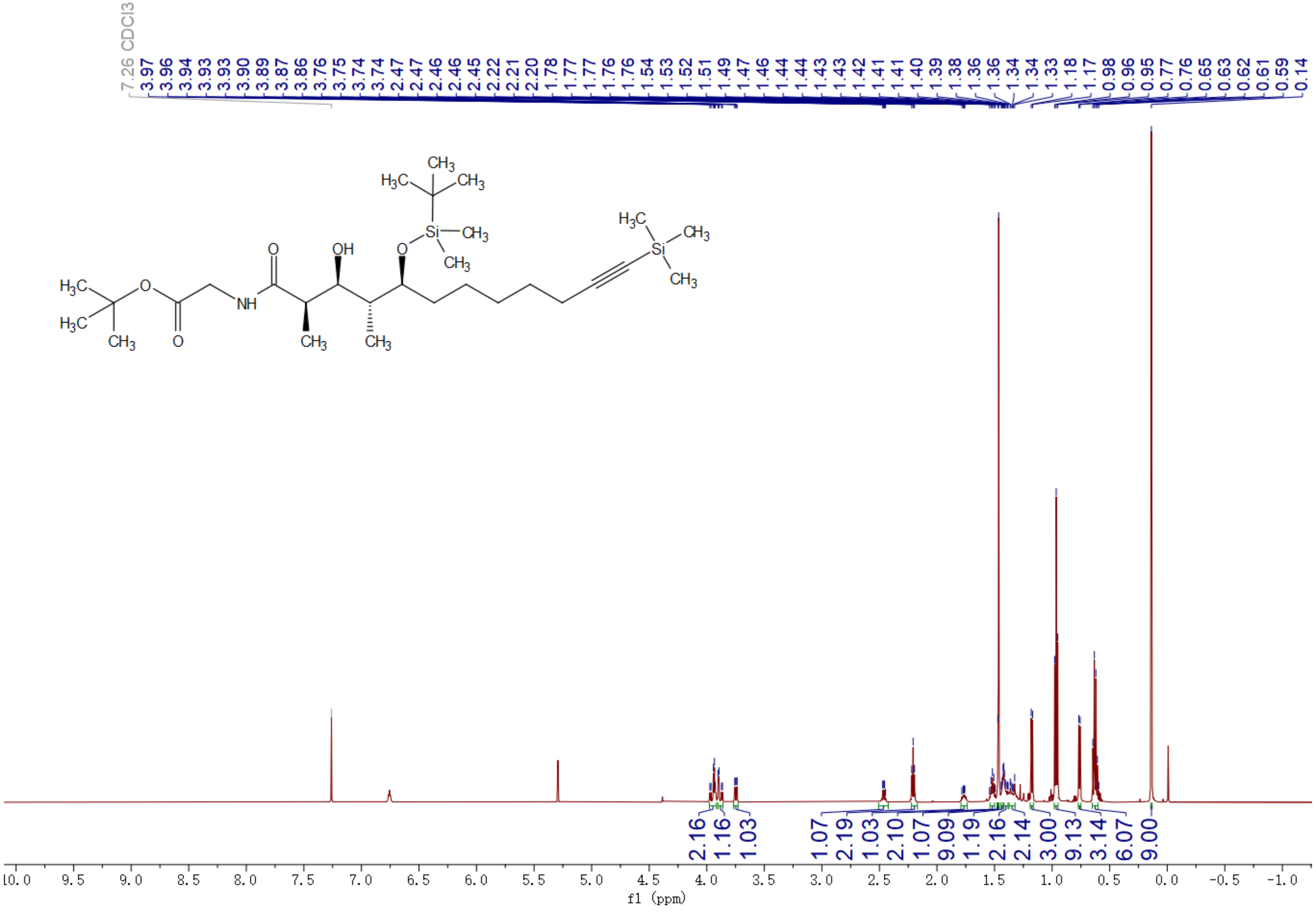

^{1}H NMR (600 MHz, $CDCl_3$) of **12**

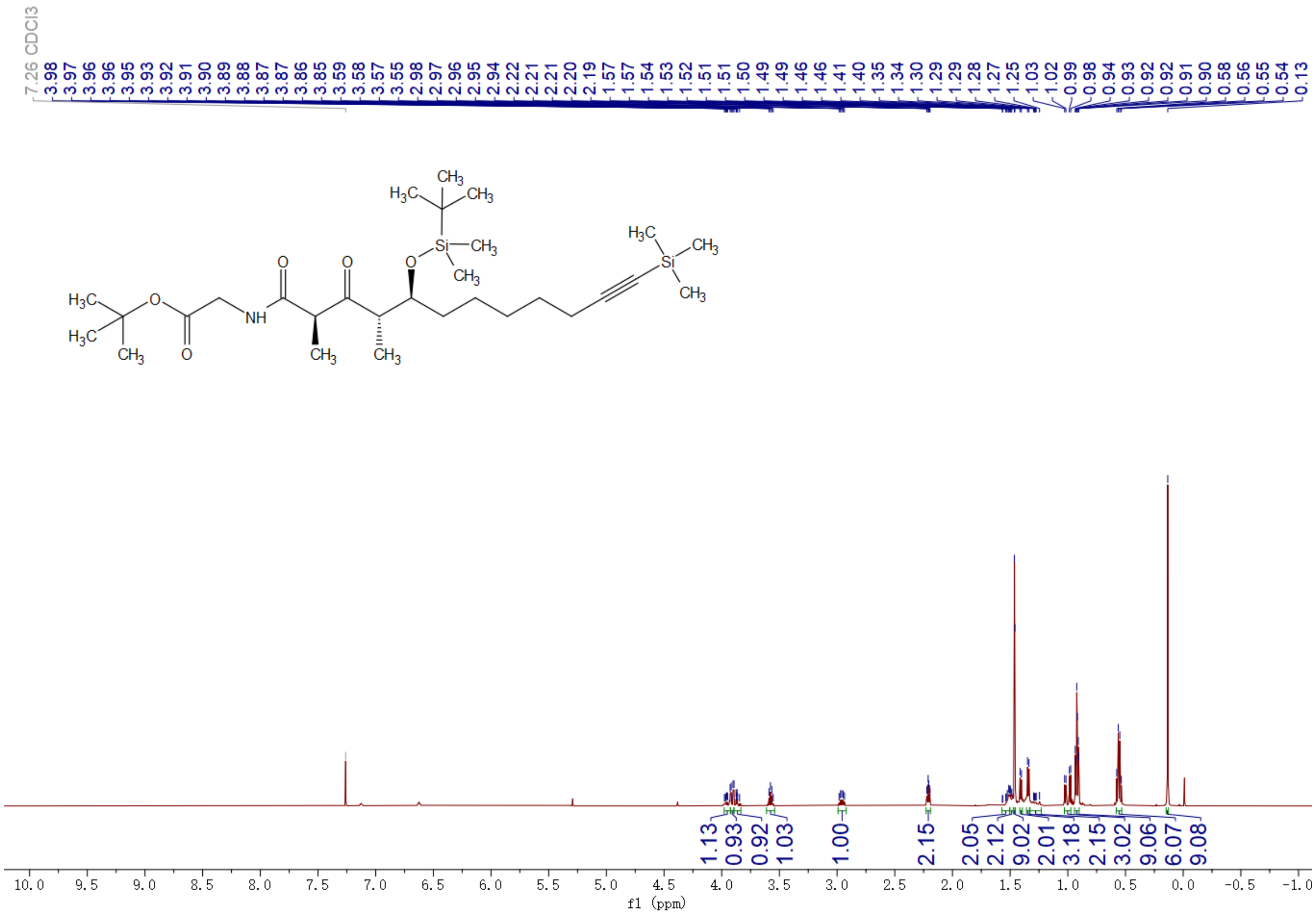

^{1}H NMR (600 MHz, $CDCl_3$) of **14**

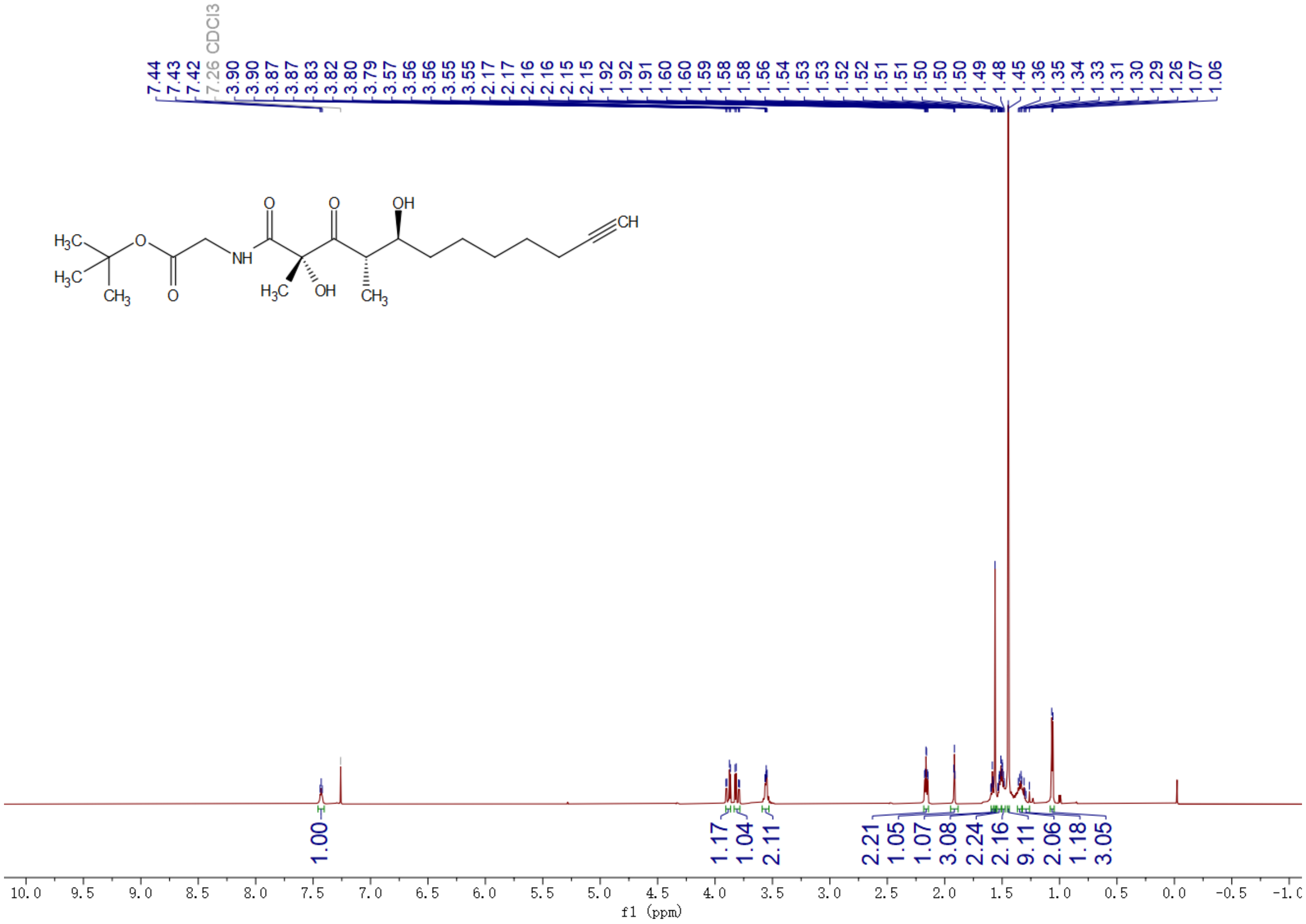

^{1}H NMR (600 MHz, DMSO-d_6) of **17**

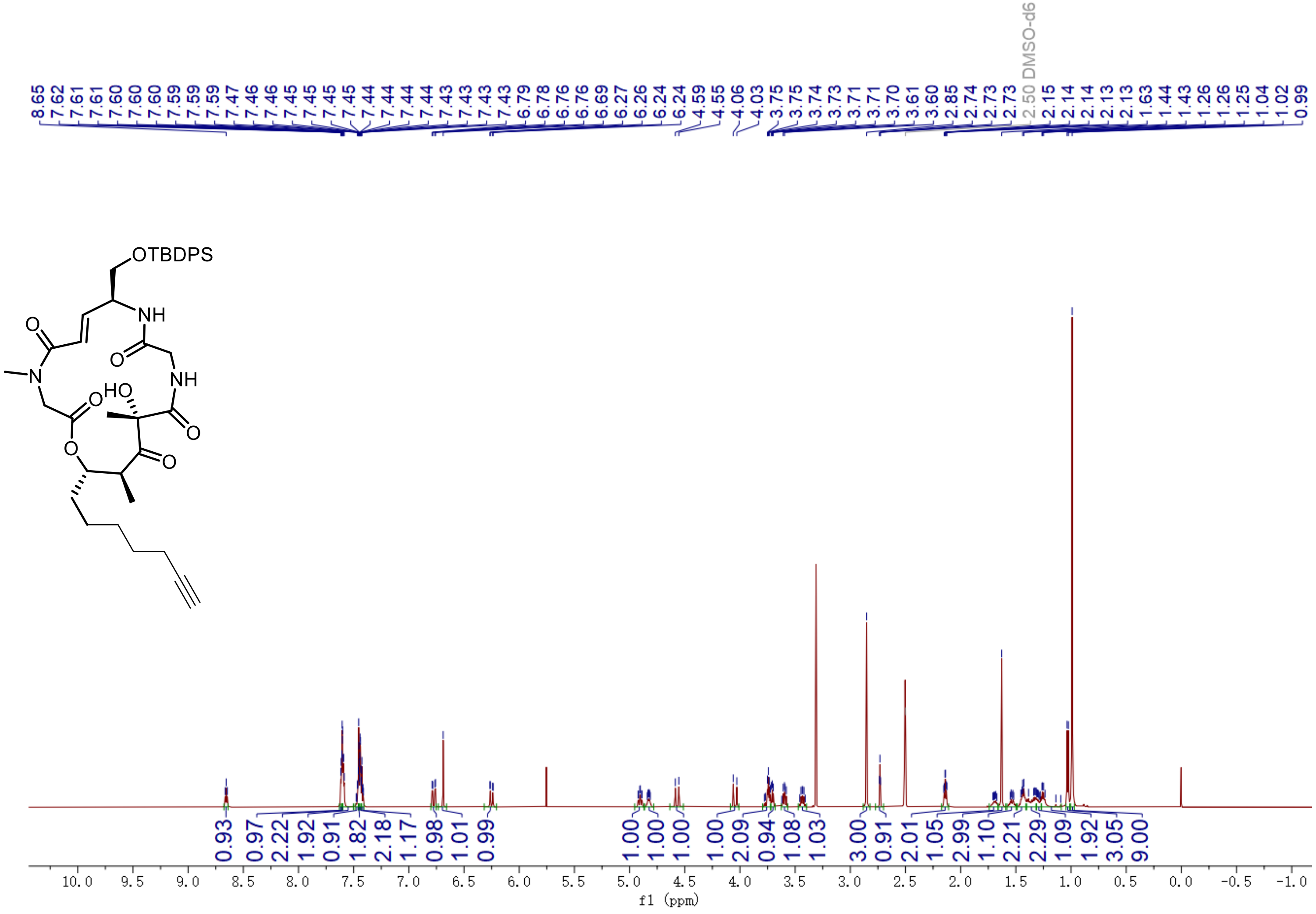

^{13}C NMR (151 MHz, DMSO-d_6) of **17**

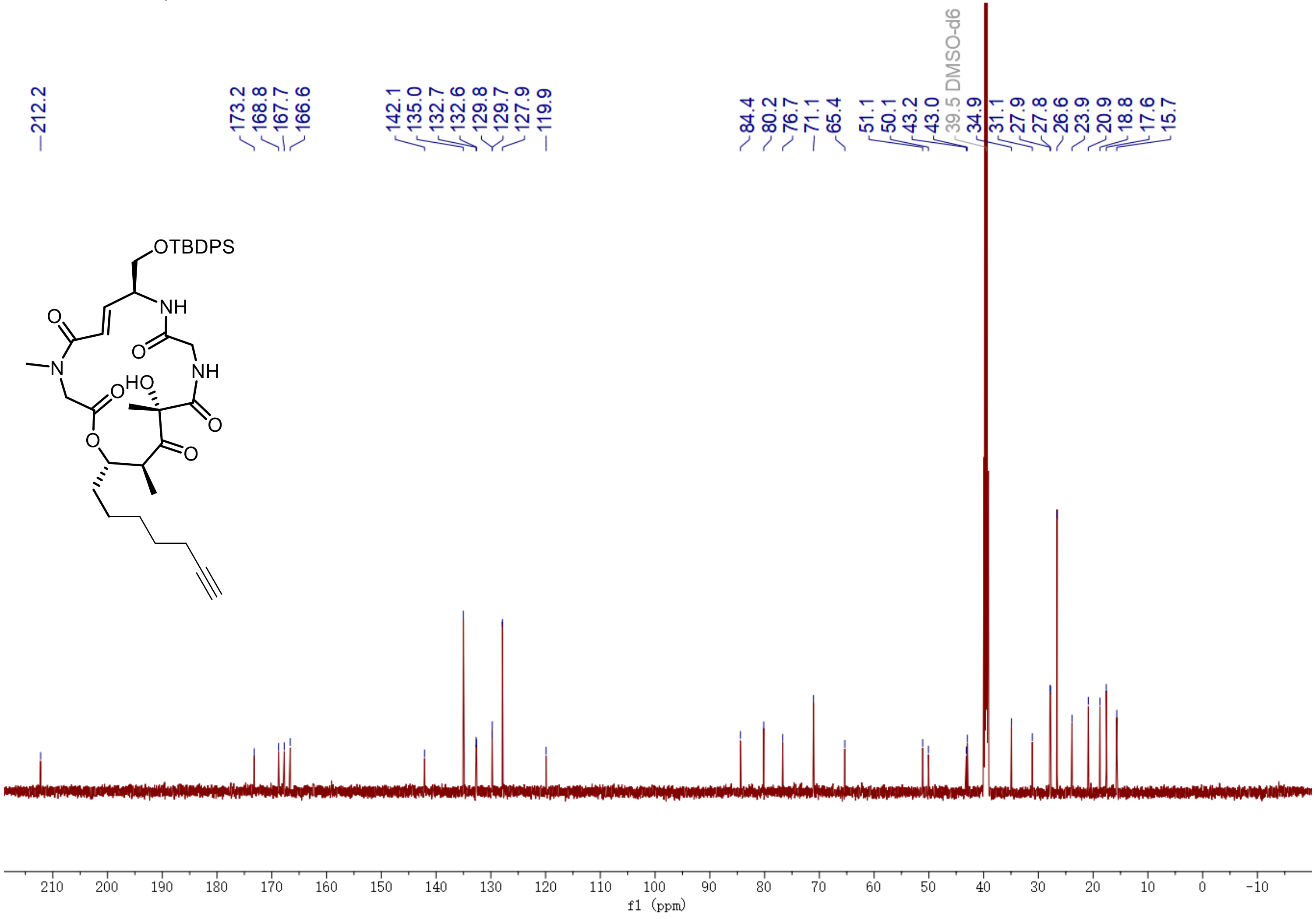

^{1}H NMR (600 MHz, DMSO-d_6) of **19**

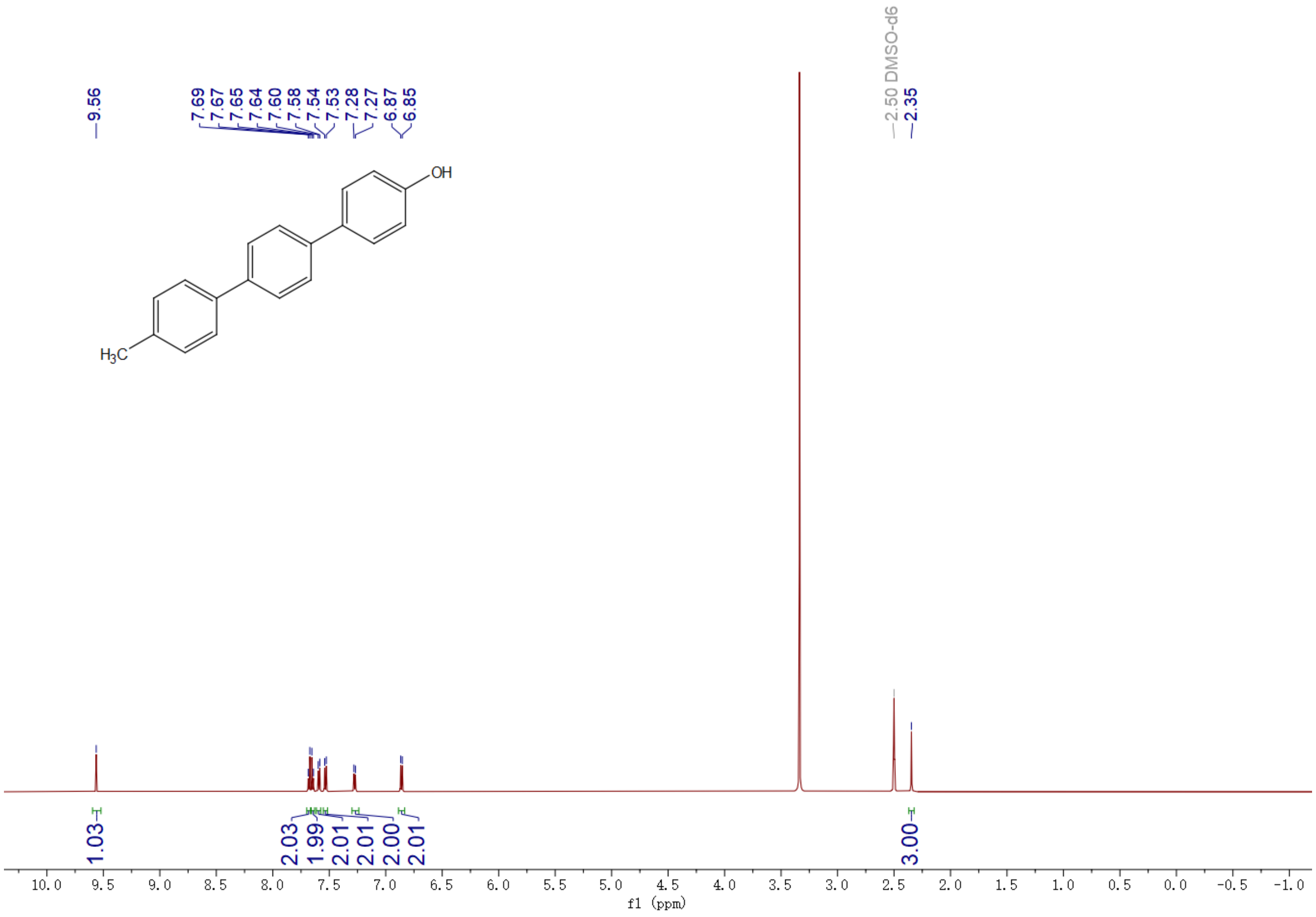

^{1}H NMR (600 MHz, DMSO-d_6) of **20**

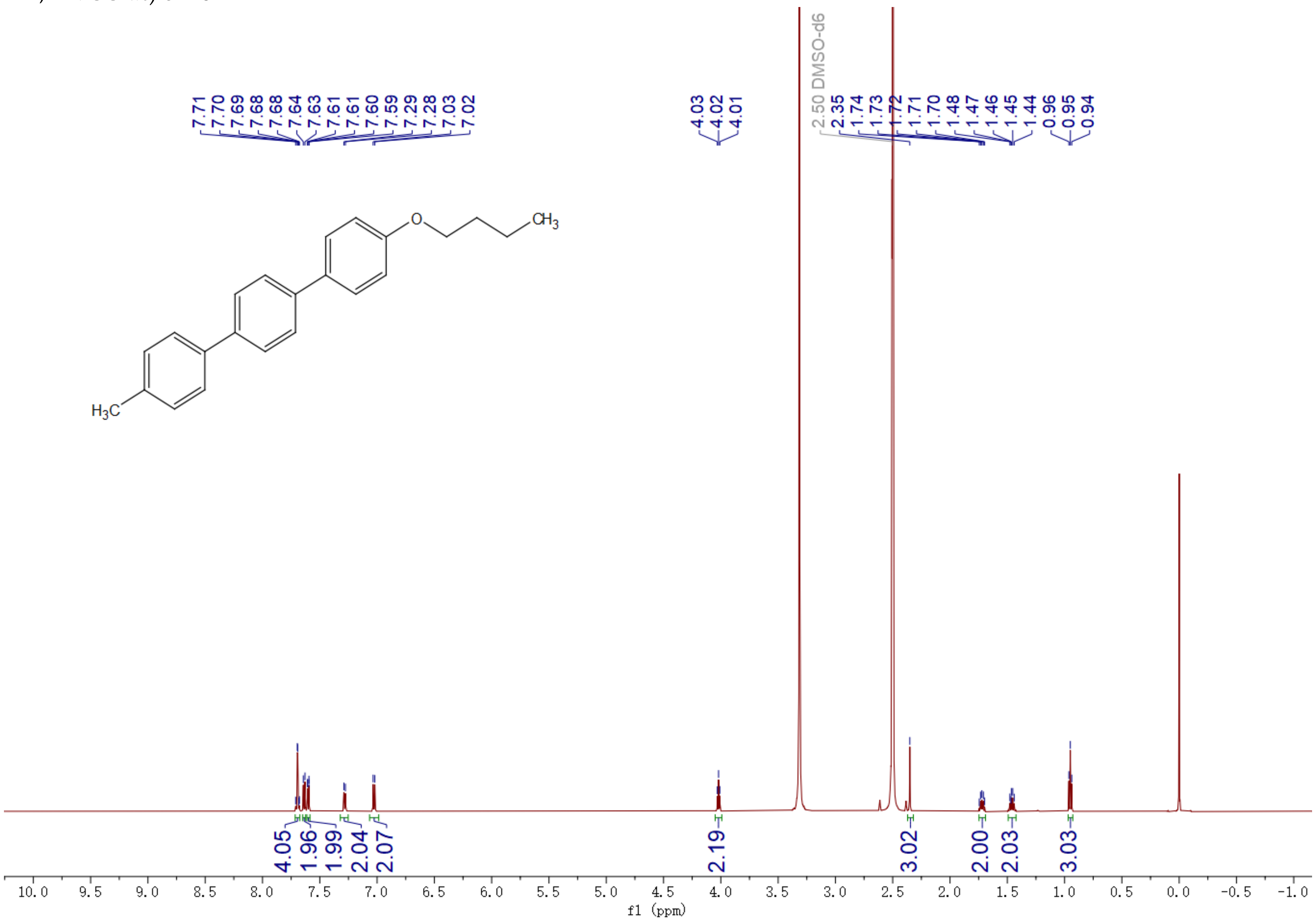

^{1}H NMR (600 MHz, DMSO-d_6) of **21**

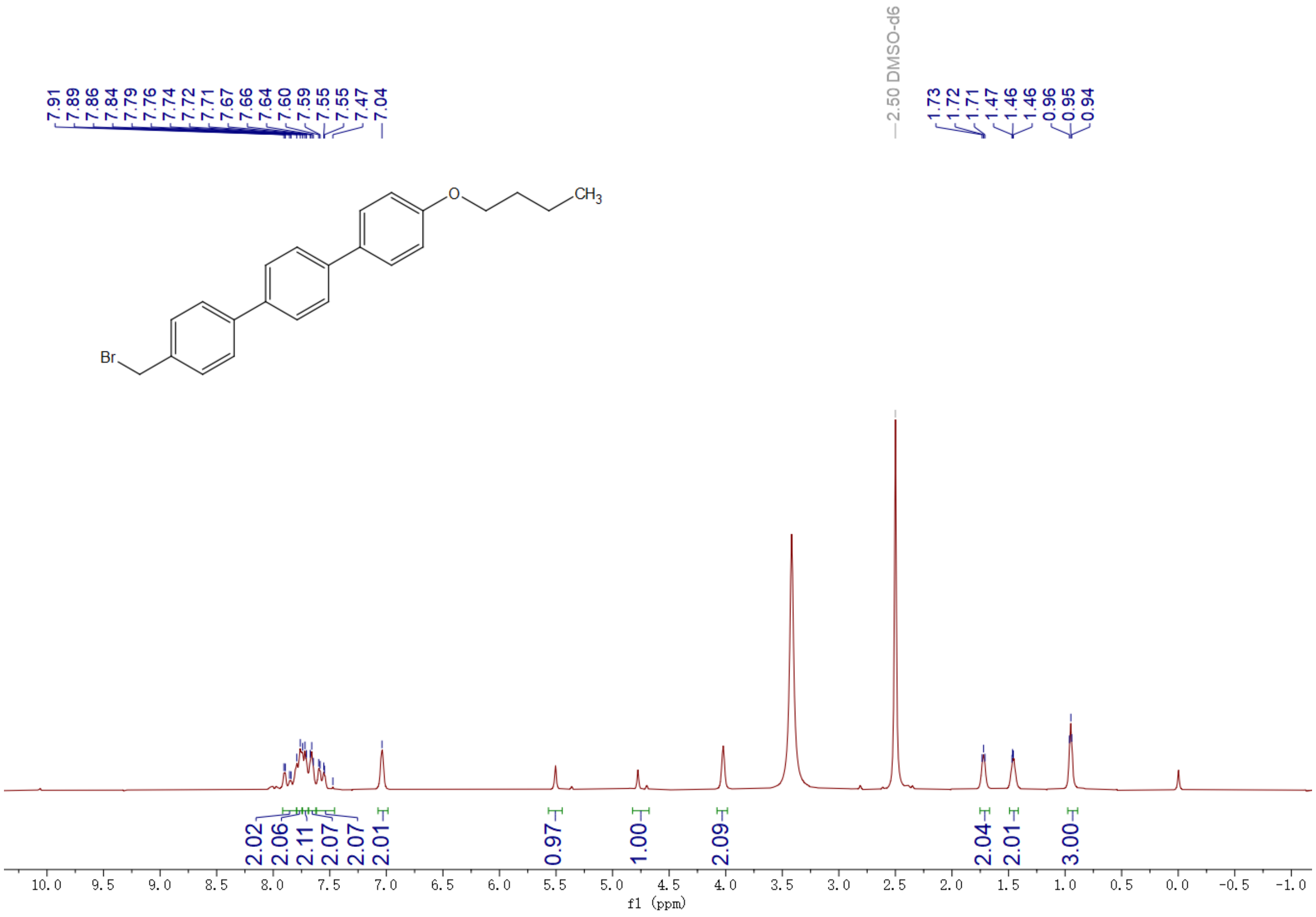

^{1}H NMR (400 MHz, $CDCl_3$) of **24**

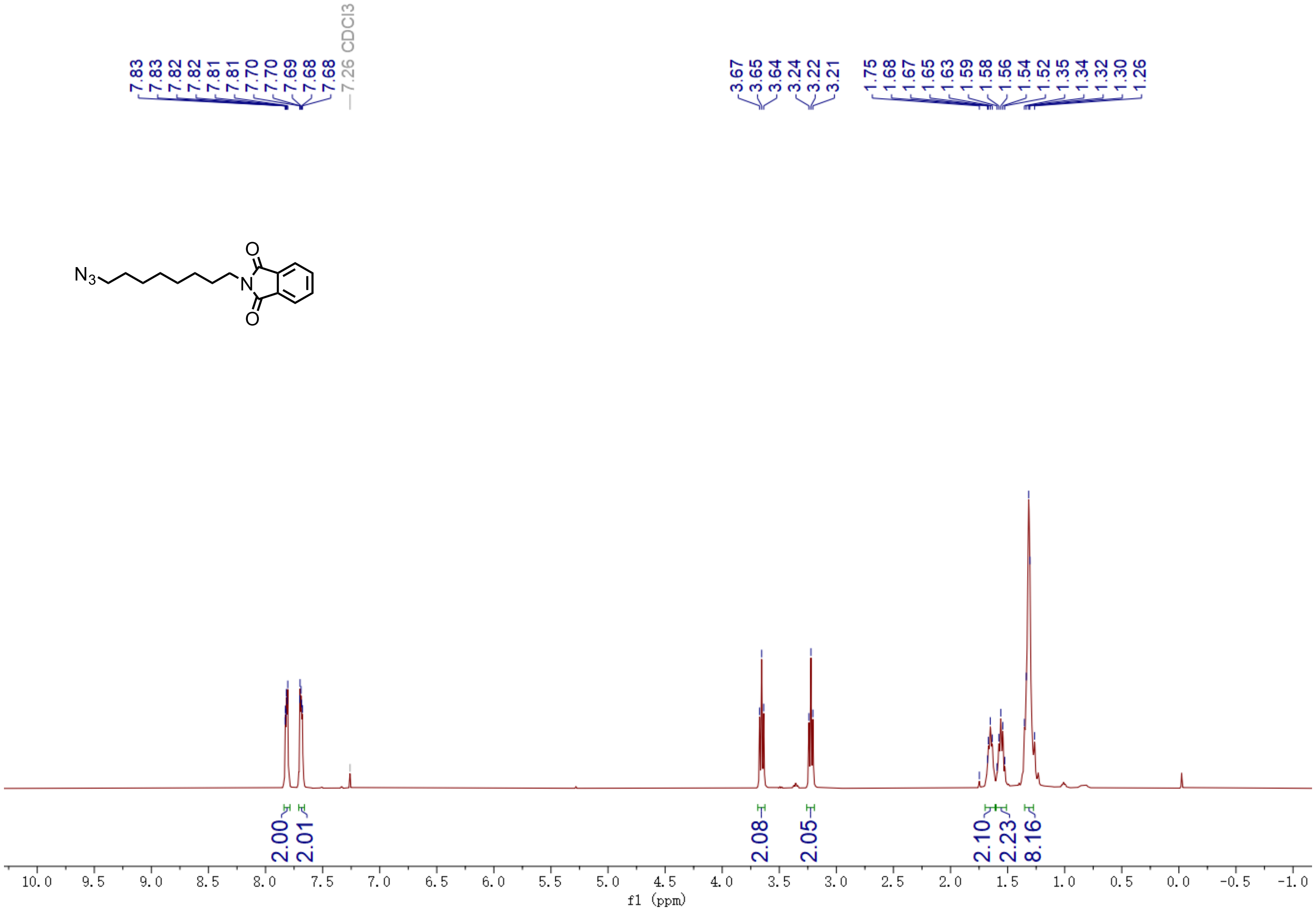

^{1}H NMR (600 MHz, $CDCl_3$) of **25a**

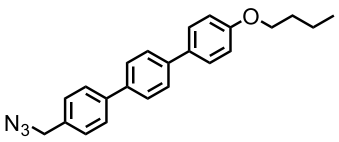

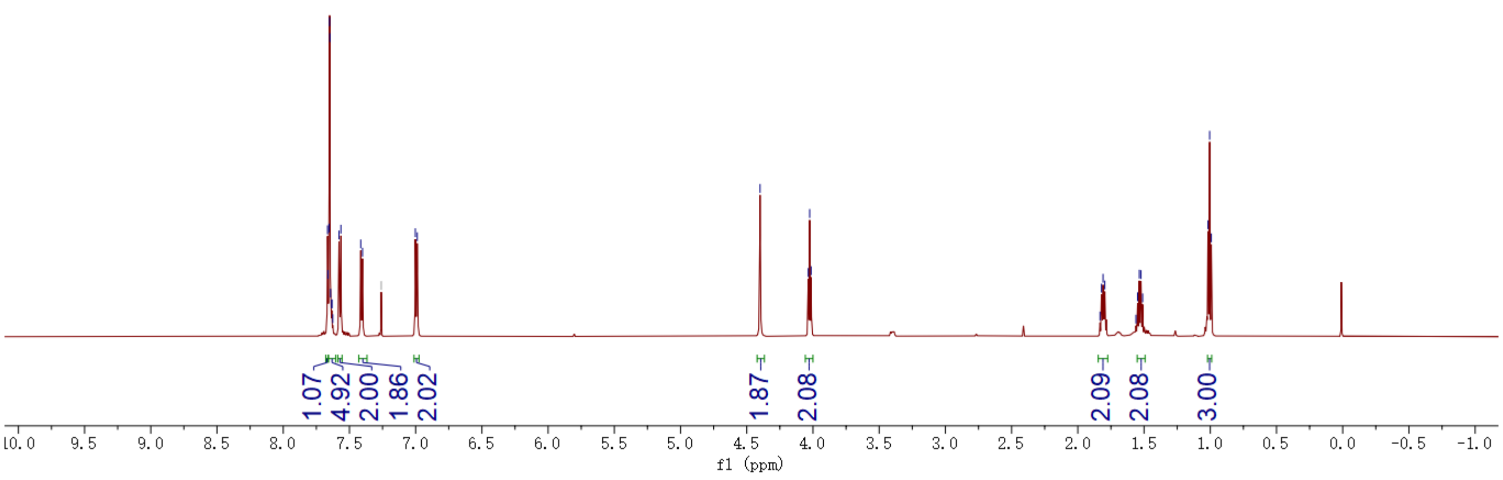

^{1}H NMR (400 MHz, $CDCl_3$) of **25b**

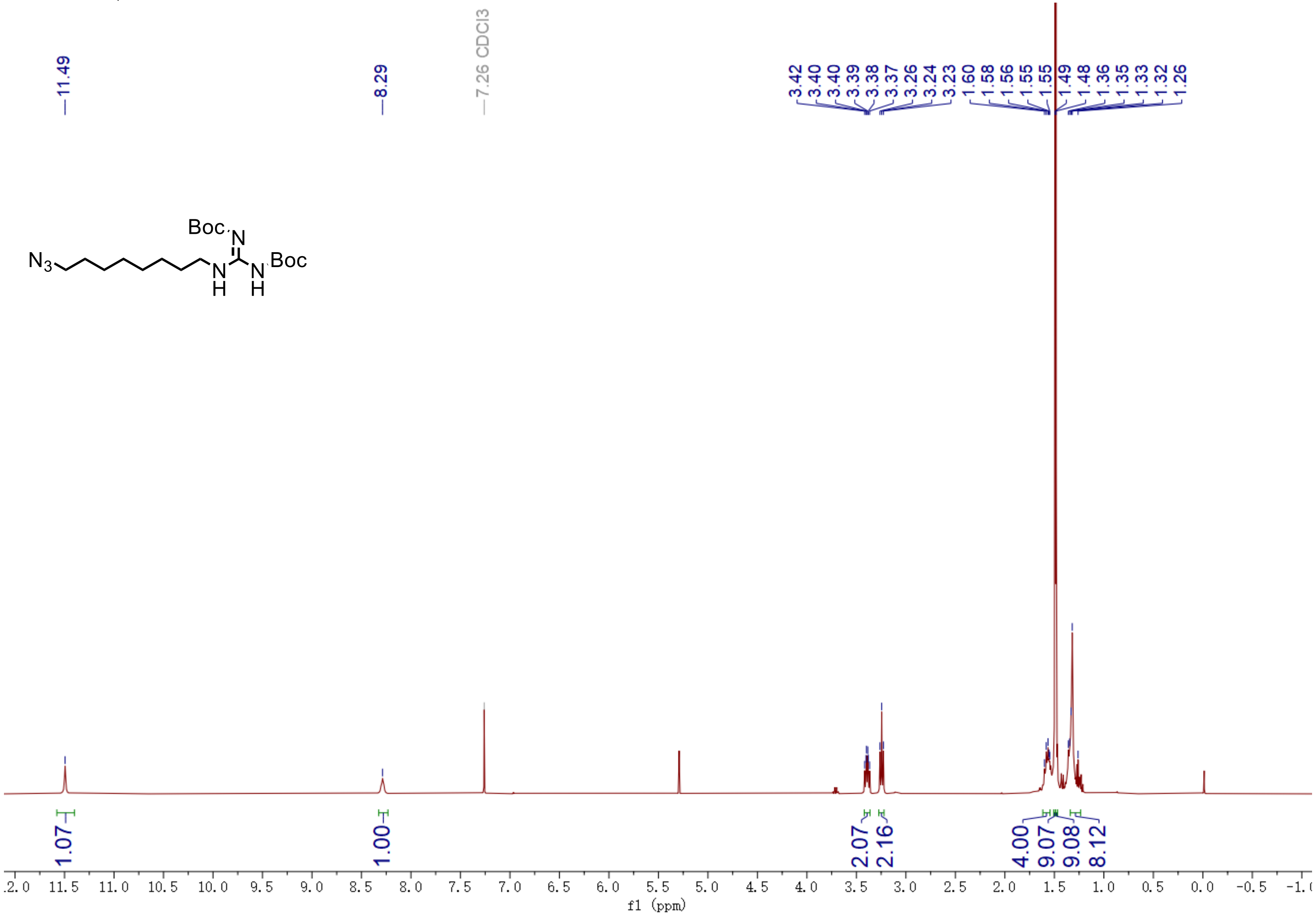

^{1}H NMR (600 MHz, $CDCl_3$) of **27a**

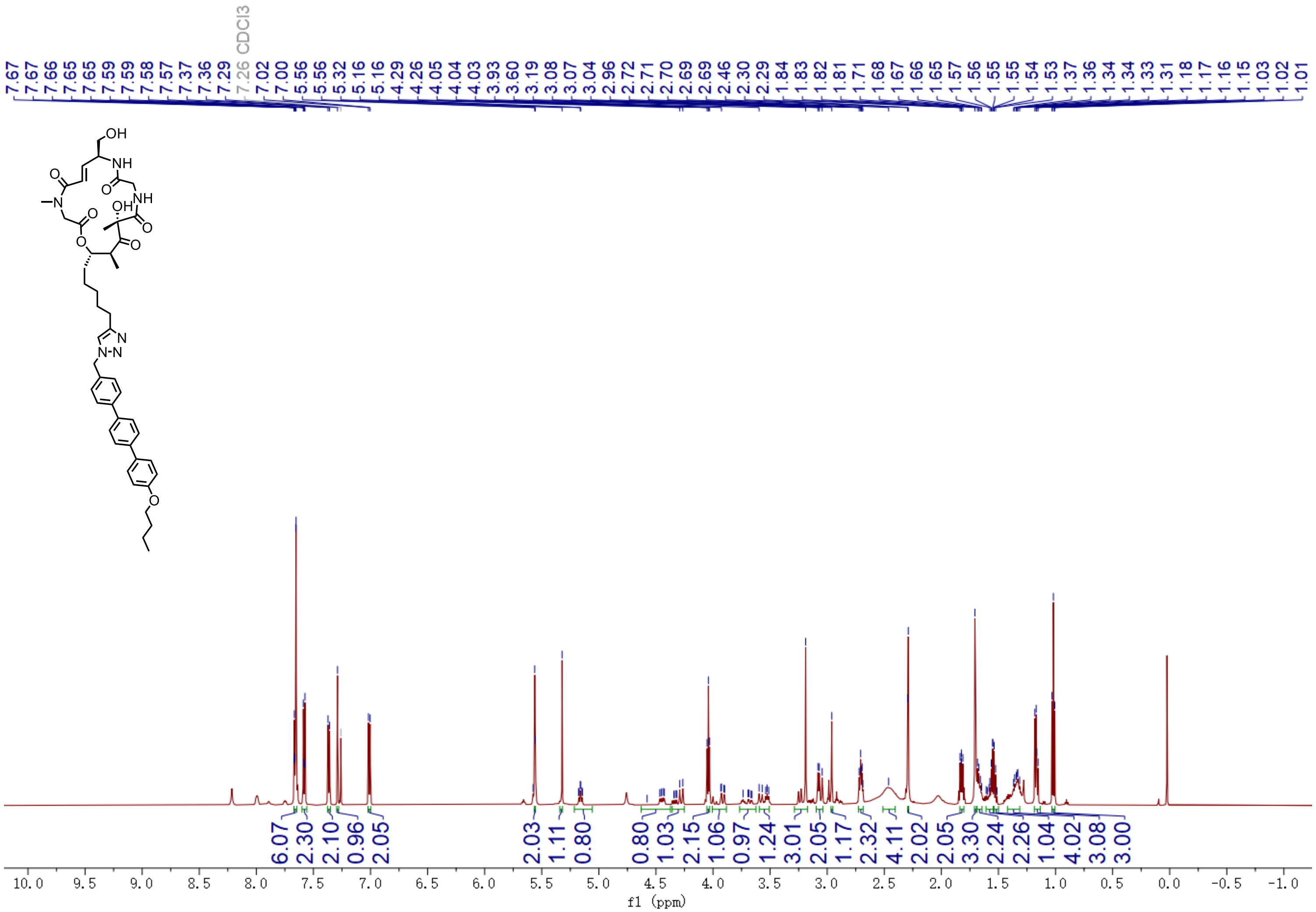

^{1}H NMR (600 MHz, DMSO-d_6) of **29**

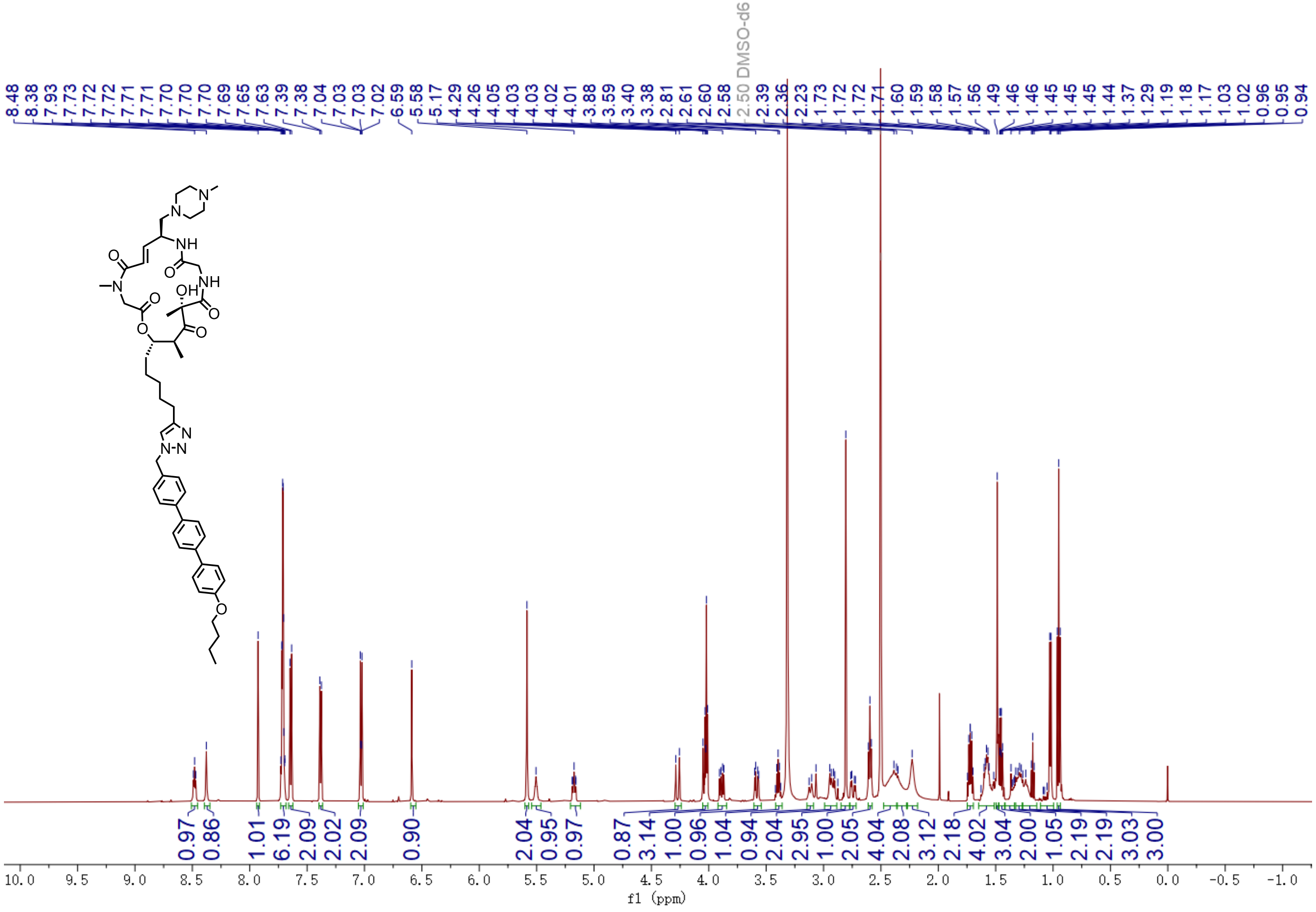

^{13}C NMR (101 MHz, DMSO-d_6) of **29**

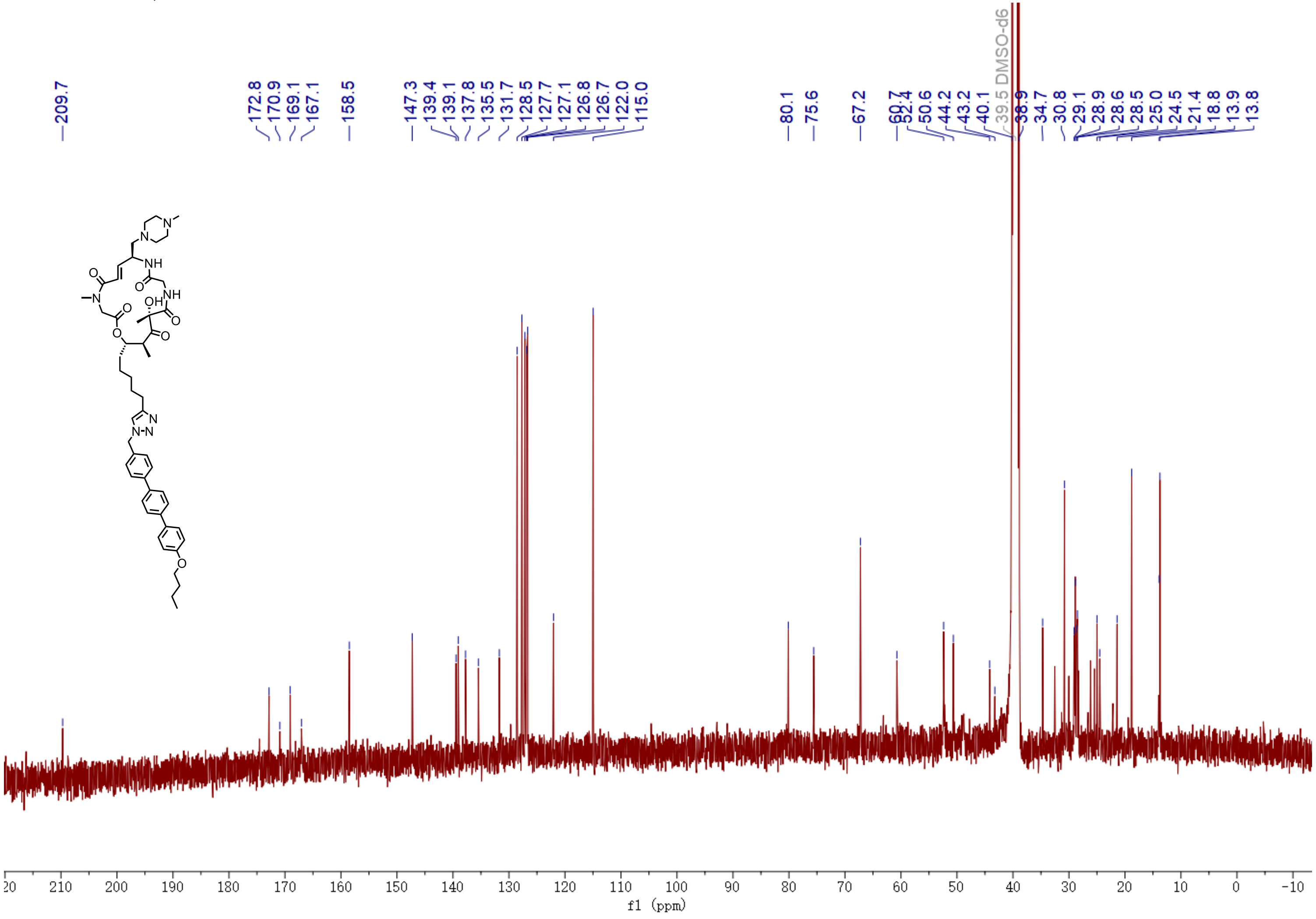

^{1}H NMR (600 MHz, DMSO-d_6) of **26b**

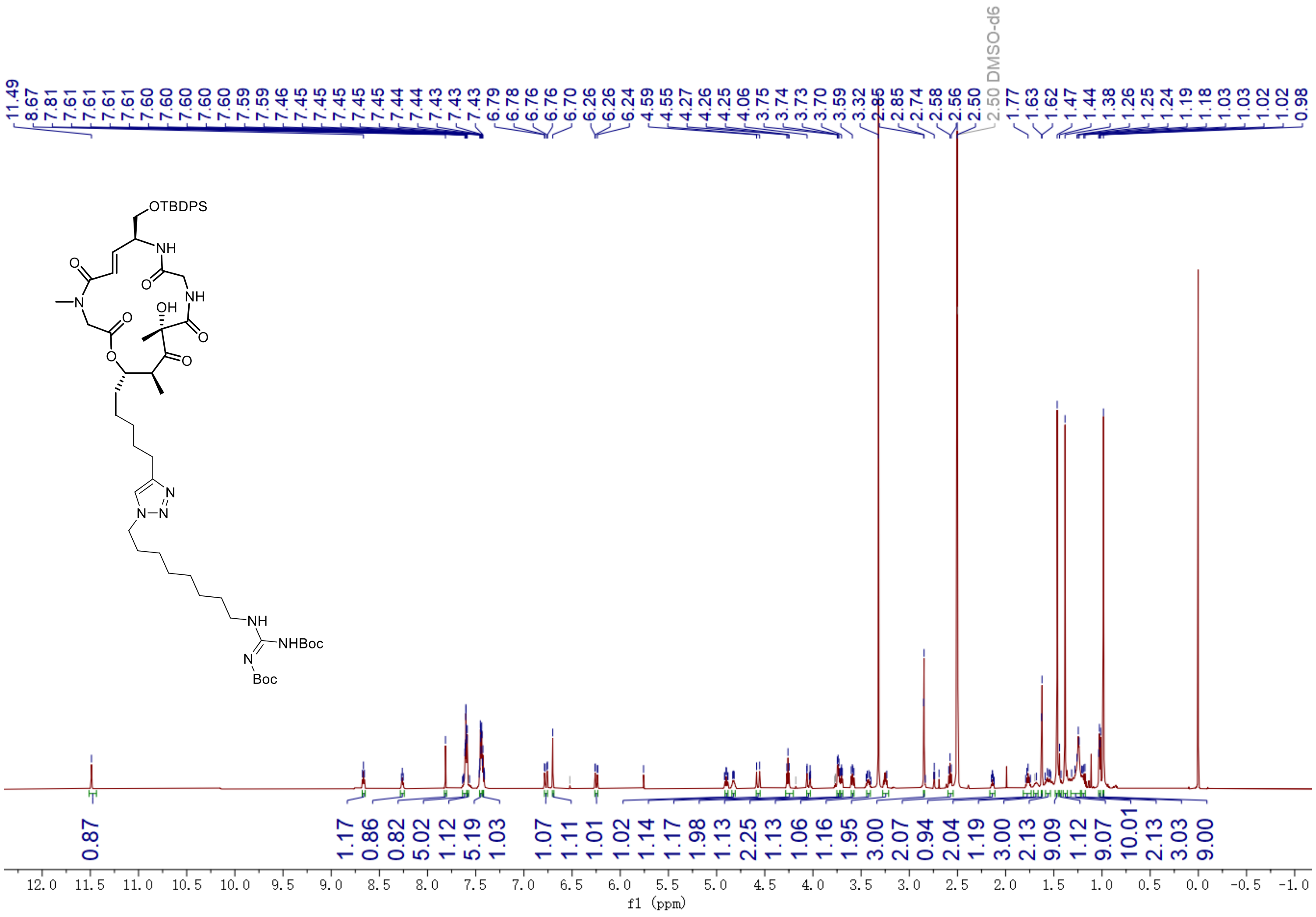

^{1}H NMR (400 MHz, DMSO-d_6) of **30**

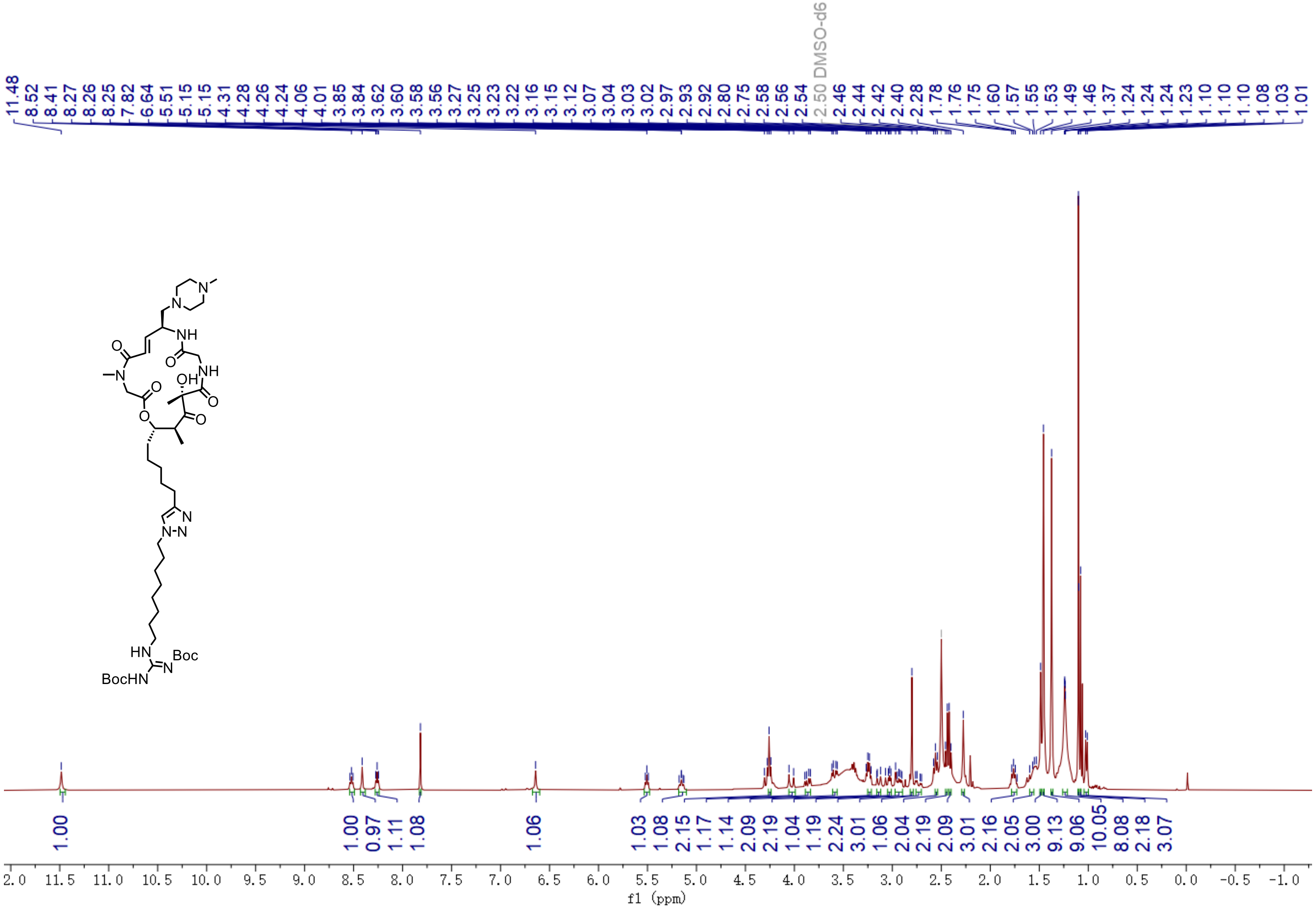

^{13}C NMR (101 MHz, DMSO-d_6) of **30**

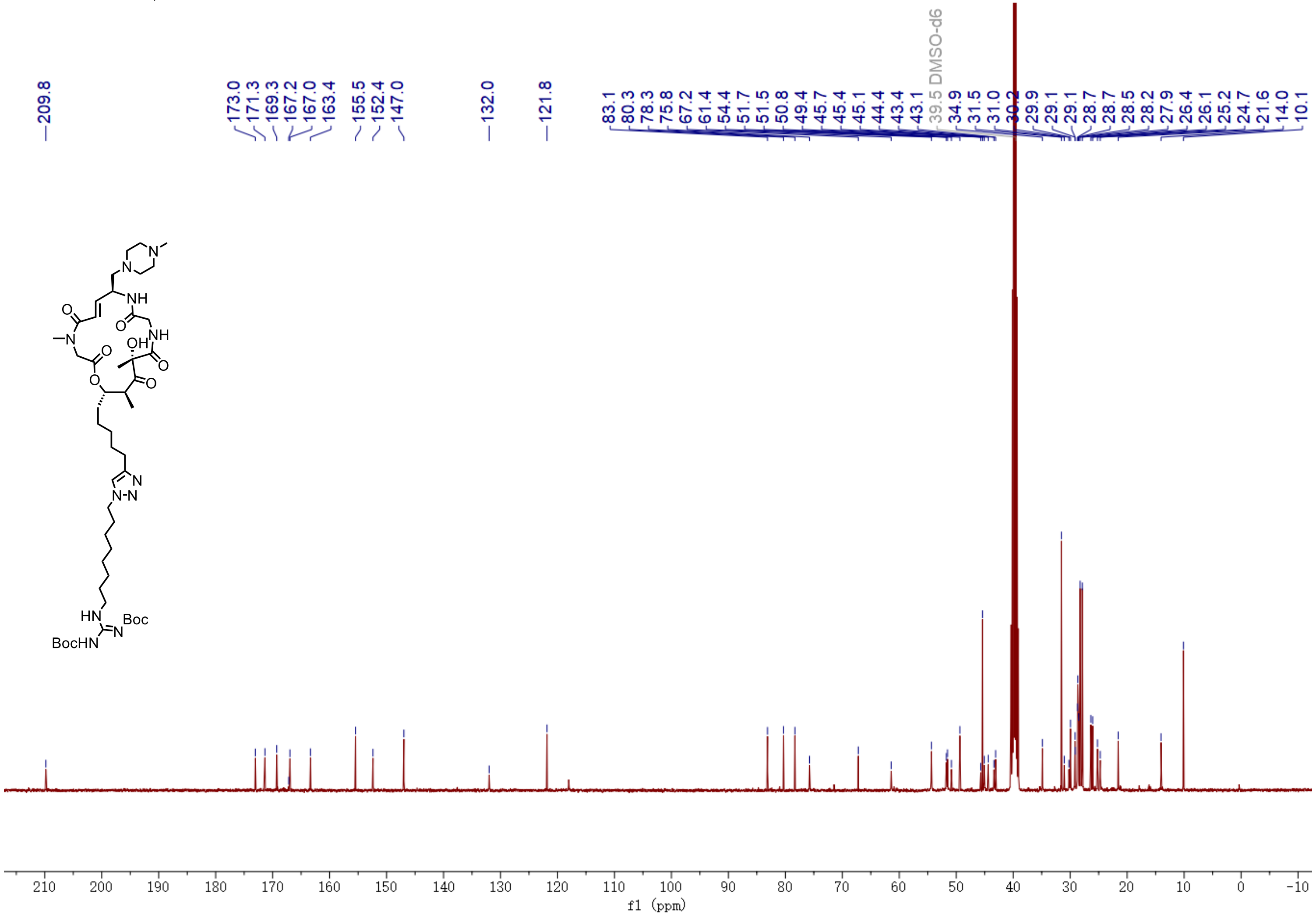

^{1}H NMR (600 MHz, DMSO-d_6) of **31**

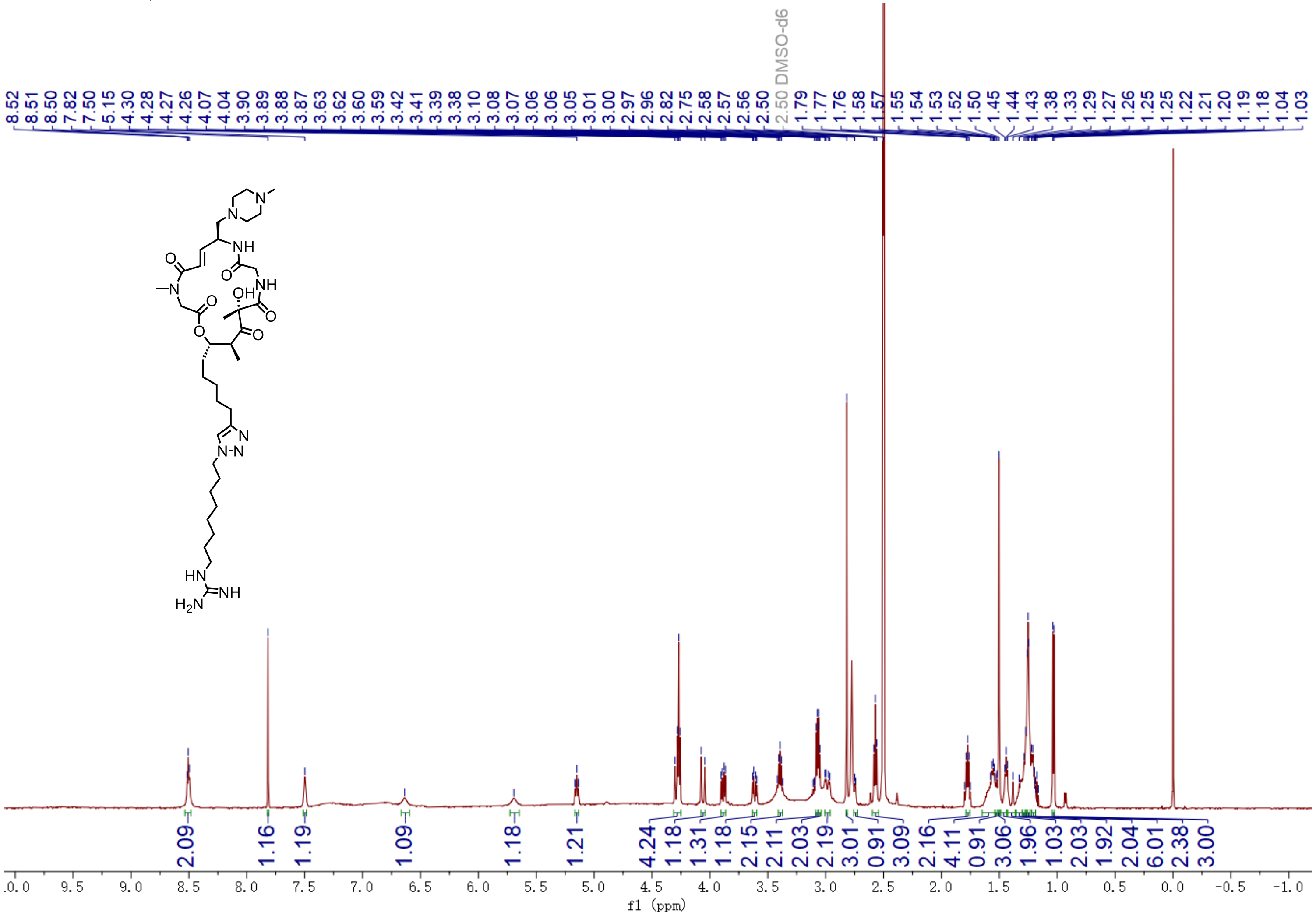

^{13}C NMR (101 MHz, DMSO-d_6) of **31**

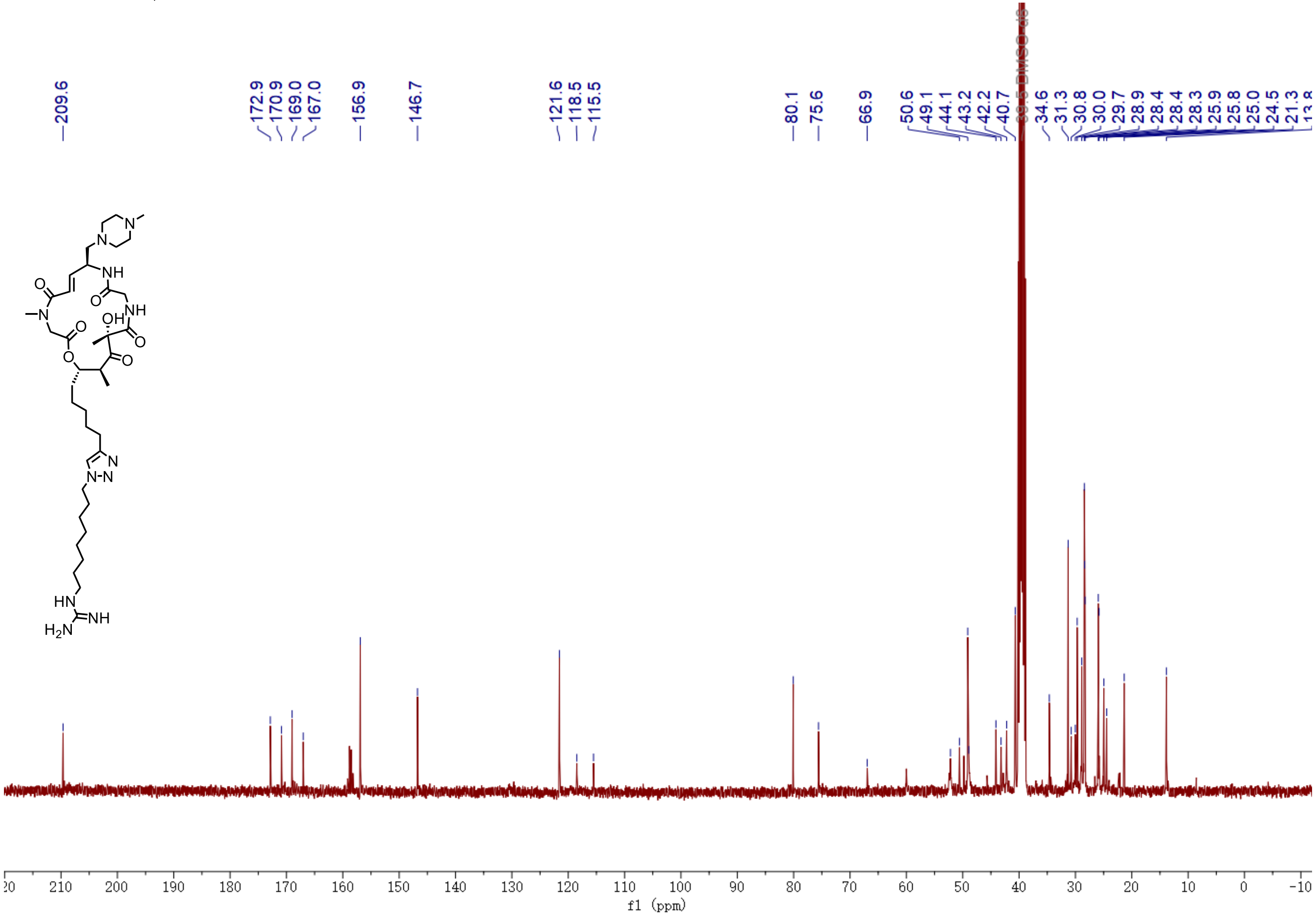

^{1}H NMR (600 MHz, DMSO-d_6) of **32**

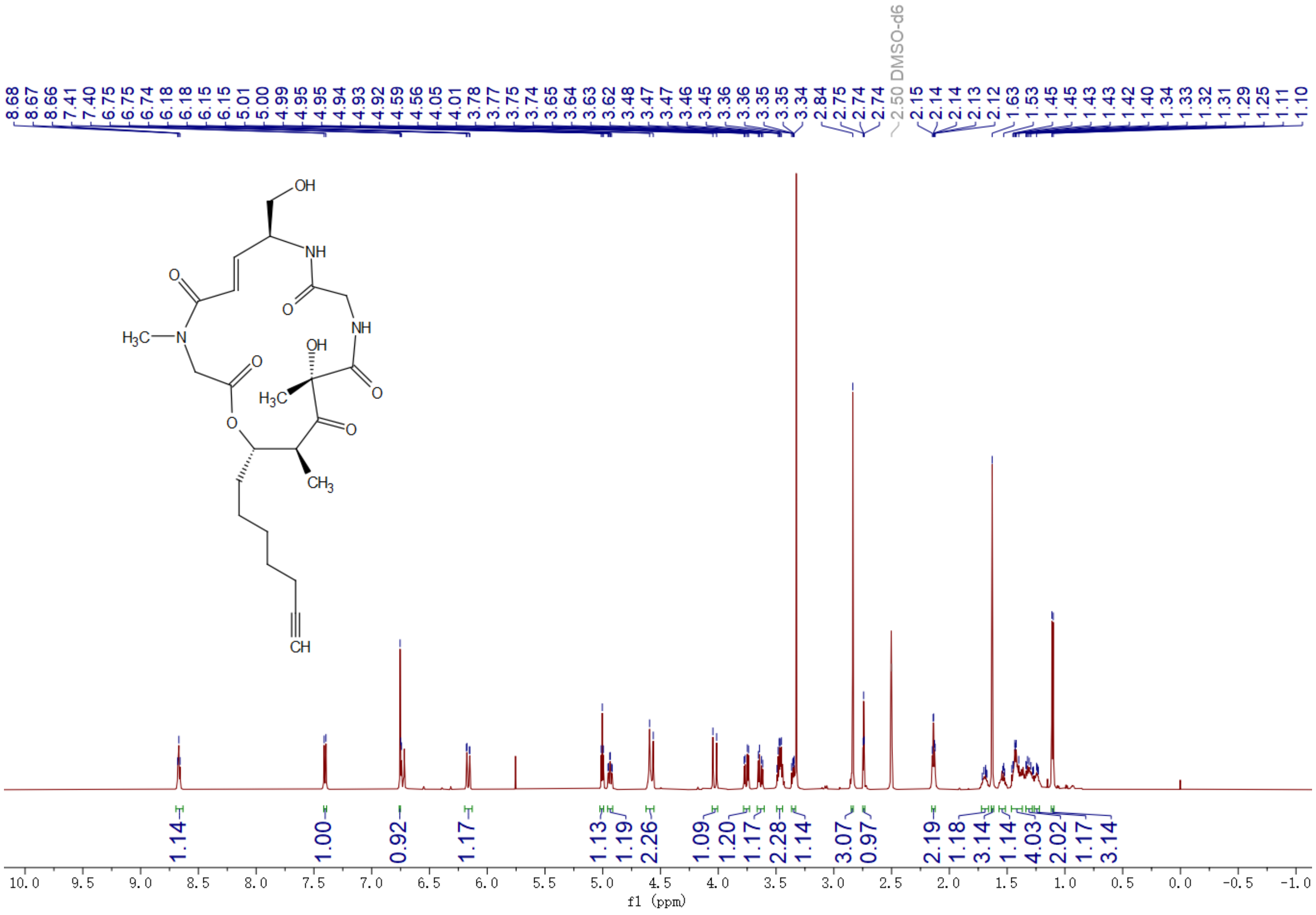

^{1}H NMR (400 MHz, DMSO-d_6) of **37**

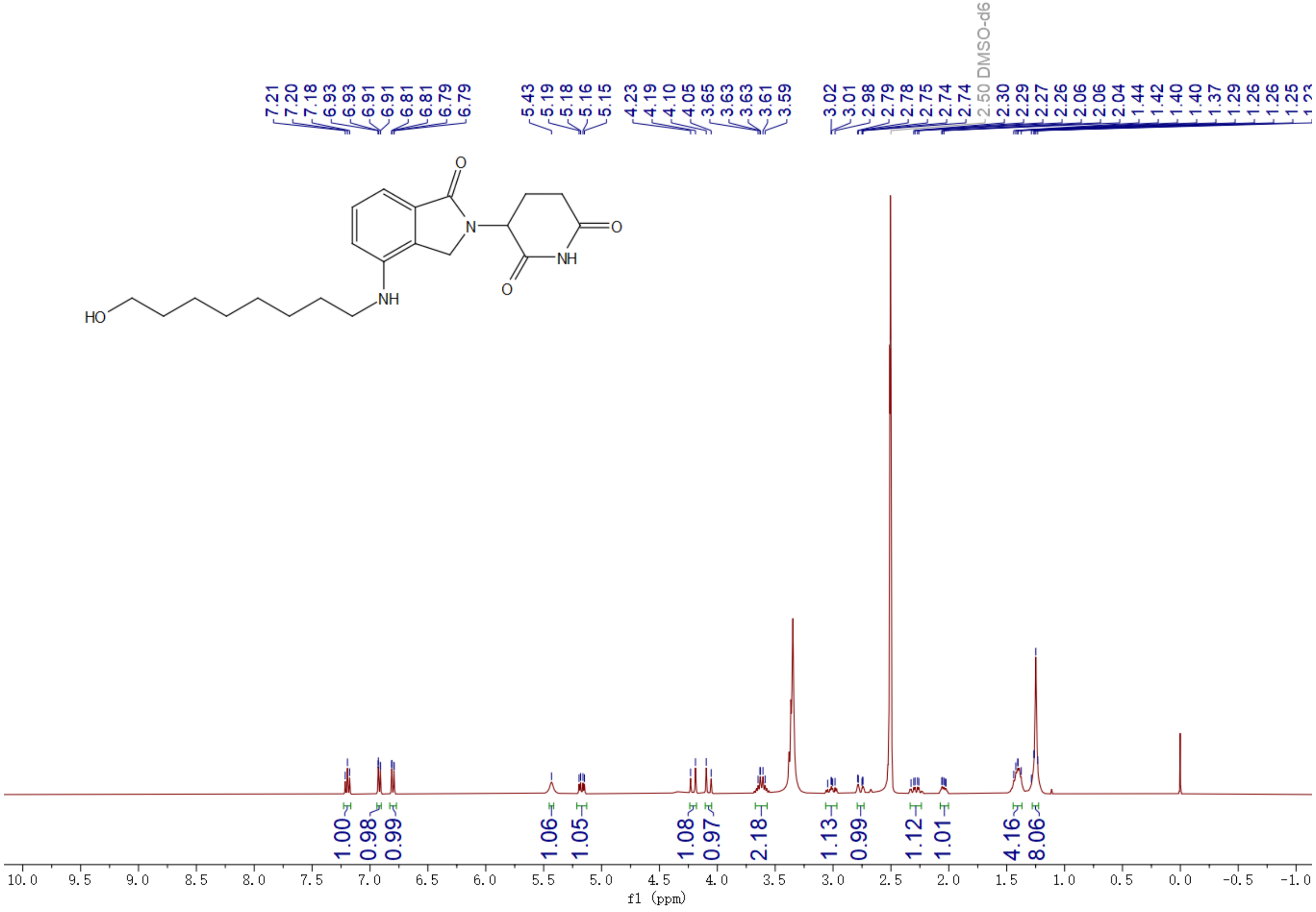

^{13}C NMR (101 MHz, DMSO-d_6) of **37**

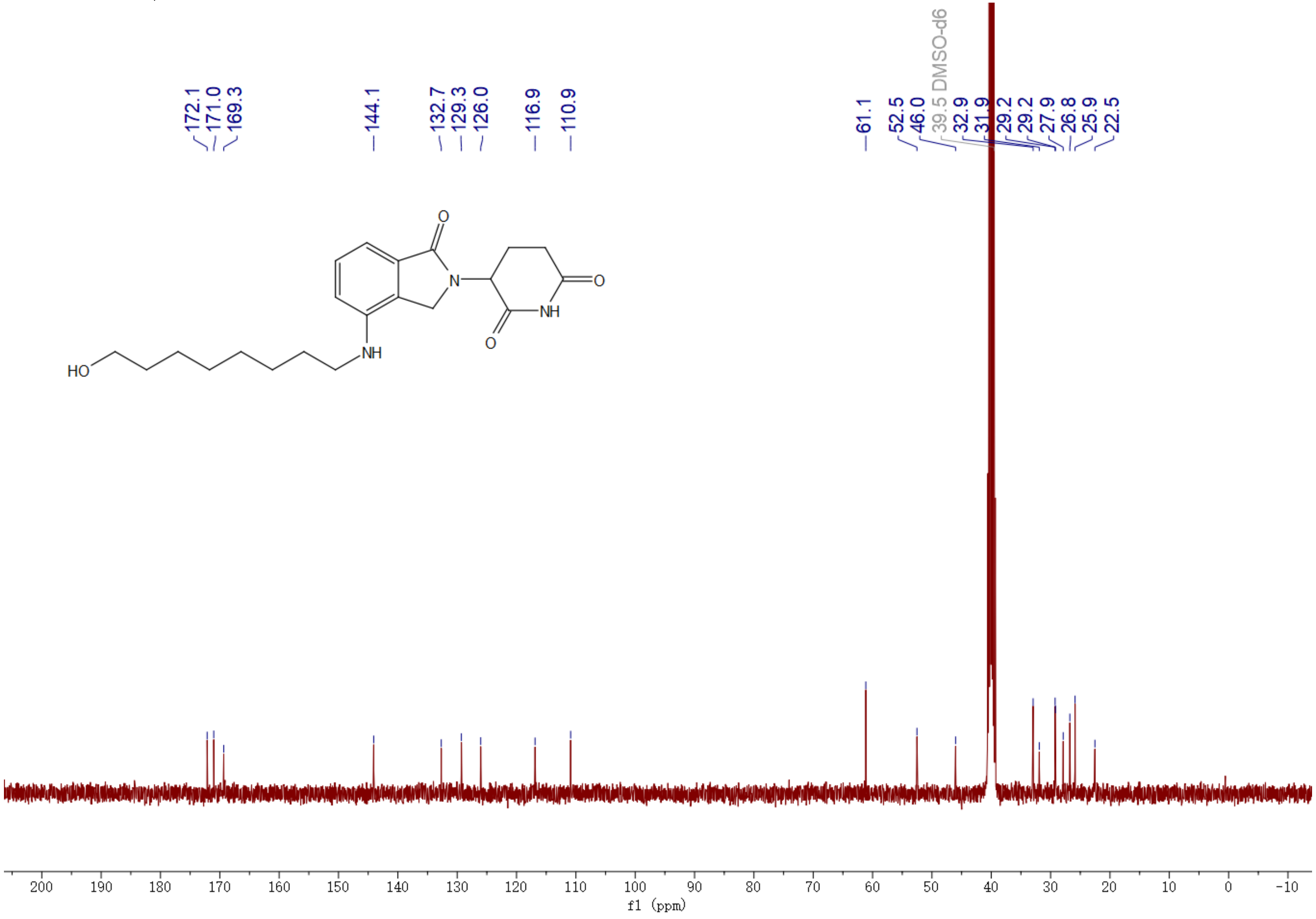

^{1}H NMR (600 MHz, $CDCl_3$) of **38a**

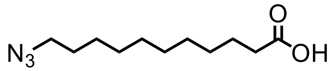

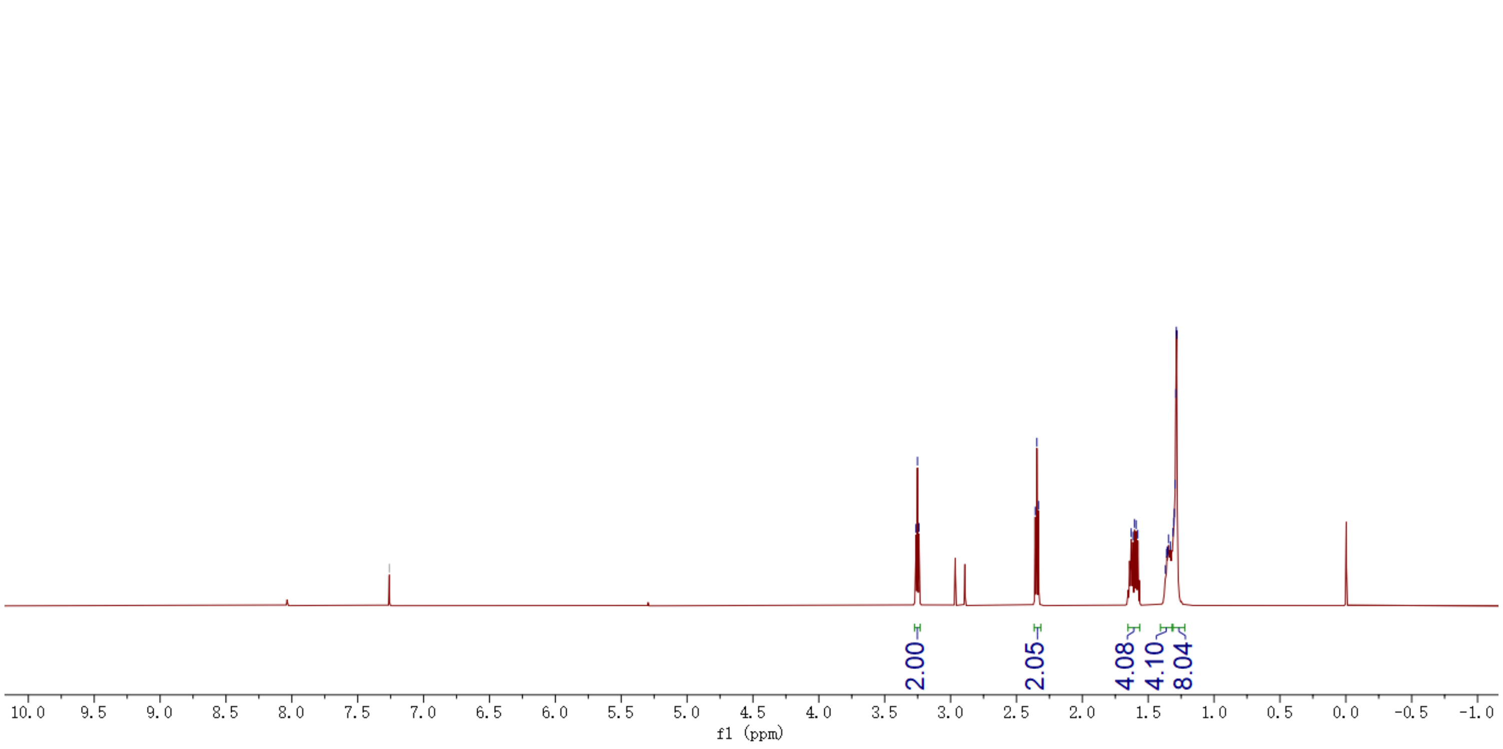

^{1}H NMR (400 MHz, DMSO-d_6) of **39**

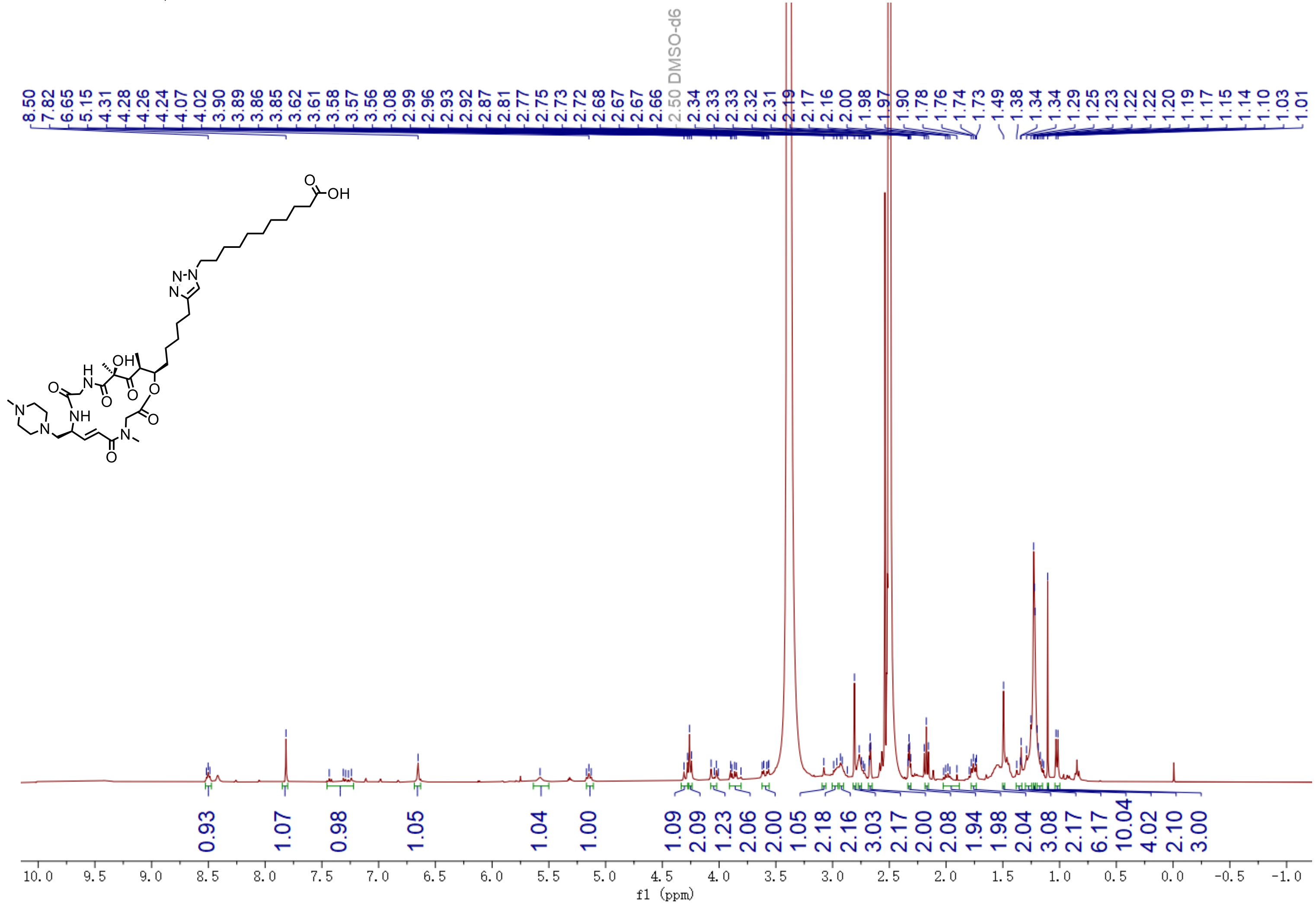

^{1}H NMR (600 MHz, DMSO-d_6) of **40**

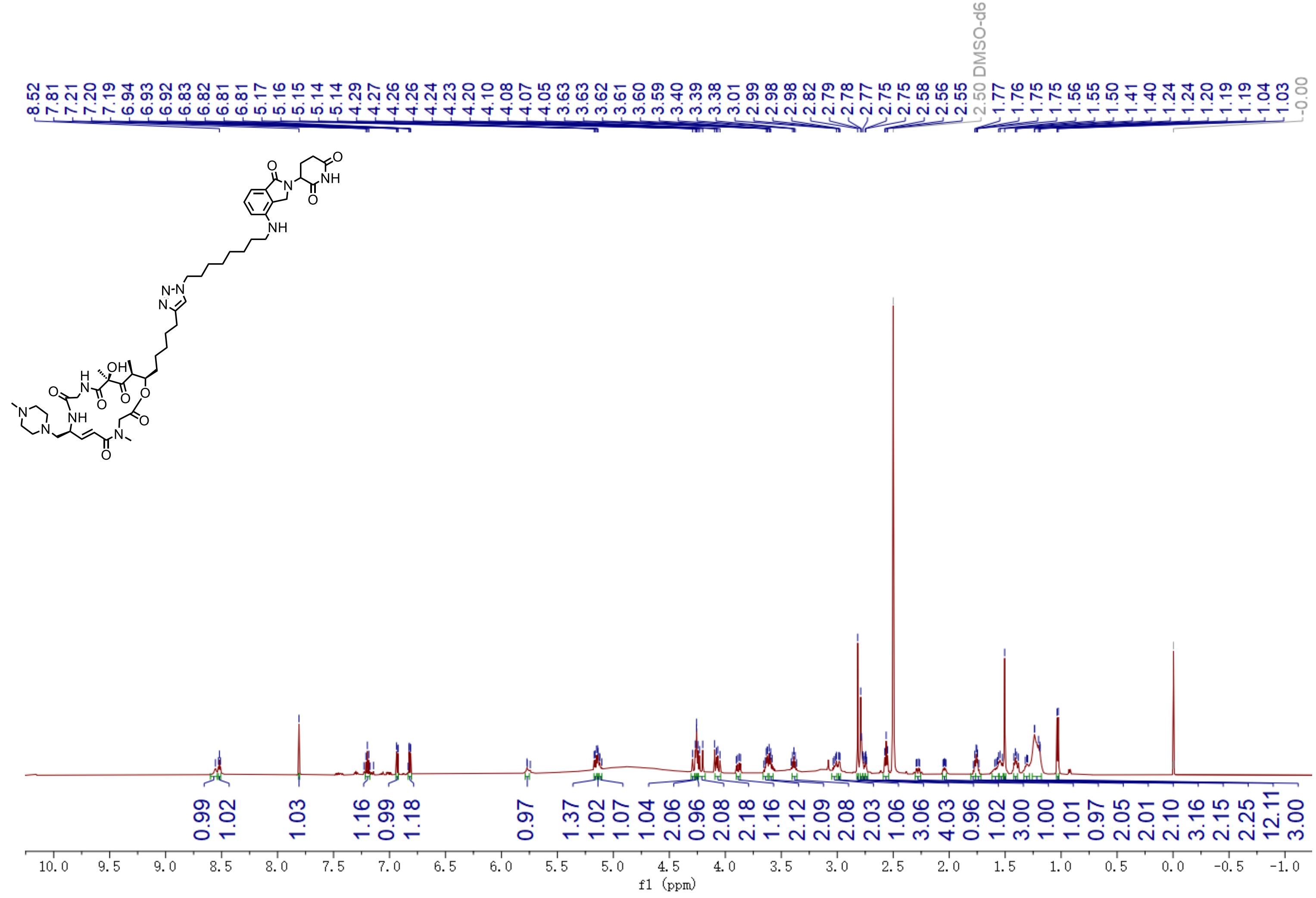

^{13}C NMR (101 MHz, DMSO-d_6) of **40**

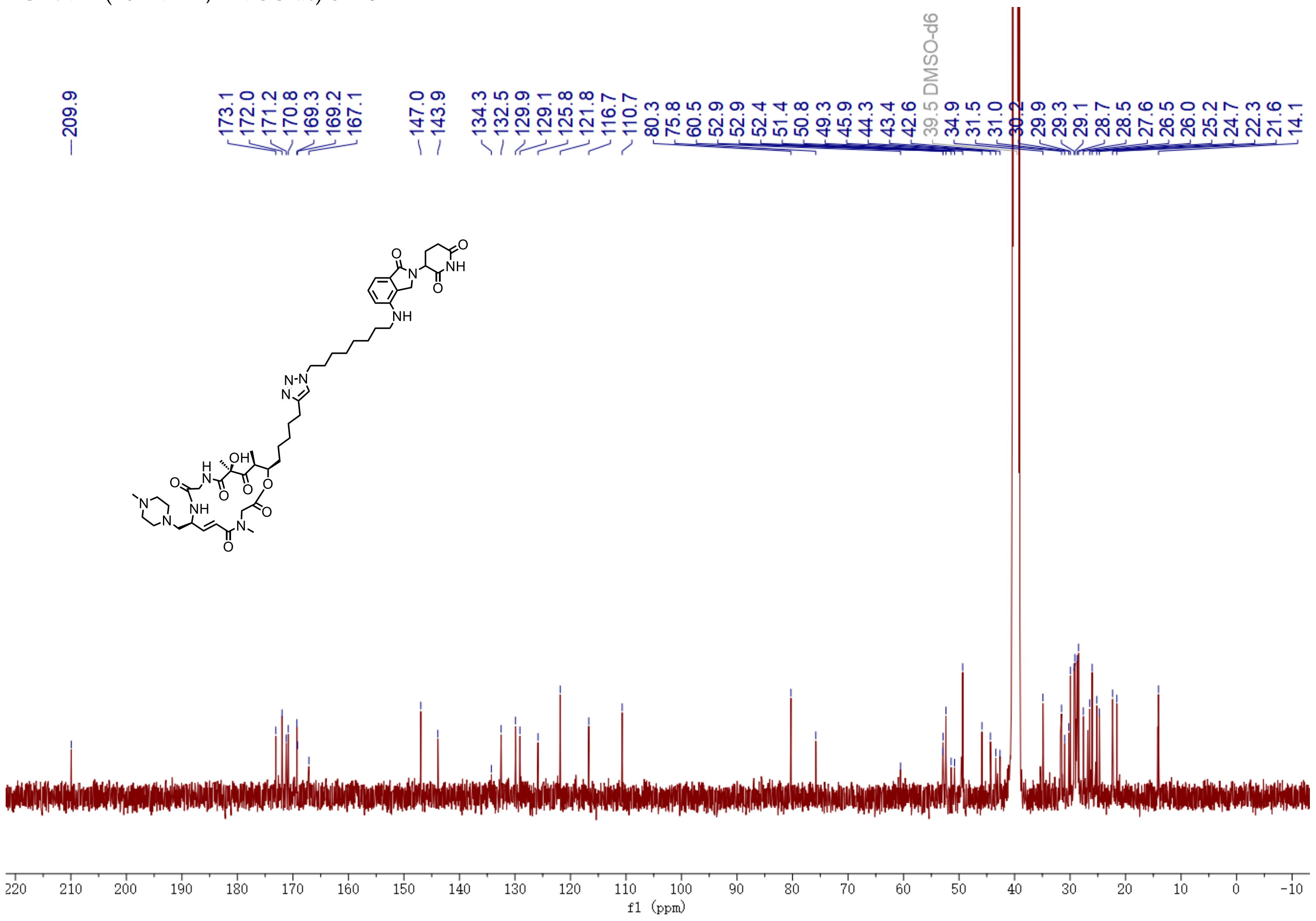

10. LCMS method

Instrument Information: WATERS ACQUITY Arc-Qda

LC Parameters:

Column:XBridge C18 3.5μm 3.0×150mm

Mobile Phase A: H_2O:Acetonitrile:Formic acid（90:10:0.1）

Mobile Phase B: H_2O:Acetonitrile:Formic acid（10:90:0.1）

Gradient: 10% to 100% B in5.0 minutes, 100%B for 7.0 minutes, 100% to 10%B in 0.1 minutes, 10%B for 2.9minutes, then stop.

Flow Rate: 0.8 mL/min

Column Temperature: 30℃

Detector: 254nm and 210 nm

Sample Preparation: 1 mg/mL in Methanol

Injection Volume: 1 μL

Report: Area Normalized Purity

MS Parameters.

Interface: ESI(Positive+Negative)

Scan Range:100-1000(m/z)

11. HPLC chromatograms of representative compounds

HPLC trace of **14**

<Chromatogram>

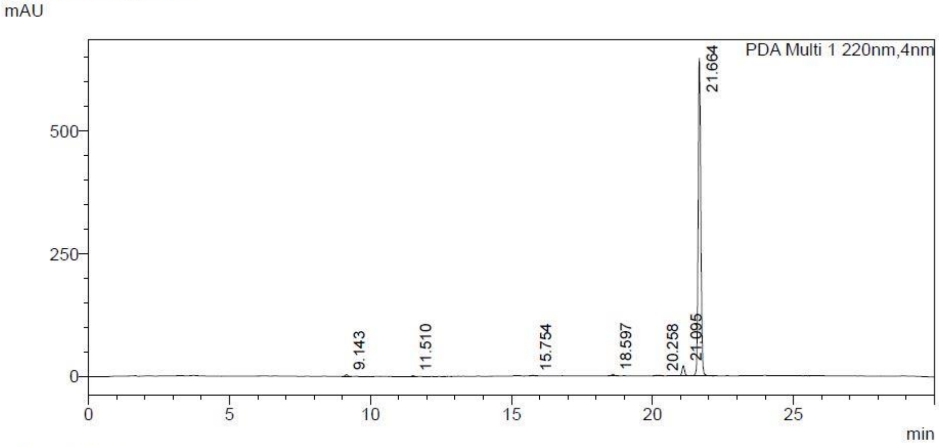

<Peak Table>

PDA Ch1 220nm

Peak#	Ret. Time	Area	Height	Conc.	Unit	Mark	Name
1	9.143	23881	3801	0.539			
2	11.510	10302	1701	0.232			
3	15.754	11304	1771	0.255			
4	18.597	23288	3472	0.526			
5	20.258	13858	1214	0.313			
6	21.095	127967	20515	2.888			
7	21.664	4220916	647255	95.248			
Total		4431516	679730				

HPLC trace of **17**

<Chromatogram>

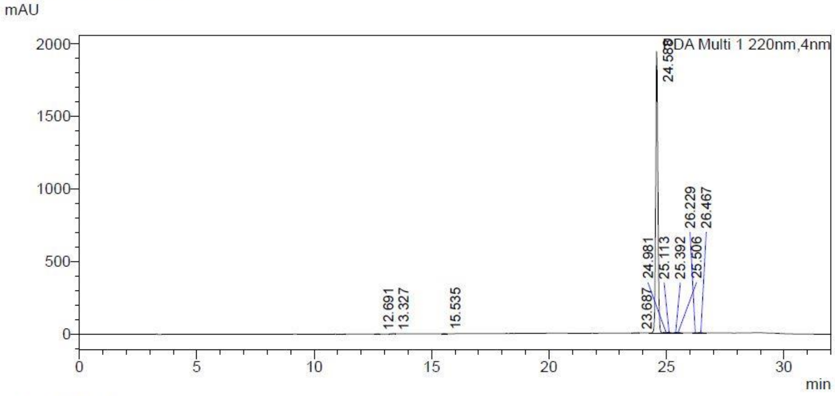

<Peak Table>

PDA Ch1 220nm

Peak#	Ret. Time	Area	Height	Conc.	Unit	Mark	Name
1	12.691	8507	1701	0.068			
2	13.327	7314	1376	0.058			
3	15.535	9323	1554	0.074			
4	23.687	8174	988	0.065			
5	24.588	12323947	1937455	98.310			
6	24.981	42137	5453	0.336		V	
7	25.113	32754	4552	0.261		V	
8	25.392	32709	4932	0.261		V	
9	25.506	39258	5115	0.313		V	
10	26.229	13306	967	0.106			
11	26.467	18326	2116	0.146		V	
Total		12535758	1966209				

HPLC trace of **31**

<Chromatogram>

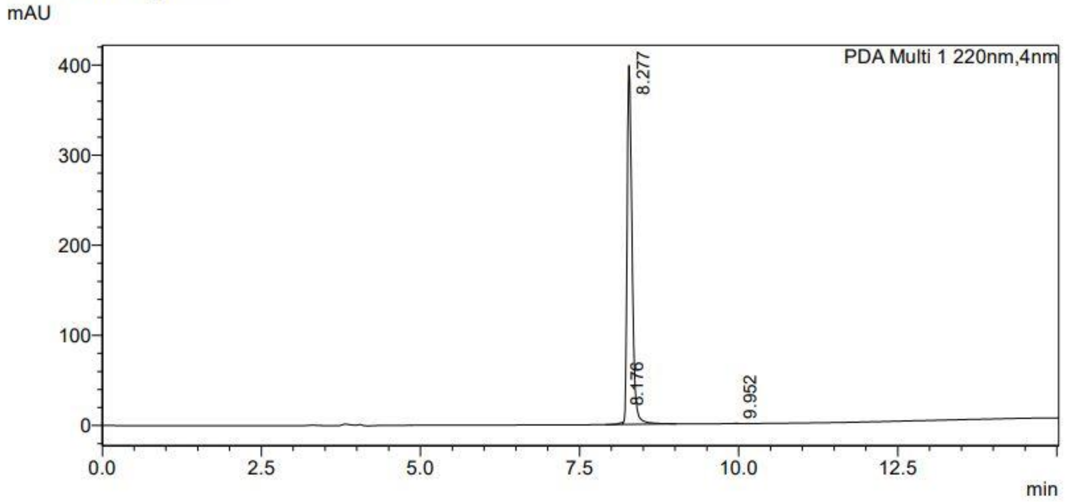

<Peak Table>

PDA Ch1 220nm

Peak#	Ret. Time	Area	Height	Conc.	Unit	Mark	Name
1	8.176	11107	2133	0.512		M	
2	8.277	2155101	398385	99.419		V M	
3	9.952	1483	350	0.068			
Total		2167691	400868				

HPLC trace of **39**

<Chromatogram>

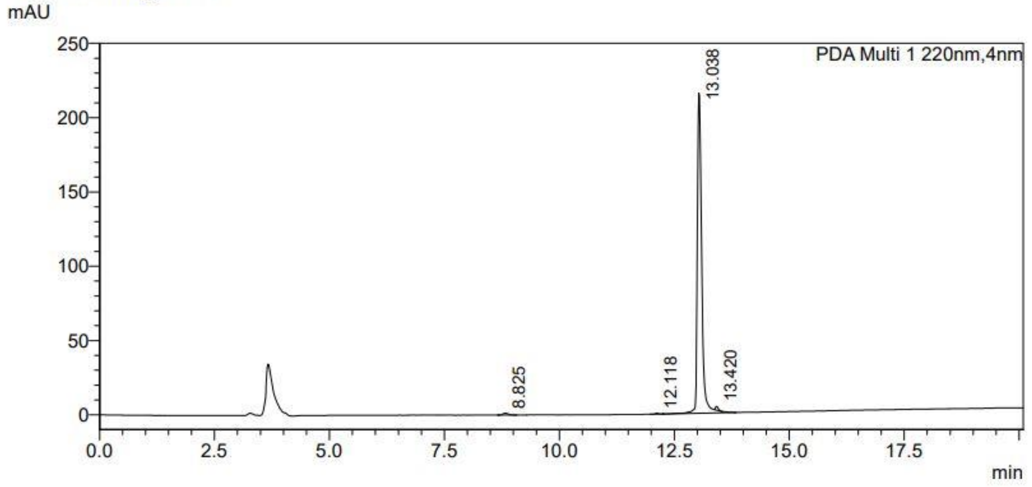

<Peak Table>

PDA Ch1 220nm

Peak#	Ret. Time	Area	Height	Area%	Resolution(USP)
1	8.825	9803	1263	0.681	--
2	12.118	3279	607	0.228	17.487
3	13.038	1415491	215393	98.360	5.531
4	13.420	10517	2505	0.731	2.415
Total		1439091	219768	100.000	

HPLC trace of **40**

<Chromatogram>

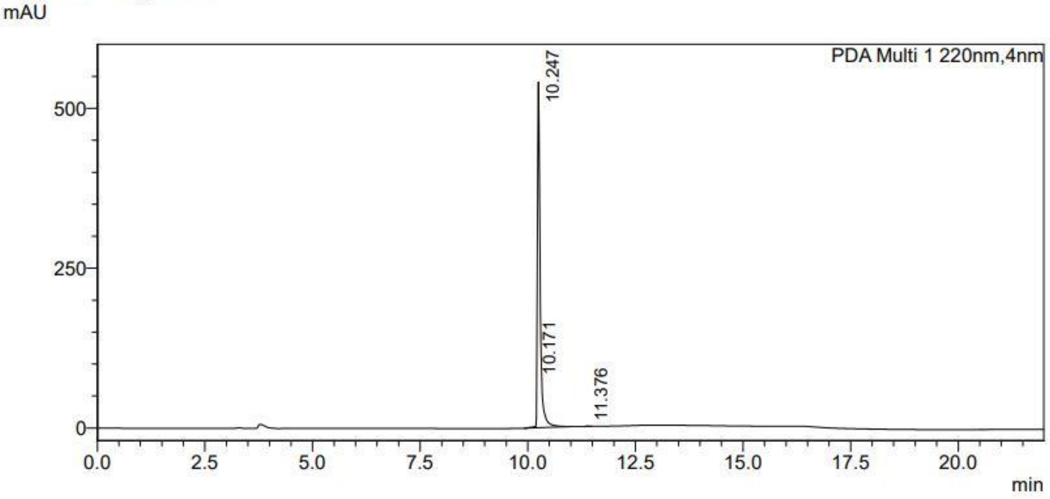

<Peak Table>

PDA Ch1 220nm

Peak#	Ret. Time	Area	Height	Area%	Resolution(USP)
1	10.171	13658	1562	0.561	--
2	10.247	2415750	540747	99.303	--
3	11.376	3293	789	0.135	8.998
Total		2432700	543098	100.000	

12. Bioanalytical Method for Caco-2 Cell Permeability Assay

Chromatographic conditions:

Gradient and flow:
G-1.0 min-10 (for Compound **39**):
0-0.1 min, 3%B; 0.1-0.5 min, 3-100%B; 0.5-0.8 min, 100%B; 0.8-0.81 min, 100-3%B; 0.81-1.0 min, 3%B. Injection volume: 10 μL. Elution rate: 1.0 mL/min.
G-3.0 min-5 (for Compound **31**):
0-0.5 min, 3%B; 0.5-1.8 min, 3-100%B; 1.8-2.3 min, 100%B; 2.3-2.5 min, 100-3%B; 2.5-3.0 min, 3%B. Injection volume: 5 μL. Elution rate: 0.65 mL/min.
G-2.0 min-10 (for Compound BE, BE-NMP, **29**, **40**):
0-0.3 min, 3%B; 0.3-0.8 min, 3-100%B; 0.8-1.2 min, 100%B; 1.2-1.5 min, 100-3%B; 1.5-2.0 min, 3%B. Injection volume: 10 μL. Elution rate: 0.65 mL/min.
Column:
Waters XSelect HSS T3, 2.5μm, 2.1 x 30mm or Waters Xselect PREMIER HSS T3, 2.5μm, 2.1 x 50mm
Mobile phase:
Phase A: water (0.1% formic acid); Phase B: acetonitrile (0.1% formic acid)

MS parameters (for Compound BE, BE-NMP, **29**, **39**, **40**):
LC system: Shimadzu LC30AD, Sciex Triple Quad 6500+ Low Mass, Column temperature: 40 °C, Ion source: Turbo spray IonDrive, Ionization model: ESI, Scan type: MRM, Collision gas: 9 L/min, Curtain gas: 45 L/min, Nebulize gas: 65 L/min, Auxiliary gas: 65 L/min, Temperature: 550°C, Ionspray voltage: +5500 v (positive

MRM) / -4500 v (negative MRM).
MS parameters (for Compound **31)**:
LC system: Shimadzu LC-40D XS, Sciex Triple Quad 6500+ Low Mass, Column temperature: 40 °C, Ion source: Turbo spray IonDrive, Ionization model: ESI, Scan type: MRM, Collision gas: 8 L/min, Curtain gas: 40 L/min, Nebulize gas: 60 L/min, Auxiliary gas: 60 L/min, Temperature: 500°C, Ionspray voltage: +5500 v (positive MRM) / -4500 v (negative MRM).

13. Bioanalytical Method for Solubility Measurement

LC system: Shimadzu
MS analysis: Triple Quad ™ 5500 instrument from AB Inc (Canada) with an ESI interface
Injection volume: 1 μL, 3 μL, 5 μL or 10 μL
For BE-NMP and **29**:
Column: ACQUITY UPLC BEH C18 (2.1 × 50 mmI.D. S-1.7 μm, 12 nm) Column
Mobile phase: 0.1% Ammonium hydroxide solution in water (A) and 0.1% Ammonium hydroxide solution in Methanol (B)

**Time (min)**	0.00	0.30	0.80	1.50	1.60	2.00
**% B**	5	5	100	100	5	5

Elution rate: 0.6 mL/min
For others:
Column: XSelect Hss T3 2.5μm (2.1 × 50 mm) Column XP
Mobile phase: 0.1% formic acid in water (A) and 0.1% formic acid in acetonitrile (B)
Elution rate: 0.65 mL/min

**Time (min)**	0.00	0.60	0.90	1.00	1.40
**% B**	5	100	100	5	5

References

1. Liu, C.; Wang, L.; Sun, Y.; Zhao, X.; Chen, T.; Su, X.; Guo, H.; Wang, Q.; Xi, X.; Ding, Y., Probe Synthesis Reveals Eukaryotic Translation Elongation Factor 1 Alpha 1 as the Anti-Pancreatic Cancer Target of BE-43547A2. *Angew. Chem.* **2022,** 134, e202206953.
2. Fu, H.; Chen, H.; Blazhynska, M.; Goulard Coderc de Lacam, E.; Szczepaniak, F.; Pavlova, A.; Shao, X.; Gumbart, J. C.; Dehez, F.; Roux, B., Accurate determination of protein: ligand standard binding free energies from molecular dynamics simulations. *Nat. protoc.* **2022,** 17, 1114-1141.
